# Supplementary material for: Willingness and perceived ability to pay for Uganda’s proposed national health insurance scheme among informal sector workers in Iganga and Mayuge districts, Uganda: A contingent valuation method
Source: PLOS Glob Public Health. 2026 Mar 11;6(3):e0003603. doi: 10.1371/journal.pgph.0003603 (PMC12978500; doi:10.1371/journal.pgph.0003603)
Supplement: Attachment — Submitted filename: PGPH-D-24-01717_R2.pdf [file pgph.0003603.s004.pdf]

PLOS Global Public Health

Willingness and perceived ability to pay for Uganda's proposed national health insurance scheme among informal sector workers in Iganga and Mayuge districts, Uganda: acontingent valuation method

--Manuscript Draft--

**Manuscript Number:**	PGPH-D-24-01717R2
**Article Type:**	Research Article
**Full Title:**	Willingness and perceived ability to pay for Uganda's proposed national health insurance scheme among informal sector workers in Iganga and Mayuge districts, Uganda: acontingent valuation method
**Short Title:**	Willingness and perceived ability to pay for Uganda's proposed national health insurance
**Corresponding Author:**	Noel Namuhani, MPH<br>Makerere University College of Health Sciences<br>Kampala, UGANDA
**Order of Authors:**	Noel Namuhani, MPH
	Angela N Kisakye
	Suzanne N Kiwanuka, PhD
**Keywords:**	National health insurance scheme, willingness to pay, ability to pay, informal sector, contingent valuation method.
**Abstract:**	Background: Access to healthcare remains a challenge, particularly among informal sector workers in most low-income countries, due to high out-of-pocket expenditures (OOPE). In Uganda, over 28% of healthcare costs are paid out of pocket. In response, Uganda has proposed a national health insurance scheme (NHIs). However, the willingness and ability to pay for the proposed NHIs within the informal sector have not yet been explored in Uganda. This study assessed the willingness and perceived ability to pay for the proposed NHIs, along with its determinants, among informal sector workers in Iganga and Mayuge districts, Uganda.<br>Methodology: A cross-sectional study was conducted in Iganga and Mayuge districts between 10/04/2019 and 23/05/2019. A contingent valuation method using the bidding game technique was used to elicit the willingness to pay (WTP). A total of 853 informal sector workers, including farmers, commercial motorists, fishermen, and traders, were randomly sampled. Seven focus group discussions (FGD) were also conducted. Logistic regression was done to identify the determinants of WTP for the proposed NHIs. Qualitative data was analyzed thematically.<br>Results: The majority 695/853, (81.5%) of the respondents were willing to pay for NHIs; the median WTP was UGX 25,000 (USD 6.8) annually; and 633/853, (74.2%) of the respondents believed that they were able to pay for the NHIs. WTP was significantly associated with being a fisher folk (AOR: 1.70 95%CI: 1.04-2.79, P = 0.035), being in the fourth wealth quintile (AOR: 2.98, 95% CI: 1.56–5.65), not hearing about health insurance (AOR: 0.50 95%CI: 0.23-0.86, P = 0.032), and not having saving group membership (AOR: 0.51, 95%CI: 0.34-0.76, P<0.001). Most of the FGD participants were willing to pay for the proposed scheme; however, some of the participants doubted their ability to pay given their extreme poverty levels and their unstable income.<br>Conclusion: The WTP for the NHIs in the informal sector was high. Therefore, it is viable for the government to extend NHIs to the informal sector. However, building awareness and consideration of extreme poverty levels in setting appropriate premiums should be a priority.
**Opposed Reviewers:**	
**Additional Information:**	
**Question**	**Response**
**Financial Disclosure**	The author(s) received no specific funding for this work.

Powered by Editorial Manager® and ProduXion Manager® from Aries Systems Corporation

Enter a financial disclosure statement that describes the sources of funding for the work included in this submission and the role the funder(s) played. This includes grants and any commercial funding of the work or authors.<br><br>This statement will be typeset if the manuscript is accepted for publication.<br><br>*Please review the submission guidelines and the instructions link below for detailed requirements and guidance.*	
**Competing Interests**<br><br>On behalf of all authors, disclose any competing interests that could be perceived to bias this work.<br><br>This statement will be typeset if the manuscript is accepted for publication.<br><br>*Please review the instructions link below and PLOS Global Public Health's competing interests policy to determine what information must be disclosed at submission.*	The authors declare that they have no competing interests
**Data Availability**<br><br>Before publication, Authors are required to make fully available and without restriction all data underlying their findings. Please see our PLOS Data Policy page for detailed information on this policy.<br><br>A **Data Availability Statement**, detailing where the data can be accessed, is required at first submission. Insert your Data Availability Statement in the box	The dataset analyzed for this study has been uploaded as a supplementary file.

below.<br><br>Please see the data reporting section of our submission guidelines for instructions on what you need to include in your Data Availability Statement.<br><br>This statement will be typeset if the manuscript is accepted for publication.<br><br>*PLOS allows rare exemptions to address legal and ethical concerns. If you have legal or ethical restrictions, please use the box below to detail these in full sentences for the Journal team to consider.*	

Date: 21st /July/2024

The Editor,
PLOS Global Public Health

Dear Sir/Madam,

Re; **Submission of Manuscript**

I wish to submit our manuscript entitled **"Willingness and Perceived ability to pay for Uganda's Proposed National Health Insurance Scheme among Informal Sector workers in Iganga and Mayuge districts, Uganda: A Contingent Valuation Method."** for consideration for publication.

As Uganda plans to introduce a National Health Insurance scheme, it is critical to consider the enrolment of the informal sector who constitute the largest proportion of the labor force to ensure sustainability of the scheme. However, it is challenging to ensure that this large group enroll and contribute to the scheme. Their willingness and ability to pay remain unexplored in Uganda. This study aimed at assessing the Willingness and ability to pay for the proposed Uganda's National Health insurance scheme among the informal sector workers in Uganda.

This paper provides information which will be used by the Uganda ministry of Health and other countries in designing strategies for ensuring maximum participation especially among the informal sector workers. This will also inform setting and collection of premiums for the NHI.

Therefore, we believe that being published in this journal will give this paper a broader visibility and exposure of our work than in any other journal. This paper will be of interest to readers in field of health systems financing and reforms.

We confirm that this work is original and has been approved by all the authors for publication. It has not been published elsewhere nor is it currently under consideration for publication elsewhere.

Thank you for your consideration.

Sincerely,

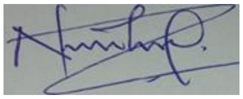

Noel Namuhani, Research associate, Makerere University School of Public Health

Date: 4th /May/2025

The Editor,
PLOS Global Public Health

Dear Sir/Madam,

Re: Address of Comments for the manuscript PGPH-D-24-01717R1

We thank you and the reviewers for the time taken to review our manuscript. We appreciate all the comments and suggestions given. Below is how we attempted to address them and improve the paper.

**Comment**	**How it was addressed**
Language editing	The entire manuscript has been edited.
Reviewer #2: The study aimed to explore the underlying factors influencing willingness and ability to pay for NHI in Uganda. The topic is relevant for health system reforms towards achieving UHC in Uganda. Authors have addressed the majority of key issues raised in my previous reviews. However, there are still a few issues requiring authors' attention:	We appreciate the comments and the second review of our paper. We have looked into the persistent issues raised and addressed them as below.
Although authors have tried, I still think the discussion section is not sufficiently nuanced in a manner that will situate in global body of knowledge for international relevance. Also, the statement included on implication of the study results for policy and practice woefully superficial. I suggest authors elaborate to include actionable recommendations based on the study findings, targeted at various levels of Uganda's health systems.	A sub section on implications for policy is included where we highlight some of the actionable recommendations. The entire discussions has been revised.
In the conclusion, results is still being presented and discussed/interpreted, instead of making concluding remarks based on the interpreted results. I believe the conclusion could still be strengthened.	*This has been rewritten by minimizing the one repeating the results. It is a bit challenging to give concluding remarks without giving an overall picture of our results and how we interpret them.*
Review #3	

Reviewer #3: Avoid unnecessary capitalization in the heading. Do not use willingness to pay and WTP concurrently. Resort to the acronym after first use. OOPE was not defined on first use. Revise that.	*The overall grammar has been checked.*<br>*The capitalizations have been dealt with in the subheadings.*<br>*Acronyms have been used for WTP, and other after first defining on first mention.*<br>*OOPE has been defined on first use in the abstract*
Contrast the following statement with the burden of diseases in these countries and how that creates a paradox. "According to the World Bank, direct modes of payment for54 health care services constitute 40% of health care expenditure in developing countries (4)	*This has been done. Thanks*
The information on page 10, lines 163 to 166, is repetitive	*This has been deleted.*
How was the semi-structured questionnaire developed? Adapted/adopted? Often, researchers have problems integrating results in mixed-method studies. What type of mixed-methods design was employed? How did that influence the integration of both quantitative and qualitative data?	*The tool was developed by the authors and this has been indicated.*<br>*The type of mixed methods study had already been indicated under the section of study design. For emphasis, this was convergent parallel where quantitative and qualitative data were collected simultaneously and hence we present the findings co currently too. This has also been indicated under the section of data collection.*
. Table 2: The classification of the religion is confusing. Catholicism, Protestantism, etc. are not religions but denominations or variants within the Christian fraternity.	*According to Uganda Bureau of statistics, that is how religion is classified. However, to avoid confusion to the global audience, we have changed the subheading to religious denominations. This has been changed in table 2.*
Could you correct this error?Line 264-266: "gave WTP zero" is grammatically incorrect. Should be "had a zero WTP."	*Thank you. We have changed this.*

We hope these revisions fully address your concerns and strengthen the manuscript's clarity, relevance, and contribution to literature.

Thank you for your consideration.

Sincerely,

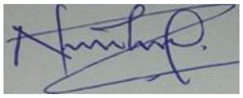

Noel Namuhani, Research associate, Makerere University School of Public Health

Manuscript

Click here to access/download;Manuscript;WTP for the Proposed NHIS Manuscript 25 July 2024.docx

Willingness and Perceived ability to pay for Uganda's Proposed National Health Insurance Scheme among Informal Sector workers in Iganga and Mayuge districts, Uganda: A Contingent Valuation Method.

Noel Namuhani[1*], Angela Kisakye[1, 2] and Suzanne N Kiwanuka[1]

Institutional affiliations

[1]Makerere University School of public health, Department of Health policy planning and management, Uganda

[2]African Field Epidemiology Network, Lugogo House, Plot 42, Lugogo By-Pass

*Corresponding Author

Email: nnamuhani@musph.ac.ug

Abstract

Background: Access to health care remains a challenge, especially among the informal sector workers in most low-income countries, due to high out-of-pocket (OOP) expenditures, with Uganda spending over 28.0% out of pocket on health care. In response, Uganda has proposed a national health insurance scheme (NHI). However, the willingness and ability to pay for the proposed NHI scheme within the informal sector have not yet been explored in Uganda. This study assessed the willingness and perceived ability to pay for the proposed NHI scheme and its determinants among the informal sector workers in Iganga and Mayuge districts, Uganda.

Methodology: A cross-sectional study was conducted in Iganga and Mayuge districts between 10/04/2019 and 23/05/2019. A contingent valuation method using the bidding game technique was used to elicit the willingness to pay (WTP). A total of 853 informal sector workers, including farmers, commercial motorists, fishermen, and traders, were randomly sampled. Seven focus group discussions (FGD) were also conducted. Logistic regression was done to identify the determinants of willingness to pay for the proposed NHI scheme. Qualitative data was analyzed thematically.

Results: The majority 695/853, (81.5%) of the respondents were willing to pay for NHI; the median WTP was UGX 25,000 (USD 6.8) annually; and 633/853, (74.2%) of the respondents believed that they were able to pay for the health insurance. Willingness to Pay was significantly associated with being a fisher folk (AOR: 1.70 95%CI: 1.04-2.79, P = 0.035), being in the fourth wealth quintile (AOR: 2.98, 95% CI: 1.56–5.65), not hearing about health insurance (AOR: 0.50 95%CI: 0.23-0.86, P = 0.032), and not having saving group membership (AOR: 0.51, 95%CI: 0.34-

0.76, P<0.001). Most of the FGD participants were willing to pay for the proposed scheme; however, some of the participants doubted their ability to pay for the scheme given their high poverty levels and their unstable income.

Conclusion: The willingness to pay for health insurance in the informal sector is high. Therefore, it is viable for the government to extend NHI to the informal sector. However, awareness building and due consideration of high poverty levels in setting appropriate premiums should be a priority.

Key words: National Health insurance scheme, Willingness to pay, Ability to pay, informal sector, Contingent Valuation method.

Introduction

Access to health care remains a big challenge, especially among the informal sector in most low-income countries (1, 2), mainly due to high out-of-pocket (OOP) expenditures (3). This put about 150 million people at risk of catastrophic healthcare costs annually, driving over 100 million people into poverty worldwide (3). According to the World Bank, direct modes of payment for health care services constitute 40% of health care expenditure in developing countries (4).

In Uganda, although the national health accounts reported a reduction in the out-of-pocket expenditure on health as a share of current health expenditure (CHE) from 38.6% in the financial year 2018/2019 to 27.4% in the financial year 2020/2021, the OOPE remains significantly high (5). Accessing healthcare is associated with several expenses, including transport, drugs,

diagnostics, and consultancy fees, among other expenses related to personnel and capital costs (6).

In Uganda, over 80% of the labor force belongs to the informal sector (7). These include peasant farmers, commercial cyclists, fishermen, and those employed in unregistered or small-scale enterprises. It is estimated that 59% of the people in the informal sector in Eastern Uganda cannot afford to access quality health care due to high poverty levels and OOP for health care (8).

Globally, the implementation of health insurance schemes is one of the major responses to addressing out-of-pocket payments for health care (3, 9) in low and middle-income countries. Health insurance has the ability to reduce financial barriers to health care access and protect individuals and families against the risk of unpredictable health care expenditures, hence aiding the attainment of UHC (10). This has improved access to healthcare, especially in developed countries.

High coverage and enrollment in the health insurance scheme are critical for the success and sustainability of the scheme. However, enrolment in health insurance schemes remains low, ranging from 2% to 40% in most African countries (11-13). The poor enrolment rates in the different countries have been attributed to a number of factors, including low incomes, large household sizes, long distances to health facilities, limited awareness, poor quality of health care services, inappropriate benefit packages, a lack of trust in the systems, and high illiteracy levels, especially in the informal sector (14-16). The informal sector workers are sometimes of low socioeconomic status compared to the formal sector workers, hence having a lower ability to pay

for health insurance (16). Furthermore, the informal sector is not well organized; they have unpredictable incomes, making it difficult to enroll and register them into the NHI scheme and collect regular contributions from them (13, 17).

Uganda is in the process of developing a NHI scheme to address the high OOP and access to services. However, given the high poverty levels among Ugandans and more so in the informal sector, the policy question of how much money people are willing to spend and their ability to pay remains unanswered. Eastern Uganda is the poorest region in Uganda, with 35.7% of most households living below the poverty line (7). The willingness to pay for the proposed NHI scheme, especially within the informal sector, which makes up 80% of Uganda's population, is critical to the achievement of equity in access to care and the core risk pooling function of a health insurance scheme. This would translate into high enrollment rates, which foster the financial sustainability of the schemes (10).

The national health insurance bill, 2019, proposes that the scheme will be mandatory for all Ugandans, and each person is to pay a premium that will be determined by the board. The bill was passed by parliament in 2021 but was not assented to by the president due to disagreements among stakeholders who were not consulted. As of 2024, the bill is being discussed by the Ministry of Health and stakeholders before being re-tabled in the Parliament of Uganda.

There is a paucity of information on the willingness and ability to pay for the proposed NHI, especially within the informal sector. Therefore, this study aimed at documenting the willingness to pay and the perceived ability to pay for the proposed NHI and factors that influence willingness

to pay for the proposed NHI scheme among the informal sector workers. This study's findings will be used by the MOH in determining affordable premiums and how they should be paid.

Materials and Methods

Study design: This was a cross-sectional study design that involved mixed methods of data collection. The study was conducted from 10/04/2019 to 23/05/2019. This study adopted a contingent valuation method (CVM) using the bidding game technique to directly elicit or measure willingness to pay for a hypothetical (not yet on the market) health insurance package (18). This approach has been used because it has been proven to be an effective way of assessing willingness to pay with minimal bias for goods that are not presently available on the market. The CVM creates a hypothetical marketplace in which no actual transactions are made; hence, this approach has been successfully used for commodities that are not exchanged in regular markets. This approach makes the assumption that people have had no previous experience buying the health service that is going to be put on the market and instead asks people their willingness to pay on the basis of their expectations and the benefits attached to the service (19). In the absence of markets for public goods, this method presents consumers with hypothetical markets in which they have the (hypothetical) opportunity to buy the good in question. Since the elicited WTP values are contingent upon the particular hypothetical market described to the respondent, this approach is called the contingent valuation method (20).

Study setting: The study was conducted in the Iganga and Mayuge districts of eastern Uganda. Iganga and Mayuge districts were selected purposively because they are the most poverty-hit districts in the country and 80% of the population lives in rural areas. They also have 80–85% of

the population belonging to the informal sector (7). Iganga District is bordered by Kaliro District to the north, Bugweri District to the east, Mayuge District to the south, Jinja District to the southwest, and Luuka District to the west. With a total population of 504,197 people, 9.9% of the households are 5 km or more from the nearest health facility, and 19.9% are near a public health facility (UBOS, 2016). It is composed of 3 counties, 8 sub counties, 66 parishes, and 395 villages. The major economic activity is farming. Mayuge district is located in south-eastern Uganda with a population of 473,2394 and a growth rate of 3.5%, and it has thirteen subcounties. The major economic activities are farming and fishing. Mayuge district is bordered by Lake Victoria to the south, as well as Jinja and Bugiri districts. It has a total of 33 landing sites where fishing activities take place.

Study Population: The study targeted the four major categories of informal sector workers: 1) farmers; 2) fishermen; 3) commercial cyclists; and 4) the business community (traders and market vendors) in the study area. The informal sector was chosen because they make up the largest (80%) portion of the population, without which the sustainability of the scheme will be questionable.

Sample Size: The sample size was calculated using the Kish Leslie formula (21), considering a 95% confidence interval, a 50% expected level of WTP, a 5% level of precision, a design effect of 2, and a 10% non-response rate. This gave a total sample size of 853 informal sector workers. Four focus group discussions were conducted with women (four FGDs), and three FGDs were conducted with men (in the informal sector). This number depends on the level of information saturation. This was determined by doing preliminary analysis while still in the field.

Sampling Procedure: Stratified random sampling was used in this study. For Iganga district, the informal sector workers were divided into three major categories (strata): farmers, commercial cyclists, traders/market vendors. In Mayuge district, the informal sector workers were divided into four categories: fishermen, farmers, commercial cyclists, and market vendors. A random sample from each stratum was taken in a number proportional to the stratum's size when compared to the population. In Iganga district, two subcounties were randomly selected, and five villages from each subcounty were also randomly selected. A proportionate sample of 286 households from the 10 villages was selected, and the household heads were systematically selected for interviews. Seven *bodaboda* stages located along main roads and junctions were randomly selected, and 10 cyclists available at the time of the study were randomly selected to participate in the interviews. A total of 84 traders were selected systematically from the two main markets for interviews. In Mayuge district, a total of 10/33 landing sites were randomly selected, and 248 fishermen were selected from the 10 landing sites proportionate to size. Sixty-two traders were randomly selected from three main markets systematically, and 62 bodaboda cyclists were randomly selected from six stages located along main roads in Mayuge town (Table 1). Men and women from each of the categories were purposively selected for the focus group discussions.

Table 1: Sampling procedure per category of informal sector

**Iganga district (**Iganga district planning office, 2018)				
**Category**	**Farmers/peasants**	**Commercial cyclists**	**Traders/vendors**	**Fisherfolk**
Proportion in the district	0.65	0.16	0.19	
Sample size	(0.65*440) =286	(0.16*440) =70	(0.19*440) =84	-

Selection procedure	2 sub counties (SC) and 5 villages from each SC were randomly selected. Households proportionate to size selected.	7 *Boda boda* stages in the town were randomly selected, cyclists on stage randomly selected to be interviewed	Traders were systematically selected for the interviews two main markets	-
Mayuge district				
**Category**	**Farmers/peasants**	**Commercial cyclists**	**Traders/vendors**	**Fisherfolk**
Proportion in the district	0.1	0.15	0.15	0.6
Sample size	(0.1*413) =41	(0.15*413) =62	(0.15*413) =62	(0.6*413) =248
Selection procedure	One sub county and two villages were randomly selected, the number of households were determined proportionate to size.	6 *Boda boda* stages in town were randomly selected, later cyclists on stage at that time were randomly selected to be interviewed	Traders were systematically selected from 3 main markets	10/33 landing sites were randomly selected. Fishermen at each site were randomly selected, proportionate to size
**Total samples**	**327**	**132**	**146**	**248**

Data collection methods and tools

The willingness to pay was assessed using a contingent valuation method using the "bidding game" technique. The bidding game was used to determine the maximum premium that a respondent is willing to pay for the proposed NHI plan. A bidding game technique was chosen because of its ease of implementation and its minimal biases. In addition, many studies have found the bidding game to be very reliable (22, 23). The respondent was asked if he or she was willing to pay the starting bid. If the respondent agreed, the interviewer would raise the bid by 10% of the first bid and again ask if the respondent was willing to pay the new bid. The

interviewer continued to present the different bids until the respondent expressed an unwillingness to pay. On the other hand, if the respondent expressed unwillingness to pay the starting bid, the interviewer lowered the bid by 10% and repeated the query. This was continued until a bid was reached where the respondent was willing to pay. The starting bid was 20,000 UGX (5.4 USD), since this was the average premium for beneficiaries in the existing community-based health insurance schemes in Luweero, Western Uganda, and Jinja. The proportion of those willing to pay, the average, and the median amount of money people were willing to pay were computed. The perceived ability to pay was measured by asking respondents whether they were able to pay premiums for health insurance per year and how much they could afford without foregoing other basic needs.

A semi-structured questionnaire was used to collect quantitative data such as sociodemographics, WTP, perceived ability to pay, and associated factors from informal sector workers. The data collection tools were translated to the local language and then back translated to English to check whether the translated questions still held informational validity. The principal investigator recruited and trained research assistants so that they were familiar with the statement of the problem, objectives of the study, sampling procedure, data collection tools, and plan for data collection. Research assistants were oriented to the bidding game technique for assessing WTP. They were also taught the meaning of health insurance and how it intends to be operationalized in the country. Research assistants were also trained on the basic interview techniques, such as asking questions in a neutral manner, not showing by words or actions what answers were being expected of the study participants, and how to record answers, especially from open-ended questions, without interpreting them. The data collection tools were pre-

tested in the neighboring district, Bugweri district. This was done to test the validity and ease of application of the tools. Filled electronic forms were checked at the point of data collection for completeness, and those found incomplete were completed before the respondent was discharged. The data were cleaned and edited by the research assistants before they submitted the complete forms to the server.

Focus group discussion (FGD) guides were also used to collect data from informal sector workers to understand the determinants of willingness to pay and their perceived ability to pay for the NHI. The FGDs were audio-taped. The FGD was composed of 6–8 participants. The discussion had a moderator and a note-taker, and the discussions lasted for an average of one hour. These were conducted in the local language, and these were audio recorded.

Data management and analysis: The quantitative data were downloaded from the server and imported into STATA 14 for cleaning and analysis. Descriptive statistics, including mean, median, standard deviations, frequencies, and percentages, were obtained. The information was presented in frequency distribution tables. Principal component analysis was done to generate wealth quintiles using the demographic health survey categorization. The wealth quintiles were generated based on nine household items, which included possession of a car, motorcycle, bicycle, radio, television, mobile phone, piece of land, owning a house, and having animals. Inferential statistics were obtained using logistic regression. This was done in two stages: 1) bivariate analysis was done to determine the potential variables associated with willingness to pay for health insurance. Crude odds ratios at a 95% confidence interval were obtained to measure the potential association. 2) A multivariate analysis was done to determine the actual

factors associated with WTP. All variables that were statistically significant at bivariate analysis and the important factors known in the literature to be statistically significant, such as socio-economic status, occupation, and potential confounders like residence, were included in the model. Variables were tested for multicollinearity before being included in the model. The model was built step-by-step while testing it using lfit until the best model was obtained. Variables that remained statistically significant at 95% confidence were considered the factors associated with WTP for health insurance. Adjusted odds ratios were obtained as measures of association.

Qualitative data was analyzed manually using a thematic-inductive approach. The recordings were transcribed verbatim and translated from the local language (Lusoga) to English. Transcripts were read by more than two independent analysts who identified codes, sub-themes, and then organized them into themes. The information was triangulated with the quantitative data findings to gain a deeper understanding.

Ethics statement

The study was approved by the Makerere University, School of Public Health Higher Degrees, Research and Ethics Committee (HDREC). At the district level, permission to conduct the study was obtained from the local leaders and the District Health Office. Permission was also sought from subcounty chiefs and local council one chairpersons. The informed written consent of each individual participant was obtained at the start of the study. Confidentiality and anonymity were maintained by the use of codes.

Results

Background Characteristics of the Respondents

Of the 853 respondents who participated in the study, 327 (38.3%) were peasant farmers, 248929.1%) were fishermen, 146 (17.1%) were business people, and 132 (15.5%) were commercial cyclists. The mean age of the respondents was 37.0 years (SD ± 11.3). The majority 667/853 (78.2%) of the respondents were males, 783/853 (91.8%) of the household heads were males, and 618/853 (72.4%) resided in rural areas. The average household size was 5 persons. The median monthly income was UGX 150,000 (USD 40.5). Only 158/853 (18.5%) were in the wealthiest quintile, while 349/853 (41.0%) of the households were in the second and poorest wealth quintiles (Table 2).

Table 2: Background Characteristics of the Respondents

Variable	Frequency (N=853)	Percentage (%)
**Sex of respondent**		
Male	667	78.2
Female	186	21.8
**Sex of the household head**		
Male	783	91.8
Female	70	8.2
**Residence**		
Rural	618	72.4
Urban	235	27.6
**Marital status**		
Married	694	81.4
Single	85	10.0
Separated	48	5.6
Widowed	26	3.0
**Age category**		
18-25	124	14.5
26-35	303	35.5
36-45	239	28.0
46-55	126	14.8
56 above	61	7.2

**Education level**		
None	51	6.0
Primary	445	52.2
Secondary	280	32.8
Tertiary	77	9.0
**Religion**		
Muslim	304	35.6
Protestant	276	32.4
Catholic	194	22.7
Born again	53	6.2
SDA	26	3.1
**Occupation**		
Farmer	327	38.3
Fishermen	248	29.1
Business	146	17.1
Bodaboda	132	15.5
**Wealth quintiles**		
Poorest (<-1.39)	172	20.2
Poor (-1.40 - -0.50)	177	20.8
Moderate (-0.51 -0.29)	163	19.1
Wealthy (0.30 -1.23)	183	21.5
Wealthiest(1.24-4.14)	158	18.5

Willingness to Pay for the Proposed NHI

The majority 695/853 (81.5%) of the respondents were willing to pay for the proposed NHI. Of those willing to pay, the median willingness to pay was UGX 25,000 (USD 6.8) while the mean WTP was UGX 28,950 (USD 7.8) per person, inclusive of 4 dependents per year. Only 3.8% protested contributing to the scheme, and 126/853 (14.7%) gave WTP zero because they believed they were too poor to pay for national health insurance (Table 3).

Qualitatively, most of the FGD participants were also willing to make contributions for health insurance. However, others resented making contributions, as noted below:

> *"Sincerely, the government is meant to provide all the medical services. So, there is no way that you can lie to us and say that the government can't provide those services..."* **(FGD male-fishermen-Mayuge)**

A number of FGD participants were not willing to pay because of poverty, while others criticized the government's misuse of taxes collected from the tax payer on salaries for officials rather than on health services, as noted in the quotes below.

> *"The fact is that poverty is the biggest hindrance. Because at times you can fall sick, yet you don't have money, and you resort to borrowing from colleagues who may not even be able to help you out because they also don't have. So generally, I disagree with paying for health insurance."* **(FGD-Business Women, Mayuge District)**

"I am not willing to pay because we are always charged a lot of tax to register our businesses. That is all tax. Where does that tax go? Let them reduce the salary of Members of Parliament so that part of it is utilized to buy medicine. Members of Parliament have no importance in our area." (**FFGD-Male-fishermen-Mayuge district)**

Willingness to Pay with Copayments:

A copayment is a cost-sharing arrangement where one covers a portion of the costs out of pocket each time one visits the health facility. Less than half, 309/695 (44.5%) of the respondents, were willing to co-pay. The mean co-pay amount was UGX 2,650 (USD 0.7), with a median of UGX 2000 (USD 0.5), each time someone accessed services. The average maximum amount of money that respondents were willing to pay with the copayment was UGX 23,000 (USD.6.2) and the median was UGX 20,000 (USD.5.4) (Table 3).

Table 3: Willingness to Pay for the Proposed NHI annually

**WTP**	**Frequency (%)**	**Mean**	**Median**
Protests (WTP=0)	32 (3.8)	0	0
WTP=0	126 (14.7)	0	0
WTP>0	695(81.5)	**28,950**	**25,000**
Total	853 (100.0)	23,600	20,000
**Copayment scenario**			
Amount willing to copay	309/695(44.5)	**2,650**	**2,000**
WTP with co payment	309/695(44.5)	23,000	20,000

Notes; *Protests-those protested attaching valuing to HI (WTP=0 but it has no economic sense). WTP=0 means willingness to pay is actually zero. WTP>0 that it is positive -WTP is above zero.*

Qualitatively, most of the FGD participants were not in support of copayments, as expressed by one of the FGD participants.

"I also do not support the idea that we pay money each time we visit the health facility, especially when we are paying every year. This will make people unable to access the services, and hence the problem of access to services will not have been solved. People are already poor, and that will mean people will continue to sell their property to go to health facilities." (FGD Male Farmer)

Preferred Mode of Payment

Most (54.1%) respondents preferred to pay annually, while 21.0% preferred to pay per harvesting season. Only 13.1% wanted to pay according to their monthly income, with the majority (63.1%) preferring to pay 1 percent of their monthly income, followed by those who were willing to pay 5%.

Perceived Ability to Pay for the Health Insurance Scheme

More than half 633/853 (74.2%) of the respondents believed that they were able to pay for health insurance, with a mean of UGX 23,000 (USD.6.2) and median of UGX 20,000 (USD.5.4) per person per year.

Regarding the perceived ability to pay, most of the FGD participants noted that most of the people are poor and most likely may not be able to make periodic subscriptions, especially if the money is raised more than what people earn. This was attested to, as indicated below:

"Sincerely speaking, some people cannot afford to pay money; for instance, you may find a very old person who is no longer earning anything, yet they also need health care." **(FGD-Boda-boda-Iganga district)**

Another FGD participant, who was a business lady, noted that

> *"I support the idea. But not very much because of the issue of affordability. Not everyone can afford to pay, and note that we earn differently. Because some of us play the role of mother and father in the family, there are a lot of requirements to provide."* **(FGD Business Women, Iganga)**

Furthermore, most of the participants noted that setting premiums should be based on people's income and ability to pay, as noted below:

> *"Since everyone earns differently, the charges should depend on how much someone earns. I could be in a position to afford UGX 50,000, but yet my colleague cannot at all since people in the village are used to getting free medicine. When some people are asked to pay just 500shs for drugs, they will consider it unworthy because they know that drugs/medication are freely given. So, if possible, we charge according to people's income."* **(FGD: business women, Iganga district)**.

Determinants for level of Willingness to Pay for Health Insurance Scheme

At bi-variable analysis, wealth quintiles, having savings group membership, having a family member with chronic illness, use of traditional medicine, and hearing about health insurance were statistically significant for willingness to pay for health insurance. The odds of being willing to pay when in the fourth wealth quintile were 2.88, compared to the odds of being willing to pay among those in the poorest category (COR: 2.88, 95% CI: 1.60–5.18, P<0.001). Not being in the saving group reduced the chances of being willing to pay for health insurance by 42%

compared to those who had saving group memberships (COR: 0.58, 95% CI: 0.33-0.69, P<0.001) (Table 4).

After adjusting for any confounding factors, occupation, wealth, saving group membership, use of traditional medicine, and having heard about health insurance were significantly associated with the level of willingness to pay for the proposed health insurance scheme.

The odds of fishermen's willingness to pay for health insurance were 1.70 compared to those of farmers after adjusting for other factors (AOR: 1.70, 95% CI: 1.04–2.79, P = 0.035). Respondents in the fourth wealth quintile were 2.98 times more likely to pay for health insurance compared to those in the poorest wealth quintile (AOR: 2.98, 95% CI: 1.56–5.65). Respondents who were not in any saving group were 0.51 times less likely to pay for the proposed health insurance compared to those with saving group membership (AOR: 0.51, 95% CI: 0.34-0.76, P<0.001), and individuals who had never heard about HI were 50% less willing to pay for the proposed National Health Insurance compared to those who had ever heard about health insurance (AOR: 0.50, 95% CI: 0.23-0.86, P = 0.032) (Table 4).

Table 1: Multi variable analysis of factors associated with WTP for NHI scheme.

**Variable**	**Level of Willingness to pay**		**COR (95%CI)**	**AOR (95%CI)**
	**No (n=158)**	**Yes (n=695)**		
**Sex of respondent**				
Male	127(80.4)	540(77.7)	1.0	
Female	31(19.6)	155(22.3)	1.18(0.76-1.81)	1.30(0.80-2.11)
**Residence**				
Rural	110(69.6)	508(73.1)	1.0	
Urban	48(30.4)	187(26.9)	0.84(0.58-1.23)	1.09(0.52-2.26)
**Occupation**				

Farmer	60(38.0)	267(38.4)	1.0	
Business	32(20.2)	114(15.4)	0.80(0.49-1.30)	0.59(0.26-1.35)
Boda boda	25(15.8)	107(15.4)	0.96(0.57-1.61)	1.04(0.50-2.18)
Fishermen	41(26.0)	207(29.8)	1.13(0.73-1.76)	1.70(1.04-2.79) *
**Wealth quintiles**				
Poorest	43(27.2)	129(18.6)	1.0	
Second poorest	32(20.3)	145(20.9)	1.51(0.90-2.53)	1.83(1.05-3.19) *
Middle	35(22.1)	128(18.4)	1.22(0.73-2.03)	1.23(0.70-2.14)
Fourth	19(12.0)	164(23.6)	2.88(1.60-5.18)	2.98(1.58-5.65) *
Wealthiest	29(18.4)	129(18.6)	1.48(0.87-2.52)	1.53(0.81-2.88)
**In saving group**				
Yes	49(31.0)	337(48.5)	1.0	
No	109(69.0)	358(51.5)	0.48(0.33-0.69)	0.51(0.34-0.76) **
**Family member with chronic illness**				
Yes	36(22.7)	241(34.7)	1.0	
No	122(77.2)	576(67.5)	0.56(0.37-0.83)	0.73(0.46-1.14)
**Ability to meet health care**				
Difficult	112(70.9)	571(82.2)	1.0	
Easy	46(29.1)	124(17.8)	0.53(0.36-0.78)	0.68(0.43-1.05)
**Use Traditional Medicine**				
No	59(38.8)	345(51.4)	1.0	
Yes	93(61.2)	326(48.6)	0.60(0.42-0.86)	0.71(0.48-0.92) *
**Ever heard about NHI**				
**Yes**	9(5.7)	83(11.9)	1.0	
**No**	149(94.3)	612(88.1)	0.45(0.22-0.91)	0.50(0.23-0.86) *

Note: ** *p<0.001, *P<0.05*

Discussions

Firstly, this study is unique in its assessment of the level of WTP, perceived ability to pay, and determinants of WTP for the proposed national health insurance scheme among the informal sector workers in Uganda, who constitute 80% of the Ugandan population. These findings are pertinent if the proposed NHI is to achieve adequate coverage. Secondly, the study applied a contingent valuation approach using a bidding game technique to hypothetically elicit the informal sector's willingness to pay. The highlight of this study is the high level of willingness to pay for NHI among informal sector workers, as well as their preferred modes of engagement. These findings provide valuable insights for fine-tuning the proposed NHI.

The level of WTP for the proposed NHI was high, with a median willingness to pay of UGX 25,000 (USD 6.8) and a mean WTP of UGX 28,950 (USD 7.8) per person, inclusive of 4 dependents per year. This level of willingness to pay was higher than the reported WTP in Nepal (71%) and in South Sudan (68%) among the informal sector (13, 24). However, the maximum amount of money people were willing to pay was lower than the 5.5 USD per person per month in Iran, 6.6 USD per person per month in Namibia, and 3 USD in Kenya per month (18, 24, 25). The differences in WTP across the different countries could be due to the differences in the socio-economic status of the different populations in the different countries. The high level of willingness to contribute to health insurance highlights high chances of the scheme being successful. However, the suggested premium being higher than the stated WTP may hinder many from participating in the scheme. But this also brings up the policy question of whether the WTP can sustain the scheme. Thus, there is a need for careful revision of the premiums before Parliament passes the NHI bill.

The ability to pay is another important consideration when planning to implement health insurance, especially among the informal sector, whose incomes are unpredictable. A high

number of participants believed that they could afford to make periodic contributions to the health insurance scheme in this study. This finding further highlights the potential for the NHI to succeed if the informal sector is included. However, the ability to pay needs to be considered when setting premiums, given the fact that less than 20% of the studied population were in the wealthiest category.

Although more than half of the respondents earned more than 100,000 UGX (27 USD) a month, there are substantial sections of the informal sector that will struggle to pay for health insurance, and therefore, these will require significant government subsidies to be included. This further highlights the need to consider the idea of making contributions as a proportion of people's earnings and wealth status because it would make people pay according to their ability and is more equitable than just having a flat figure for all people. Moreover, by virtue of the unpredictable nature of their income, informal sector workers expressed the desire to have premiums synchronized to earning cycles such as harvest seasons. This implies that the NHI will have to explore flexible premium schedules for informal sector workers in order to enhance their uptake of the scheme. In Ethiopia, making premium systems more flexible eased payments (26).

In this study, having a savings group membership increased the likelihood of willingness to pay for the proposed national health insurance scheme. In Asian countries, saving groups were reported to be key in initiating community-based health insurance schemes (27). Savings groups provide members with a secure place to save money, generate a pool fund, and have the opportunity to borrow in small amounts. They also provide affordable basic insurance services and enable the community to meet the premiums(27). Local savings groups have become one of the most famous financial support systems in rural Uganda over the last decade (28). Savings and

burial groups present an opportunity to leverage in enhancing access to health care in rural communities. This implies that as countries plan to initiate NHI, saving groups can become a great resource to tap into to ensure community participation and payment of the premiums for the scheme.

Participants belonging to the highest wealth category were more likely to pay for the proposed national health insurance scheme. This is in agreement with studies conducted in Nigeria, Ethiopia, and Ghana, which have reported that socio-economic factors such as poverty, income, or wealth status have a bearing on the ease or difficulty of enrolling in a given scheme, with high premiums deterring enrollment in schemes (29-31). Similar studies conducted in Sierra Leone and Iran showed that the WTP for the HI scheme depended on the monthly income of the respondent (25, 32) . In Nepal, belonging to a lower wealth quartile was associated with a lower willingness to pay for National Health Insurance Scheme (33). This therefore underscores the need for income generating projects to be implemented within the communities to aid them make contributions for health insurance.

The use of traditional medicine was negatively associated with participation in the health insurance scheme. This is because members who use traditional medicine see no need to utilize modern health care services, making them less likely to pay for health insurance. Studies have shown that the presence and use of alternative medicine and other forms of healthcare, such as herbalists, negatively affects participation in a health insurance scheme since insurance requires regular use of modern medicine. Studies conducted in Cameroon, Burkina Faso, Nigeria, and India have reported that those who use modern medicine are more willing to pay for health insurance than those who use traditional medicine (34-36). In Uganda's pluralistic health system, the

ministry of health and other mandated regulatory bodies need to institute measures that not only regulate the alternative sector but ultimately attract people to use modern medicine as opposed to traditional medicine.

It is not surprising that those who had never heard about health insurance had 50% less WTP compared to those who had prior information about health insurance. Indeed, the level of knowledge on how health insurance operates was very low, and statistical tests of significance could not be used in this study. However, one systematic review and meta-analysis of factors influencing WTP for voluntary contributary health insurance schemes in LMICs has revealed that knowledge and understanding of the functioning of the scheme positively influence the willingness to pay for and participate in the scheme (37, 38). As the Ugandan government moves to implement health insurance, information, education, and communication will be paramount to the success of the proposed scheme.

Study Limitations: As with most contingent valuation studies, the elicitation technique is always subject to bias, and the assignment of the first bid or amount is also biased. However, this was triangulated with an open-ended question and qualitative data. The low level of knowledge about health insurance among respondents was also a limitation for attaching value to the proposed scheme. This was solved by educating the respondent about health insurance before eliciting a willingness to pay.

Conclusions

This study indicated a high level of WTP, and the majority believed they could afford to pay for the health insurance scheme. However, studies on the ability to pay need to be conducted, as

this study is based on the perceived ability to pay for health insurance schemes. The high level of WTP indicates that the informal sector is willing to make a contribution to their health, making it feasible for the government to propose a flexible and affordable scheme within which the informal sector can bear some costs towards their health care. To support the ability to pay, there is a need to build local solidarity groups and strengthen local initiatives to aid poor members.

Declarations

Consent for publication

Not Applicable

Availability of data and materials

The datasets analyzed for this study are available from the corresponding author on reasonable request.

Competing interests

The authors declare that they have no competing interests

Funding

The author(s) received no specific funding for this work

Authors' contributions

NN, SNK, AK conceived the study, NN collected, analyzed and wrote the first draft of the manuscript. SNK and AK provided reviews on all drafts of manuscript. All the authors read and approved the final manuscript.

Acknowledgements

We wish to thank the District Health Officers of Mayuge and Iganga districts for their cooperation, mobilization and ensuring that data collection went on smoothly. We also extend our sincere appreciation to the research assistants and all respondents in Iganga and Mayuge for sparing their time to contribute to this piece of work.

References

1. Sarker AR, Sultana M, Mahumud RA, Ahmed S, Islam Z, Morton A, et al. Determinants of enrollment of informal sector workers in cooperative based health scheme in Bangladesh. PloS one. 2017;12(7):e0181706.
2. Kiwanuka S, Ekirapa E, Peterson S, Okui O, Rahman MH, Peters D, et al. Access to and utilisation of health services for the poor in Uganda: a systematic review of available evidence. Transactions of the Royal Society of Tropical Medicine and Hygiene. 2008;102(11):1067-74.
3. WHO. Health Systems Financing: The Path to Universal Coverage. Geneva: World Health Organization 2010.
4. World Bank. Financing health in low-income countries. Washington DC: World Bank Group; 2010.
5. MoH. National Health Accounts 2018/19-2020/21 Report. 2021.
6. Tashobya CK, Ssengooba F, Cruz VO, Yates R, Murindwa G, McPake B. Health systems reforms in Uganda: processes and outputs. Christine Kirunga Tashobya FS and, Valeria Oliveira Cruz, editors. 2006.

7. UBOS. The National Population and Housing Census 2014 – Main Report. Kampala, Uganda: Uganda Bureau of Statistics; 2016.

8. Twaweza. Health Check: Ugandans' experiences and opinions on health services Uganda: Twaweza; 2019 [Available from: https://www.twaweza.org/go/szw-ug-health-check.

9. Gottret P, Schieber G. Health financing revisited: a practitioner's guide: The World Bank; 2006.

10. Xu K, Evans DB, Carrin G, Aguilar-Rivera AM, Musgrove P, Evans T. Protecting households from catastrophic health spending. Health affairs. 2007;26(4):972-83.

11. De Allegri M, Sanon M, Bridges J, Sauerborn R. Understanding consumers' preferences and decision to enrol in community-based health insurance in rural West Africa. Health policy. 2006;76(1):58-71.

12. Kyomugisha E, Buregyeya E, Ekirapa E, Mugisha J, Bazeyo W. Strategies for sustainability and equity of prepayment health schemes in Uganda. African health sciences. 2009;9(2).

13. Basaza R, Alier PK, Kirabira P, Ogubi D, Lako RLL. Willingness to pay for National Health Insurance Fund among public servants in Juba City, South Sudan: a contingent evaluation. International journal for equity in health. 2017;16(1):158.

14. Nguyen H, Knowles J. Demand for voluntary health insurance in developing countries: the case of Vietnam's school-age children and adolescent student health insurance program. Social Science & Medicine. 2010;71(12):2074-82.

15. Acharya A, Vellakkal S, Taylor F, Masset E, Satija A, Burke M, et al. Impact of National Health Insurance for the Poor and the Informal sector in Low-and Middle-Income Countries.

London: EPPI-Centre, Social Science Research Unit, Institute of Education, University of London; 2012.

16. Basaza R, Criel B, Van der Stuyft P. Community health insurance in Uganda: why does enrolment remain low? A view from beneath. Health Policy. 2008;87(2):172-84.

17. Jowett M, Kutzin J, Organization WH. Raising revenues for health in support of UHC: strategic issues for policy makers. World Health Organization; 2015.

18. Gustafsson-Wright E, Asfaw A, van der Gaag J. Willingness to pay for health insurance: An analysis of the potential market for new low-cost health insurance products in Namibia. Social science & medicine. 2009;69(9):1351-9.

19. Russell S, Fox-Rushby J, Arhina D. Willingness and ability to pay for health care: a selection of methods and issues. Health Policy and Planning. 1995;10(1):94-101.

20. Mitchell RC, Carson RT. Using surveys to value public goods: the contingent valuation method: Rff Press; 2013.

21. Kish L. Survey sampling. Inc., New York.: John Wiley and Sons; 1965.

22. Whittington D, Smith VK, Okorafor A, Okore A, Liu JL, McPhail A. Giving respondents time to think in contingent valuation studies: a developing country application. Journal of Environmental Economics and Management. 1992;22(3):205-25.

23. Dong H, Kouyate B, Cairns J, Sauerborn R. A comparison of the reliability of the take-it-or-leave-it and the bidding game approaches to estimating willingness-to-pay in a rural population in West Africa. Social Science & Medicine. 2003;56(10):2181-9.

24. Barasa EW, Mwaura N, Rogo K, Andrawes L. Extending voluntary health insurance to the informal sector: experiences and expectations of the informal sector in Kenya. Wellcome open research. 2017;2.

25. Nosratnejad S, Rashidian A, Mehrara M, Sari AA, Mahdavi G, Moeini M. Willingness to pay for social health Insurance in Iran. Global journal of health science. 2014;6(5):154.

26. Habte A, Tamene A, Ejajo T, Dessu S, Endale F, Gizachew A, et al. Towards universal health coverage: The level and determinants of enrollment in the Community-Based Health Insurance (CBHI) scheme in Ethiopia: A systematic review and meta-analysis. PloS one. 2022;17(8):e0272959.

27. Shaikh BT, Noorani Q, Abbas S. Community based saving groups: an innovative approach to overcome the financial and social barriers in health care seeking by the women in the rural remote communities of Pakistan. Archives of Public Health. 2017;75(1):57.

28. Mutebi A, Muhumuza Kananura R, Ekirapa-Kiracho E, Bua J, Namusoke Kiwanuka S, Nammazi G, et al. Characteristics of community savings groups in rural Eastern Uganda: opportunities for improving access to maternal health services. Global health action. 2017;10(sup4):1347363.

29. Kotoh AM, Aryeetey GC, Van der Geest S. Factors that influence enrolment and retention in Ghana'National Health Insurance Scheme. International journal of health policy and management. 2018;7(5):443.

30. Odeyemi IA. Community-based health insurance programmes and the national health insurance scheme of Nigeria: challenges to uptake and integration. International journal for equity in health. 2014;13(1):20.

31. Mebratie AD, Sparrow R, Yilma Z, Alemu G, Bedi AS. Enrollment in Ethiopia's community-based health insurance scheme. World Development. 2015;74:58-76.

32. Jofre-Bonet M, Kamara J. Willingness to pay for health insurance in the informal sector of Sierra Leone. PloS one. 2018;13(5):e0189915.

33. Khatiwada B, Ghimire S, N S. Willingness to pay for health insurance in mangalbare village development committee of illam district. MOJ Public Health. 2017;5(2):43-6.

34. Mathiyazaghan K. Willingness to pay for rural health insurance through community participation in India. The International journal of health planning and management. 1998;13(1):47-67.

35. Donfouet HPP, Mahieu P-A, Malin E. Using respondents' uncertainty scores to mitigate hypothetical bias in community-based health insurance studies. The European Journal of Health Economics. 2013;14(2):277-85.

36. Oriakhi H, Onemolease E. Determinants of rural household's willingness to participate in community based health insurance scheme in Edo State, Nigeria. Studies on ethno-medicine. 2012;6(2):95-102.

37. Adebayo EF, Uthman OA, Wiysonge CS, Stern EA, Lamont KT, Ataguba JE. A systematic review of factors that affect uptake of community-based health insurance in low-income and middle-income countries. BMC health services research. 2015;15(1):543.

38. Dror DM, Hossain SS, Majumdar A, Koehlmoos TLP, John D, Panda PK. What factors affect voluntary uptake of community-based health insurance schemes in low-and middle-income countries? A systematic review and meta-analysis. PLoS One. 2016;11(8):e0160479.

Manuscript

Click here to access/download;Manuscript;Clean Manuscript 20-12-24.docx

Willingness and Perceived ability to pay for Uganda's Proposed National Health Insurance Scheme among Informal Sector workers in Iganga and Mayuge districts, Uganda: A Contingent Valuation Method.

Noel Namuhani[1*], Angela Kisakye[1, 2] and Suzanne N Kiwanuka[1]

Institutional affiliations

[1]Makerere University School of public health, Department of Health policy planning and management, Uganda

[2]African Field Epidemiology Network, Lugogo House, Plot 42, Lugogo By-Pass

*Corresponding Author

Email: nnamuhani@musph.ac.ug

Abstract

Background: Access to healthcare remains a challenge, particularly among informal sector workers in most low-income countries, due to high out-of-pocket expenditures (OOPE). In Uganda, over 28% of healthcare costs are paid out of pocket. In response, Uganda has proposed a national health insurance scheme (NHI). However, the willingness and ability to pay for the proposed NHI scheme within the informal sector have not yet been explored in Uganda. This study assessed the willingness and perceived ability to pay for the proposed NHI scheme, along with its determinants, among informal sector workers in Iganga and Mayuge districts, Uganda.

Methodology: A cross-sectional study was conducted in Iganga and Mayuge districts between 10/04/2019 and 23/05/2019. A contingent valuation method using the bidding game technique was used to elicit the willingness to pay (WTP). A total of 853 informal sector workers, including farmers, commercial motorists, fishermen, and traders, were randomly sampled. Seven focus group discussions (FGD) were also conducted. Logistic regression was done to identify the determinants of willingness to pay for the proposed NHI scheme. Qualitative data was analyzed thematically.

Results: The majority 695/853, (81.5%) of the respondents were willing to pay for NHI; the median WTP was UGX 25,000 (USD 6.8) annually; and 633/853, (74.2%) of the respondents believed that they were able to pay for the health insurance. Willingness to Pay was significantly associated with being a fisher folk (AOR: 1.70 95%CI: 1.04-2.79, P = 0.035), being in the fourth wealth quintile (AOR: 2.98, 95% CI: 1.56–5.65), not hearing about health insurance (AOR: 0.50 95%CI: 0.23-0.86, P = 0.032), and not having saving group membership (AOR: 0.51, 95%CI: 0.34-

0.76, P<0.001). Most of the FGD participants were willing to pay for the proposed scheme; however, some of the participants doubted their ability to pay for the scheme given their high poverty levels and their unstable income.

Conclusion: The willingness to pay for health insurance in the informal sector is high. Therefore, it is viable for the government to extend NHI to the informal sector. However, awareness building and due consideration of high poverty levels in setting appropriate premiums should be a priority.

Key words: National Health insurance scheme, Willingness to pay, Ability to pay, informal sector, Contingent Valuation method.

Author Summary

Many Ugandans, especially those in the informal sector, struggle to access healthcare due to high out-of-pocket healthcare costs. To address this, Uganda plans to establish a national health insurance scheme. However, the extent to which informal sector workers — who constitute the largest portion of the labor force — are willing and able to pay remains unclear. This paper, therefore, seeks to explore the willingness and ability of informal sector workers to pay for the proposed national health insurance scheme.

To answer this objective, we interviewed 853 workers engaged in farming, fishing, trade, and commercial cycling in Iganga and Mayuge districts. We found that most respondents (82%) were willing to pay for the proposed health insurance, with an average annual amount of UGX 25,000

(about USD 6.80). Over 74% believed they could afford to pay for the proposed scheme, while others expressed concerns about their ability to pay due to poverty and unstable incomes.

Our study findings suggest that extending the health insurance scheme to informal sector workers is feasible. However, the government needs to consider affordability when setting premiums, given the high levels of poverty.

Introduction

Globally, there is a growing commitment to achieving Universal Health Coverage (UHC), which is an important element for achieving the Sustainable Development Goals (SDGs) (1). UHC aims to address the poor access to quality and affordable health services, which many African countries are grappling with, mainly due to high out-of-pocket expenditures(OOPe) (4). In 2021, it was estimated that about 4.5 billion people (more than half) in the world were not fully covered by essential health services, while about 2 billion (a quarter) of the world's population were facing financial hardship due to out-of-pocket health spending (1).This put about 150 million people at risk of catastrophic healthcare costs annually, driving over 100 million people into poverty worldwide (4). According to the World Bank, direct modes of payment for health care services constitute 40% of health care expenditure in developing countries (5).

In Uganda, although the national health accounts reported a reduction in the out-of-pocket expenditure on health as a share of current health expenditure (CHE) from 38.6% in the financial year 2018/2019 to 27.4% in the financial year 2020/2021, the OOPE remains significantly high. This is compounded by the complex health financing landscape, where the general government health expenditure per capita allocation is very low estimated at 11.6 $, heavy reliance on donor

funding accounting for 50.3% of the total health expenditure which has significantly reduced over the years. (6). Uganda abolished user fees in public health facilities, however, accessing healthcare is still associated with several expenses, including transport, drugs, diagnostics, and consultancy fees, among other expenses related to personnel and capital costs (7).

Globally, more than 60% of the employment occurs in the informal sector and in Uganda, over 80% of the labor force belongs to the informal sector (8). These include peasant farmers, commercial cyclists, fishermen, and those employed in unregistered or small-scale enterprises. It is estimated that 59% of the people in the informal sector in Eastern Uganda cannot afford to access quality health care due to high poverty levels and OOP for health care (9).

Globally, the implementation of health insurance schemes is one of the major responses to addressing out-of-pocket payments for health care (4, 10) in low and middle-income countries. Health insurance has the ability to reduce financial barriers to health care access and protect individuals and families against the risk of unpredictable health care expenditures, hence aiding the attainment of UHC (11). This has improved access to healthcare, especially in developed countries.

High coverage and enrollment in the health insurance scheme are critical for the success and sustainability of the scheme, given that premiums constitute the proposed main source of funding for the scheme. However, enrolment in health insurance schemes remains low, ranging from 2% to 40% in most African countries (12-14). The poor enrolment rates in the different countries have been attributed to a number of factors, including low incomes, large household sizes, long distances to health facilities, limited awareness, poor quality of health care services,

inappropriate benefit packages, a lack of trust in the systems, and high illiteracy levels, especially in the informal sector (15-17). The informal sector workers are sometimes of low socioeconomic status compared to the formal sector workers, hence having a lower ability to pay for health insurance (17). Furthermore, the informal sector is not well organized; they have unpredictable incomes, making it difficult to enroll and register them into the NHI scheme and collect regular contributions from them (14, 18).

Uganda is in the process of developing a NHI scheme to address the high OOP and access to services. However, given the high poverty levels among Ugandans and more so in the informal sector, the policy question of how much money people are willing to spend and their ability to pay remains unanswered. Eastern Uganda is the poorest region in Uganda, with 35.7% of most households living below the poverty line (8). The willingness to pay for the proposed NHI scheme, especially within the informal sector, which makes up 80% of Uganda's population, is critical to the achievement of equity in access to care and the core risk pooling function of a health insurance scheme. This would translate into high enrollment rates, which foster the financial sustainability of the schemes (11).

The National Health Insurance Bill, 2019, proposes that the scheme will be mandatory for all Ugandans, covering both formal and informal sector workers. Each person will be required to pay a premium, the amount of which will be determined by the board, to access the scheme. The main sources of funding for the proposed scheme are premium contributions and government contributions for the indigent. Services will be provided in both public and accredited private health facilities. The bill was passed by Parliament in 2021 but was not assented to by the

president due to disagreements among stakeholders who were not consulted. As of 2024, the bill is being discussed by the Ministry of Health and other stakeholders before being re-tabled in the Parliament of Uganda.

There is a paucity of information on the willingness and ability to pay for the proposed National Health Insurance (NHI) scheme, particularly within the informal sector. Understanding the level of willingness to enroll and the factors that influence the willingness to pay is crucial in designing strategies to improve enrollment rates in the scheme. Therefore, this study aimed to document the willingness to pay and the perceived ability to pay for the proposed NHI, along with the factors influencing willingness to pay among informal sector workers. The study's findings will be used by the Ministry of Health (MOH) to determine affordable premiums and design strategies to ensure maximum participation, especially among informal sector workers. The findings will also inform the National Health Insurance Scheme bill currently under discussion.

Materials and Methods

Study design: This was a cross-sectional study design that involved mixed methods of data collection. The quantitative and qualitative methods were conducted concurrently. The study was conducted from 10/04/2019 to 23/05/2019. This study adopted a contingent valuation method (CVM) using the bidding game technique to directly elicit or measure willingness to pay for a hypothetical (not yet on the market) health insurance package (19). This approach has been used because it has been proven to be an effective way of assessing willingness to pay with minimal bias for goods that are not presently available on the market. The CVM creates a hypothetical marketplace in which no actual transactions are made; hence, this approach has

been successfully used for commodities that are not exchanged in regular markets. This approach makes the assumption that people have had no previous experience buying the health service that is going to be put on the market and instead asks people their willingness to pay on the basis of their expectations and the benefits attached to the service (20). In the absence of markets for public goods, this method presents consumers with hypothetical markets in which they have the (hypothetical) opportunity to buy the good, health insurance in this case. Since the elicited WTP values are contingent upon the particular hypothetical market described to the respondent, this approach is called the contingent valuation method (21).

Study setting: The study was conducted in the Iganga and Mayuge districts of eastern Uganda. Iganga and Mayuge districts were selected purposively because they are the most poverty-hit districts in the country and 80% of the population lives in rural areas. They also have 80–85% of the population belonging to the informal sector (8). Iganga District is bordered by Kaliro District to the north, Bugweri District to the east, Mayuge District to the south, Jinja District to the southwest, and Luuka District to the west. With a total population of 504,197 people, 9.9% of the households are 5 km or more from the nearest health facility, and 19.9% are near a public health facility (UBOS, 2016). It is composed of 3 counties, 8 sub counties, 66 parishes, and 395 villages. The major economic activity is farming. Mayuge district is located in south-eastern Uganda with a population of 473,2394 and a growth rate of 3.5%, and it has thirteen subcounties. The major economic activities are farming and fishing. Mayuge district is bordered by Lake Victoria to the south, as well as Jinja and Bugiri districts. It has a total of 33 landing sites where fishing activities take place. Uganda has a decentralized healthcare system, having both public and private health facilities. The MoH at the center is the regulator and it will regulate the operations of the NHIs.

At regional level, there are regional referral hospitals, and district level, where policies are implemented. At the district level exist district hospitals, health center IVs at county level, health center IIIs at the subcounty level, health center IIs at parish level and village health teams at village level.

Study Population: The study targeted the four major categories of informal sector workers: 1) farmers; 2) fishermen; 3) commercial cyclists; and 4) the business community (traders and market vendors) in the study area. The informal sector was chosen because they make up the largest (80%) portion of the population, without which the sustainability of the scheme will be questionable.

Sample Size: The sample size was calculated using the Kish Leslie formula (22), considering a 95% confidence interval, a 50% expected level of WTP, a 5% level of precision, a design effect of 2, and a 10% non-response rate. This gave a total sample size of 853 informal sector workers. Four focus group discussions were conducted with women (four FGDs), and three FGDs were conducted with men (in the informal sector). This number depends on the level of information saturation. This was determined by doing preliminary analysis while still in the field.

Sampling Procedure: Stratified random sampling was used in this study. For Iganga district, the informal sector workers were divided into three major categories (strata): farmers, commercial cyclists, traders/market vendors. In Mayuge district, the informal sector workers were divided into four categories: fishermen, farmers, commercial cyclists, and market vendors. A random sample from each stratum was taken in a number proportional to the stratum's size when compared to the population. In Iganga district, two sub counties were randomly selected, and

five villages from each subcounty were also randomly selected. A proportionate sample of 286 households from the 10 villages was selected, and the household heads were systematically selected for interviews. Seven *bodaboda* stages located along main roads and junctions were randomly selected, and 10 cyclists available at the time of the study were randomly selected to participate in the interviews. A total of 84 traders were selected systematically from the two main markets for interviews. In Mayuge district, a total of 10/33 landing sites were randomly selected, and 248 fishermen were selected from the 10 landing sites proportionate to size. Sixty-two traders were randomly selected from three main markets systematically, and 62 bodaboda cyclists were randomly selected from six stages located along main roads in Mayuge town (Table 1). Men and women from each of the categories were purposively selected for the focus group discussions. These were selected and identified with the help of local leaders based on community members who can express themselves, but also are residents and working in the informal sectors of interest.

Table 1: Sampling procedure per category of informal sector

**Iganga district (**Iganga district planning office, 2018)				
**Category**	**Farmers/peasants**	**Commercial cyclists**	**Traders/vendors**	**Fisherfolk**
Proportion in the district	0.65	0.16	0.19	
Sample size	(0.65*440) =286	(0.16*440) =70	(0.19*440) =84	-
Selection procedure	2 sub counties (SC) and 5 villages from each SC were randomly selected. Households proportionate to size selected.	7 *Boda boda* stages in the town were randomly selected, cyclists on stage randomly selected to be interviewed	Traders were systematically selected for the interviews two main markets	-
Mayuge district				

Category	Farmers/peasants	Commercial cyclists	Traders/vendors	Fisherfolk
Proportion in the district	0.1	0.15	0.15	0.6
Sample size	(0.1*413) =41	(0.15*413) =62	(0.15*413) =62	(0.6*413) =248
Selection procedure	One sub county and two villages were randomly selected, the number of households were determined proportionate to size.	6 *Boda boda* stages in town were randomly selected, later cyclists on stage at that time were randomly selected to be interviewed	Traders were systematically selected from 3 main markets	10/33 landing sites were randomly selected. Fishermen at each site were randomly selected, proportionate to size
**Total samples**	**327**	**132**	**146**	**248**

Data collection methods and tools

The willingness to pay was assessed using a contingent valuation method using the "bidding game" technique. The bidding game was used to determine the maximum premium that a respondent is willing to pay for the proposed NHI plan. A bidding game technique was chosen because of its ease of implementation and its minimal biases. In addition, many studies have found the bidding game to be very reliable (23, 24). The respondent was asked if he or she was willing to pay the starting bid. If the respondent agreed, the interviewer would raise the bid by 10% of the first bid and again ask if the respondent was willing to pay the new bid. The interviewer continued to present the different bids until the respondent expressed an unwillingness to pay. On the other hand, if the respondent expressed unwillingness to pay the starting bid, the interviewer lowered the bid by 10% and repeated the query. This was continued until a bid was reached where the respondent was willing to pay. The starting bid was 20,000

UGX (5.4 USD), since this was the average premium for beneficiaries in the existing community-based health insurance schemes in Luweero, Western Uganda, and Jinja. The proportion of those willing to pay, the average, and the median amount of money people were willing to pay were computed. The perceived ability to pay was measured by asking respondents whether they were able to pay premiums for health insurance per year and how much they could afford without foregoing other basic needs.

A semi-structured questionnaire was used to collect quantitative data such as sociodemographics, WTP, perceived ability to pay, and associated factors from informal sector workers. The data collection tools were translated to the local language and then back translated to English to check whether the translated questions still held informational validity. The principal investigator recruited and trained research assistants so that they were familiar with the statement of the problem, objectives of the study, sampling procedure, data collection tools, and plan for data collection. Research assistants were oriented to the bidding game technique for assessing WTP. They were also taught the meaning of health insurance and how it intends to be operationalized in the country. Research assistants were also trained on the basic interview techniques, such as asking questions in a neutral manner, not showing by words or actions what answers were being expected of the study participants, and how to record answers, especially from open-ended questions, without interpreting them. The data collection tools were pre-tested in the neighboring district, Bugweri district. This was done to test the validity and ease of application of the tools. Filled electronic forms were checked at the point of data collection for completeness, and those found incomplete were completed before the respondent was

discharged. The data were cleaned and edited by the research assistants before they submitted the complete forms to the server.

Focus group discussion (FGD) guides were also used to collect data from informal sector workers to understand the determinants of willingness to pay and their perceived ability to pay for the NHI. The FGDs were audio-taped. The FGD was composed of 6–8 participants. The discussion had a moderator and a note-taker, and the discussions lasted for an average of one hour. These were conducted in the local language, and these were audio recorded.

Data management and analysis: The quantitative data were downloaded from the server and imported into STATA 14 for cleaning and analysis. Descriptive statistics, including mean, median, standard deviations, frequencies, and percentages, were obtained. The information was presented in frequency distribution tables. Principal component analysis was done to generate wealth quintiles using the demographic health survey categorization. The wealth quintiles were generated based on nine household items, which included possession of a car, motorcycle, bicycle, radio, television, mobile phone, piece of land, owning a house, and having animals. Inferential statistics were obtained using logistic regression. This was done in two stages: 1) bivariate analysis was done to determine the potential variables associated with willingness to pay for health insurance. Crude odds ratios at a 95% confidence interval were obtained to measure the potential association. 2) A multivariate analysis was done to determine the actual factors associated with WTP. All variables that were statistically significant at bivariate analysis and the important factors known in the literature to be statistically significant, such as socio-economic status, occupation, and potential confounders like residence, were included in the

model. Variables were tested for multicollinearity before being included in the model. The model was built step-by-step while testing it using lfit until the best model was obtained. Variables that remained statistically significant at 95% confidence were considered the factors associated with WTP for health insurance. Adjusted odds ratios were obtained as measures of association.

Qualitative data was analyzed manually using a thematic-inductive approach. The recordings were transcribed verbatim and translated from the local language (Lusoga) to English. Transcripts were read by more than two independent analysts who identified codes, sub-themes, and then organized them into themes. The information was triangulated with the quantitative data findings to gain a deeper understanding.

Ethics statement

The study was approved by the Makerere University, School of Public Health Higher Degrees, Research and Ethics Committee (HDREC). At the district level, permission to conduct the study was obtained from the local leaders and the District Health Office. Permission was also sought from subcounty chiefs and local council one chairpersons. The informed written consent of each individual participant was obtained at the start of the study. Confidentiality and anonymity were maintained by the use of codes.

Results

Background Characteristics of the Respondents

Of the 853 respondents who participated in the study, 327 (38.3%) were peasant farmers, 248929.1%) were fishermen, 146 (17.1%) were business people, and 132 (15.5%) were

commercial cyclists. The mean age of the respondents was 37.0 years (SD ± 11.3). The majority 667/853 (78.2%) of the respondents were males, 783/853 (91.8%) of the household heads were males, and 618/853 (72.4%) resided in rural areas. The average household size was 5 persons. The median monthly income was UGX 150,000 (USD 40.5). Only 158/853 (18.5%) were in the wealthiest quintile, while 349/853 (41.0%) of the households were in the second and poorest wealth quintiles (Table 2).

Table 2: Background Characteristics of the Respondents

Variable	Frequency (N=853)	Percentage (%)
**Sex of respondent**		
Male	667	78.2
Female	186	21.8
**Sex of the household head**		
Male	783	91.8
Female	70	8.2
**Residence**		
Rural	618	72.4
Urban	235	27.6
**Marital status**		
Married	694	81.4
Single	85	10.0
Separated	48	5.6
Widowed	26	3.0
**Age category**		
18-25	124	14.5
26-35	303	35.5
36-45	239	28.0
46-55	126	14.8
56 above	61	7.2
**Education level**		
None	51	6.0
Primary	445	52.2
Secondary	280	32.8
Tertiary	77	9.0
**Religion**		
Muslim	304	35.6

Protestant	276	32.4
Catholic	194	22.7
Born again	53	6.2
SDA	26	3.1
**Occupation**		
Farmer	327	38.3
Fishermen	248	29.1
Business	146	17.1
Bodaboda	132	15.5
**Wealth quintiles**		
Poorest (<-1.39)	172	20.2
Poor (-1.40 - -0.50)	177	20.8
Moderate (-0.51 -0.29)	163	19.1
Wealthy (0.30 -1.23)	183	21.5
Wealthiest(1.24-4.14)	158	18.5

Willingness to Pay for the Proposed NHI

The majority 695/853 (81.5%) of the respondents were willing to pay for the proposed NHI. Of those willing to pay, the median willingness to pay was UGX 25,000 (USD 6.8) while the mean WTP was UGX 28,950 (USD 7.8) per person, inclusive of 4 dependents per year. Only 3.8% protested contributing to the scheme, and 126/853 (14.7%) gave WTP zero because they believed they were too poor to pay for national health insurance (Table 3).

Qualitatively, most of the FGD participants were also willing to make contributions for health insurance. However, others resented making contributions, as noted below:

> *"Sincerely, the government is meant to provide all the medical services. So, there is no way that you can lie to us and say that the government can't provide those services..."* **(FGD male-fishermen-Mayuge)**

A number of FGD participants were not willing to pay because of poverty, while others criticized the government's misuse of taxes collected from the tax payer on salaries for officials rather than on health services, as noted in the quotes below.

> *"The fact is that poverty is the biggest hindrance. Because at times you can fall sick, yet you don't have money, and you resort to borrowing from colleagues who may not even be able to help you out because they also don't have. So generally, I disagree with paying for health insurance."* **(FGD-Business Women, Mayuge District)**

> *"I am not willing to pay because we are always charged a lot of tax to register our businesses. That is all tax. Where does that tax go? Let them reduce the salary of Members of Parliament so that part of it is utilized to buy medicine. Members of Parliament have no importance in our area."* (**FFGD-Male-fishermen-Mayuge district)**

Willingness to Pay with Copayments:

A copayment is a cost-sharing arrangement where one covers a portion of the costs out of pocket each time one visits the health facility. Less than half, 309/695 (44.5%) of the respondents, were willing to co-pay. The mean co-pay amount was UGX 2,650 (USD 0.7), with a median of UGX 2000 (USD 0.5), each time someone accessed services. The average maximum amount of money that respondents were willing to pay with the copayment was UGX 23,000 (USD.6.2) and the median was UGX 20,000 (USD.5.4) (Table 3).

Table 3: Willingness to Pay for the Proposed NHI annually

WTP	Frequency (%)	Mean	Median
Protests (WTP=0)	32 (3.8)	0	0

WTP=0	126 (14.7)	0	0
WTP>0	695(81.5)	**28,950**	**25,000**
Total	853 (100.0)	23,600	20,000
**Copayment scenario**			
Amount willing to copay	309/695(44.5)	**2,650**	**2,000**
WTP with co payment	309/695(44.5)	23,000	20,000

Notes; *Protests-those protested attaching valuing to HI (WTP=0 but it has no economic sense).*

WTP=0 means willingness to pay is actually zero. WTP>0 that it is positive -WTP is above zero.

Qualitatively, most of the FGD participants were not in support of copayments, as expressed by one of the FGD participants.

> *"I also do not support the idea that we pay money each time we visit the health facility, especially when we are paying every year. This will make people unable to access the services, and hence the problem of access to services will not have been solved. People are already poor, and that will mean people will continue to sell their property to go to health facilities."* (FGD Male Farmer)

Preferred Mode of Payment

Most (54.1%) respondents preferred to pay annually, while 21.0% preferred to pay per harvesting season. Only 13.1% wanted to pay according to their monthly income, with the majority (63.1%) preferring to pay 1 percent of their monthly income, followed by those who were willing to pay 5%.

Perceived Ability to Pay for the Health Insurance Scheme

More than half 633/853 (74.2%) of the respondents believed that they were able to pay for health insurance, with a mean of UGX 23,000 (USD.6.2) and median of UGX 20,000 (USD.5.4) per person per year.

Regarding the perceived ability to pay, most of the FGD participants noted that most of the people are poor and most likely may not be able to make periodic subscriptions, especially if the money is raised more than what people earn. This was attested to, as indicated below:

> *"Sincerely speaking, some people cannot afford to pay money; for instance, you may find a very old person who is no longer earning anything, yet they also need health care."* **(FGD-Boda-boda-Iganga district)**

Another FGD participant, who was a business lady, noted that

> *"I support the idea. But not very much because of the issue of affordability. Not everyone can afford to pay, and note that we earn differently. Because some of us play the role of mother and father in the family, there are a lot of requirements to provide."* **(FGD Business Women, Iganga)**

Furthermore, most of the participants noted that setting premiums should be based on people's income and ability to pay, as noted below:

> *"Since everyone earns differently, the charges should depend on how much someone earns. I could be in a position to afford UGX 50,000, but yet my colleague cannot at all since people in the village are used to getting free medicine. When some people are asked to pay just 500shs for drugs, they will consider it unworthy because they know that drugs/medication are freely given. So, if possible, we charge according to people's income."* **(FGD: business women, Iganga district)**.

Determinants for level of Willingness to Pay for Health Insurance Scheme

At bi-variable analysis, wealth quintiles, having savings group membership, having a family member with chronic illness, use of traditional medicine, and hearing about health insurance were statistically significant for willingness to pay for health insurance. The odds of being willing to pay when in the fourth wealth quintile were 2.88, compared to the odds of being willing to pay among those in the poorest category (COR: 2.88, 95% CI: 1.60–5.18, P<0.001). Not being in the saving group reduced the chances of being willing to pay for health insurance by 42% compared to those who had saving group memberships (COR: 0.58, 95% CI: 0.33-0.69, P<0.001) (Table 4).

After adjusting for any confounding factors, occupation, wealth, saving group membership, use of traditional medicine, and having heard about health insurance were significantly associated with the level of willingness to pay for the proposed health insurance scheme.

The odds of fishermen's willingness to pay for health insurance were 1.70 compared to those of farmers after adjusting for other factors (AOR: 1.70, 95% CI: 1.04–2.79, P = 0.035). Respondents in the fourth wealth quintile were 2.98 times more likely to pay for health insurance compared to those in the poorest wealth quintile (AOR: 2.98, 95% CI: 1.56–5.65). Respondents who were not in any saving group were 0.51 times less likely to pay for the proposed health insurance compared to those with saving group membership (AOR: 0.51, 95% CI: 0.34-0.76, P<0.001), and individuals who had never heard about HI were 50% less willing to pay for the proposed National Health Insurance compared to those who had ever heard about health insurance (AOR: 0.50, 95% CI: 0.23-0.86, P = 0.032) (Table 4).

Table 1: Multi variable analysis of factors associated with WTP for NHI scheme.

Variable	Level of Willingness to pay		COR (95%CI)	AOR (95%CI)
	No (n=158)	Yes (n=695)		
**Sex of respondent**				
Male	127(80.4)	540(77.7)	1.0	
Female	31(19.6)	155(22.3)	1.18(0.76-1.81)	1.30(0.80-2.11)
**Residence**				
Rural	110(69.6)	508(73.1)	1.0	
Urban	48(30.4)	187(26.9)	0.84(0.58-1.23)	1.09(0.52-2.26)
**Occupation**				
Farmer	60(38.0)	267(38.4)	1.0	
Business	32(20.2)	114(15.4)	0.80(0.49-1.30)	0.59(0.26-1.35)
Boda boda	25(15.8)	107(15.4)	0.96(0.57-1.61)	1.04(0.50-2.18)
Fishermen	41(26.0)	207(29.8)	1.13(0.73-1.76)	1.70(1.04-2.79) *
**Wealth quintiles**				
Poorest	43(27.2)	129(18.6)	1.0	
Second poorest	32(20.3)	145(20.9)	1.51(0.90-2.53)	1.83(1.05-3.19) *
Middle	35(22.1)	128(18.4)	1.22(0.73-2.03)	1.23(0.70-2.14)
Fourth	19(12.0)	164(23.6)	2.88(1.60-5.18)	2.98(1.58-5.65) *
Wealthiest	29(18.4)	129(18.6)	1.48(0.87-2.52)	1.53(0.81-2.88)
**In saving group**				
Yes	49(31.0)	337(48.5)	1.0	
No	109(69.0)	358(51.5)	0.48(0.33-0.69)	0.51(0.34-0.76) **
**Family member with chronic illness**				
Yes	36(22.7)	241(34.7)	1.0	
No	122(77.2)	576(67.5)	0.56(0.37-0.83)	0.73(0.46-1.14)
**Ability to meet health care**				
Difficult	112(70.9)	571(82.2)	1.0	
Easy	46(29.1)	124(17.8)	0.53(0.36-0.78)	0.68(0.43-1.05)

**Use Traditional Medicine**				
No	59(38.8)	345(51.4)	1.0	
Yes	93(61.2)	326(48.6)	0.60(0.42-0.86)	0.71(0.48-0.92) *
**Ever heard about NHI**				
**Yes**	9(5.7)	83(11.9)	1.0	
**No**	149(94.3)	612(88.1)	0.45(0.22-0.91)	0.50(0.23-0.86) *

Note: ** $p<0.001$, $*P<0.05$

Discussion

This study assessed the willingness and perceived ability to pay for the proposed National Health Insurance (NHI) scheme and its determinants among informal sector workers in Iganga and Mayuge districts, Uganda. The study found that the majority of informal sector workers were both willing and believed they were able to pay for the proposed NHI. Willingness to pay was significantly associated with occupation, wealth, awareness, and membership in saving groups.

The level of WTP for the proposed NHI was high, with a median willingness to pay of UGX 25,000 (USD 6.8) and a mean WTP of UGX 28,950 (USD 7.8) per person, inclusive of 4 dependents per year. This level of willingness to pay was higher than the reported WTP in Nepal (71%) and in South Sudan (68%) among the informal sector (14, 25). However, the maximum amount of money people were willing to pay was lower than the 5.5 USD per person per month in Iran, 6.6 USD per person per month in Namibia, and 3 USD in Kenya per month (19, 25, 26). The differences in WTP across the different countries could be due to the differences in the socio-economic status of the different populations in the different countries. The high level of willingness to pay implies a high level of support for the proposed National Health Insurance scheme among the informal

sector workers, contribute to the proposed NHIs and therefore, the need for government accelerate efforts of implementing the scheme. However, the suggested premium being higher than the stated WTP may hinder many from participating in the scheme. But this also brings up the policy question of whether the WTP can sustain the scheme since premiums are the major source of funding for the scheme. Thus, there is a need for careful revision of the premiums before Parliament passes the NHI bill. The premiums should be based on the ability to pay.

The ability to pay is another important consideration when planning to implement health insurance, especially among the informal sector, whose incomes are unpredictable. A high number of participants believed that they could afford to make periodic contributions to the health insurance scheme in this study. This finding further highlights the potential for the NHI to succeed if the informal sector is included. However, the ability to pay needs to be considered when setting premiums, given the fact that less than 20% of the studied population were in the wealthiest category.

Although more than half of the respondents earned more than 100,000 UGX (27 USD) a month, there are substantial sections of the informal sector that will struggle to pay for health insurance, and therefore, these will require significant government subsidies to be included. This further highlights the need to consider the idea of making contributions as a proportion of people's earnings and wealth status because it would make people pay according to their ability and is more equitable than just having a flat figure for all people. Moreover, by virtue of the unpredictable nature of their income, informal sector workers expressed the desire to have premiums synchronized to earning cycles such as harvest seasons. This implies that the NHI will

have to explore flexible premium schedules for informal sector workers in order to enhance their uptake of the scheme. In Ethiopia, making premium systems more flexible eased payments (27).

In this study, having a savings group membership increased the likelihood of willingness to pay for the proposed national health insurance scheme. In Asian countries, saving groups were reported to be key in initiating community-based health insurance schemes (28). Savings groups provide members with a secure place to save money, generate a pool fund, and have the opportunity to borrow in small amounts. They also provide affordable basic insurance services and enable the community to meet the premiums(28). Local savings groups have become one of the most famous financial support systems in rural Uganda over the last decade (29). Savings and burial groups present an opportunity to leverage in enhancing access to health care in rural communities. This implies that as countries plan to initiate NHI, saving groups can become a great resource to tap into to ensure community participation and payment of the premiums for the scheme.

Participants belonging to the highest wealth category were more likely to pay for the proposed national health insurance scheme. This is in agreement with studies conducted in Nigeria, Ethiopia, and Ghana, which have reported that socio-economic factors such as poverty, income, or wealth status have a bearing on the ease or difficulty of enrolling in a given scheme, with high premiums deterring enrollment in schemes (30-32). Similar studies conducted in Sierra Leone and Iran showed that the WTP for the HI scheme depended on the monthly income of the respondent (26, 33) . In Nepal, belonging to a lower wealth quartile was associated with a lower willingness to pay for National Health Insurance Scheme (34). This therefore underscores the

need for income generating projects to be implemented within the communities to aid them make contributions for health insurance.

The use of traditional medicine was negatively associated with participation in the health insurance scheme. This is because members who use traditional medicine see no need to utilize modern health care services, making them less likely to pay for health insurance. Studies have shown that the presence and use of alternative medicine and other forms of healthcare, such as herbalists, negatively affects participation in a health insurance scheme since insurance requires regular use of modern medicine. Studies conducted in Cameroon, Burkina Faso, Nigeria, and India have reported that those who use modern medicine are more willing to pay for health insurance than those who use traditional medicine (35-37). In Uganda's pluralistic health system, the ministry of health and other mandated regulatory bodies need to institute measures that not only regulate the alternative sector but ultimately attract people to use modern medicine as opposed to traditional medicine.

It is not surprising that those who had never heard about health insurance had 50% less WTP compared to those who had prior information about health insurance. Indeed, the level of knowledge on how health insurance operates was very low, and statistical tests of significance could not be used in this study. However, one systematic review and meta-analysis of factors influencing WTP for voluntary contributary health insurance schemes in LMICs has revealed that knowledge and understanding of the functioning of the scheme positively influence the willingness to pay for and participate in the scheme (38, 39). As the Ugandan government moves to implement health insurance, information, education, and communication will be paramount to the success of the proposed scheme.

Study Limitations: As with most contingent valuation studies, the elicitation technique is always subject to bias, and the assignment of the first bid or amount is also prone to bias. However, this was triangulated with an open-ended question and qualitative data. The low level of knowledge about health insurance among respondents was another limitation in attaching value to the proposed scheme. This was addressed by educating the respondents about health insurance before eliciting their willingness to pay. Despite these limitations, the study provides valuable insights into the willingness to pay for the proposed scheme among informal sector workers, who are often excluded from social protection programs.

Conclusions

This study indicated a high level of willingness to pay (WTP), with the majority believing they could afford to pay for the health insurance scheme. This suggests that the informal sector is willing to contribute to their health, making it feasible for the government to introduce a contributory scheme, with the public bearing some costs towards their healthcare. However, there is a need to revise the proposed premiums, considering that a large portion of the informal sector may struggle to meet them, which could affect coverage.

Additionally, studies on the actual ability to pay should be conducted, as this study is based on the perceived ability to pay for health insurance schemes. To support the ability to pay, local solidarity groups should be built, and local initiatives to assist poorer members should be strengthened.

The determinants of willingness to pay included occupation, wealth status, use of traditional medicine, and prior knowledge of health insurance. This suggests that building trust among

households and improving awareness are critical to the success of the scheme. There is a need for the Ministry of Health (MOH) and district health departments to design an information and education campaign to sensitize the general public about how health insurance works, encouraging buy-in and fostering trust, especially among the informal sector.

Declarations

Consent for publication

Not Applicable

Availability of data and materials

The dataset analyzed for this study has been uploaded as a supplementary file.

Competing interests

The authors declare that they have no competing interests

Funding

The author(s) received no specific funding for this work

Authors' contributions

NN, SNK, AK conceived the study, NN collected, analyzed and wrote the first draft of the manuscript. SNK and AK provided reviews on all drafts of manuscript. All the authors read and approved the final manuscript.

Acknowledgements

We wish to thank the District Health Officers of Mayuge and Iganga districts for their cooperation, mobilization and ensuring that data collection went on smoothly. We also extend our sincere appreciation to the research assistants and all respondents in Iganga and Mayuge for sparing their time to contribute to this piece of work.

References

1. WHO. Universal health coverage (UHC): World Health Organization 2023 [Available from: https://www.who.int/news-room/fact-sheets/detail/universal-health-coverage-(uhc).
2. Sarker AR, Sultana M, Mahumud RA, Ahmed S, Islam Z, Morton A, et al. Determinants of enrollment of informal sector workers in cooperative based health scheme in Bangladesh. PloS one. 2017;12(7):e0181706.
3. Kiwanuka S, Ekirapa E, Peterson S, Okui O, Rahman MH, Peters D, et al. Access to and utilisation of health services for the poor in Uganda: a systematic review of available evidence. Transactions of the Royal Society of Tropical Medicine and Hygiene. 2008;102(11):1067-74.
4. WHO. Health Systems Financing: The Path to Universal Coverage. Geneva: World Health Organization 2010.
5. World Bank. Financing health in low-income countries. Washington DC: World Bank Group; 2010.
6. MoH. National Health Accounts 2018/19-2020/21 Report. 2021.
7. Tashobya CK, Ssengooba F, Cruz VO, Yates R, Murindwa G, McPake B. Health systems reforms in Uganda: processes and outputs. Christine Kirunga Tashobya FS and, Valeria Oliveira Cruz, editors. 2006.
8. UBOS. The National Population and Housing Census 2014 – Main Report. Kampala, Uganda: Uganda Bureau of Statistics; 2016.
9. Twaweza. Health Check: Ugandans' experiences and opinions on health services Uganda: Twaweza; 2019 [Available from: https://www.twaweza.org/go/szw-ug-health-check.
10. Gottret P, Schieber G. Health financing revisited: a practitioner's guide: The World Bank; 2006.
11. Xu K, Evans DB, Carrin G, Aguilar-Rivera AM, Musgrove P, Evans T. Protecting households from catastrophic health spending. Health affairs. 2007;26(4):972-83.
12. De Allegri M, Sanon M, Bridges J, Sauerborn R. Understanding consumers' preferences and decision to enrol in community-based health insurance in rural West Africa. Health policy. 2006;76(1):58-71.
13. Kyomugisha E, Buregyeya E, Ekirapa E, Mugisha J, Bazeyo W. Strategies for sustainability and equity of prepayment health schemes in Uganda. African health sciences. 2009;9(2).
14. Basaza R, Alier PK, Kirabira P, Ogubi D, Lako RLL. Willingness to pay for National Health Insurance Fund among public servants in Juba City, South Sudan: a contingent evaluation. International journal for equity in health. 2017;16(1):158.
15. Nguyen H, Knowles J. Demand for voluntary health insurance in developing countries: the case of Vietnam's school-age children and adolescent student health insurance program. Social Science & Medicine. 2010;71(12):2074-82.

16. Acharya A, Vellakkal S, Taylor F, Masset E, Satija A, Burke M, et al. Impact of National Health Insurance for the Poor and the Informal sector in Low-and Middle-Income Countries. London: EPPI-Centre, Social Science Research Unit, Institute of Education, University of London; 2012.
17. Basaza R, Criel B, Van der Stuyft P. Community health insurance in Uganda: why does enrolment remain low? A view from beneath. Health Policy. 2008;87(2):172-84.
18. Jowett M, Kutzin J, Organization WH. Raising revenues for health in support of UHC: strategic issues for policy makers. World Health Organization; 2015.
19. Gustafsson-Wright E, Asfaw A, van der Gaag J. Willingness to pay for health insurance: An analysis of the potential market for new low-cost health insurance products in Namibia. Social science & medicine. 2009;69(9):1351-9.
20. Russell S, Fox-Rushby J, Arhina D. Willingness and ability to pay for health care: a selection of methods and issues. Health Policy and Planning. 1995;10(1):94-101.
21. Mitchell RC, Carson RT. Using surveys to value public goods: the contingent valuation method: Rff Press; 2013.
22. Kish L. Survey sampling. Inc., New York.: John Wiley and Sons; 1965.
23. Whittington D, Smith VK, Okorafor A, Okore A, Liu JL, McPhail A. Giving respondents time to think in contingent valuation studies: a developing country application. Journal of Environmental Economics and Management. 1992;22(3):205-25.
24. Dong H, Kouyate B, Cairns J, Sauerborn R. A comparison of the reliability of the take-it-or-leave-it and the bidding game approaches to estimating willingness-to-pay in a rural population in West Africa. Social Science & Medicine. 2003;56(10):2181-9.
25. Barasa EW, Mwaura N, Rogo K, Andrawes L. Extending voluntary health insurance to the informal sector: experiences and expectations of the informal sector in Kenya. Wellcome open research. 2017;2.
26. Nosratnejad S, Rashidian A, Mehrara M, Sari AA, Mahdavi G, Moeini M. Willingness to pay for social health Insurance in Iran. Global journal of health science. 2014;6(5):154.
27. Habte A, Tamene A, Ejajo T, Dessu S, Endale F, Gizachew A, et al. Towards universal health coverage: The level and determinants of enrollment in the Community-Based Health Insurance (CBHI) scheme in Ethiopia: A systematic review and meta-analysis. PloS one. 2022;17(8):e0272959.
28. Shaikh BT, Noorani Q, Abbas S. Community based saving groups: an innovative approach to overcome the financial and social barriers in health care seeking by the women in the rural remote communities of Pakistan. Archives of Public Health. 2017;75(1):57.
29. Mutebi A, Muhumuza Kananura R, Ekirapa-Kiracho E, Bua J, Namusoke Kiwanuka S, Nammazi G, et al. Characteristics of community savings groups in rural Eastern Uganda: opportunities for improving access to maternal health services. Global health action. 2017;10(sup4):1347363.
30. Kotoh AM, Aryeetey GC, Van der Geest S. Factors that influence enrolment and retention in Ghana'National Health Insurance Scheme. International journal of health policy and management. 2018;7(5):443.
31. Odeyemi IA. Community-based health insurance programmes and the national health insurance scheme of Nigeria: challenges to uptake and integration. International journal for equity in health. 2014;13(1):20.
32. Mebratie AD, Sparrow R, Yilma Z, Alemu G, Bedi AS. Enrollment in Ethiopia's community-based health insurance scheme. World Development. 2015;74:58-76.
33. Jofre-Bonet M, Kamara J. Willingness to pay for health insurance in the informal sector of Sierra Leone. PloS one. 2018;13(5):e0189915.
34. Khatiwada B, Ghimire S, N S. Willingness to pay for health insurance in mangalbare village development committee of illam district. MOJ Public Health. 2017;5(2):43-6.

35. Mathiyazaghan K. Willingness to pay for rural health insurance through community participation in India. The International journal of health planning and management. 1998;13(1):47-67.
36. Donfouet HPP, Mahieu P-A, Malin E. Using respondents' uncertainty scores to mitigate hypothetical bias in community-based health insurance studies. The European Journal of Health Economics. 2013;14(2):277-85.
37. Oriakhi H, Onemolease E. Determinants of rural household's willingness to participate in community based health insurance scheme in Edo State, Nigeria. Studies on ethno-medicine. 2012;6(2):95-102.
38. Adebayo EF, Uthman OA, Wiysonge CS, Stern EA, Lamont KT, Ataguba JE. A systematic review of factors that affect uptake of community-based health insurance in low-income and middle-income countries. BMC health services research. 2015;15(1):543.
39. Dror DM, Hossain SS, Majumdar A, Koehlmoos TLP, John D, Panda PK. What factors affect voluntary uptake of community-based health insurance schemes in low-and middle-income countries? A systematic review and meta-analysis. PLoS One. 2016;11(8):e0160479.

Manuscript

Click here to access/download;Manuscript;Clean Manuscript 4th May 2025 Final.docx

Willingness and perceived ability to pay for Uganda's proposed national health insurance scheme among informal sector workers in Iganga and Mayuge districts, Uganda: a contingent valuation method

Noel Namuhani[1*], Angela N Kisakye[1, 2] and Suzanne N Kiwanuka[1]

Institutional affiliations

[1]Makerere university school of public health, Department of health policy planning and management, Uganda

[2]African Field Epidemiology Network, Lugogo House, Plot 42, Lugogo By-Pass

*Corresponding author

Email: nnamuhani@musph.ac.ug

Abstract

Background: Access to healthcare remains a challenge, particularly among informal sector workers in most low-income countries, due to high out-of-pocket expenditures (OOPE). In Uganda, over 28% of healthcare costs are paid out of pocket. In response, Uganda has proposed a national health insurance scheme (NHIs). However, the willingness and ability to pay for the proposed NHIs within the informal sector have not yet been explored in Uganda. This study assessed the willingness and perceived ability to pay for the proposed NHIs, along with its determinants, among informal sector workers in Iganga and Mayuge districts, Uganda.

Methodology: A cross-sectional study was conducted in Iganga and Mayuge districts between 10/04/2019 and 23/05/2019. A contingent valuation method using the bidding game technique was used to elicit the willingness to pay (WTP). A total of 853 informal sector workers, including farmers, commercial motorists, fishermen, and traders, were randomly sampled. Seven focus group discussions (FGD) were also conducted. Logistic regression was done to identify the determinants of WTP for the proposed NHIs. Qualitative data was analyzed thematically.

Results: The majority 695/853, (81.5%) of the respondents were willing to pay for NHIs; the median WTP was UGX 25,000 (USD 6.8) annually; and 633/853, (74.2%) of the respondents believed that they were able to pay for the NHIs. WTP was significantly associated with being a fisher folk (AOR: 1.70 95%CI: 1.04-2.79, P = 0.035), being in the fourth wealth quintile (AOR: 2.98, 95% CI: 1.56–5.65), not hearing about health insurance (AOR: 0.50 95%CI: 0.23-0.86, P = 0.032), and not having saving group membership (AOR: 0.51, 95%CI: 0.34-0.76, P<0.001). Most of the

FGD participants were willing to pay for the proposed scheme; however, some of the participants doubted their ability to pay given their extreme poverty levels and their unstable income.

Conclusion: The WTP for the NHIs in the informal sector was high. Therefore, it is viable for the government to extend NHIs to the informal sector. However, building awareness and consideration of extreme poverty levels in setting appropriate premiums should be a priority.

Key words: National health insurance scheme, willingness to pay, ability to pay, informal sector, contingent valuation method.

Author summary

Many Ugandans, especially those in the informal sector, struggle to access healthcare due to high out-of-pocket healthcare costs. To address this, Uganda plans to establish a national health insurance scheme. However, the extent to which informal sector workers, who constitute the largest portion of the labor force, are willing and able to pay remains unclear. This paper, therefore, seeks to explore the willingness and ability of informal sector workers to pay for the proposed national health insurance scheme.

To answer this objective, we interviewed 853 workers engaged in farming, fishing, trade, and commercial cycling in Iganga and Mayuge districts. We found that most respondents (82%) were willing to pay for the proposed health insurance, with an average annual amount of UGX 25,000 (about USD 6.80). Over 74% believed they could afford to pay for the proposed scheme, while others expressed concerns about their ability to pay due to poverty and unstable incomes.

Our study findings suggest that extending the health insurance scheme to informal sector workers is feasible. However, the government needs to consider affordability when setting premiums, given the elevated levels of poverty.

Introduction

Globally, there is a growing commitment to achieving Universal Health Coverage (UHC), which is an important element for achieving the Sustainable Development Goals (SDGs) (1). UHC aims to address the poor access to quality and affordable health services, which many African countries are grappling with, mainly due to high out-of-pocket expenditures (OOPE) (2). In 2021, it was estimated that about 4.5 billion people (more than half) in the world were not fully covered by essential health services, while about 2 billion (a quarter) of the world's population were facing financial hardship due to out-of-pocket health spending (1).This put about 150 million people at risk of catastrophic healthcare costs annually, driving over 100 million people into poverty worldwide (2). According to the World Bank, direct modes of payment for health care services constitute 40% of health care expenditure in developing countries (3). However, developing countries suffer a higher burden of preventable disease, including the rising non communicable diseases (4). Thus, the need for health financing reforms to address this burden of disease, reduce OOPE and inequities.

In Uganda, although the national health accounts reported a reduction in the OOPE on health as a share of current health expenditure (CHE) from 38.6% in the financial year 2018/2019 to 27.4% in the financial year 2020/2021, the OOPE remains significantly high. This is compounded by the complex health financing landscape, where the general government health expenditure per

capita allocation is very low estimated at 11.6 $, heavy reliance on donor funding accounting for 50.3% of the total health expenditure which has significantly reduced over the years. (5). Uganda abolished user fees in public health facilities, however, accessing healthcare is still associated with several expenses, including transport, drugs, diagnostics, and consultancy fees, among other expenses related to personnel and capital costs (6).

Globally, more than 60% of the employment occurs in the informal sector and in Uganda, over 80% of the labor force belongs to the informal sector (7). These include peasant farmers, commercial cyclists, fishermen, and those employed in unregistered or small-scale enterprises. It is estimated that 59% of the people in the informal sector in Eastern Uganda cannot afford to access quality health care due to high poverty levels and OOPE for health care (8).

Globally, the implementation of health insurance schemes is one of the major responses to addressing OOPE on health care (2, 9) in low and middle-income countries. Health insurance has the ability to reduce financial barriers to health care access and protect individuals and families against the risk of unpredictable health care expenditures, hence aiding the attainment of UHC (10).

High coverage and enrollment in the proposed NHIs are critical for the success and sustainability of the scheme, given that premiums constitute the main proposed source of funding for the scheme. However, enrolment in health insurance schemes remains low, ranging from 2% to 40% in most African countries (11-13). The poor enrolment rates in the different countries have been attributed to a number of factors, including low incomes, large household sizes, long distances to health facilities, limited awareness, poor quality of health care services, inappropriate benefit

packages, a lack of trust in the systems, and high illiteracy levels, especially in the informal sector (14-16). The informal sector workers are sometimes of low socioeconomic status compared to the formal sector workers, hence having a lower ability to pay for health insurance (16). Furthermore, the informal sector is not well organized; they have unpredictable incomes, making it difficult to enroll and register them into the NHIs and collect regular contributions from them (13, 17).

Uganda is in the process of developing a NHIs to address the high OOPE and access to services. However, given the high poverty levels among Ugandans and more so in the informal sector, the policy question of WTP and their ability to pay remains unanswered. Eastern Uganda is the poorest region in Uganda, with 35.7% of most households living below the poverty line (7). The WTP for the proposed NHIs, especially within the informal sector, which makes up 80% of Uganda's population, is critical to the achievement of equity in access to care and the core risk pooling function of a health insurance scheme. This would translate into high enrollment rates, which foster the financial sustainability of the schemes (10).

The NHI Bill, 2019, proposes that the scheme will be mandatory for all Ugandans, covering both formal and informal sector workers. Each person will be required to pay a premium, the amount of which will be determined by the board, to access the scheme. The main sources of funding for the proposed scheme are premium contributions and government contributions for the indigent. Services will be provided in both public and accredited private health facilities. The bill was passed by Parliament in 2021 but was not assented to by the president due to disagreements

among stakeholders who were not consulted. As of 2025, the bill is being discussed by the MoH and other stakeholders before being re-tabled in the Parliament of Uganda.

There is a paucity of information on the willingness and ability to pay for the proposed NHIs scheme, particularly within the informal sector. Understanding the WTP and the associated factors is crucial in designing strategies to improve enrollment rates in the scheme. Therefore, this study aimed to document WTP and the perceived ability to pay for the proposed NHIs, along with the factors influencing WTP among informal sector workers. The study's findings will be used by the Ministry of Health (MOH) to determine affordable premiums and design strategies to ensure maximum participation, especially among informal sector workers. The findings will also inform the NHIs bill which is currently under discussion.

Materials and methods

Study design: This was a cross-sectional study design that involved mixed methods of data collection (convergent parrel mixed methods design). The quantitative and qualitative methods were conducted concurrently. The study was conducted from 10/04/2019 to 23/05/2019. This study adopted a contingent valuation method (CVM) using the bidding game technique to directly elicit or measure willingness to pay for a hypothetical (not yet on the market) health insurance package (18). This approach has been used because it has been proven to be an effective way of assessing WTP with minimal bias for goods that are not presently available on the market. CVM creates a hypothetical marketplace in which no actual transactions are made; hence, this approach has been successfully used for commodities that are not exchanged in regular markets. This approach makes the assumption that people have had no previous

experience buying the health service that is going to be put on the market and instead asks people their WTP based on their expectations and the benefits attached to the service (19). In the absence of markets for public goods, this method presents consumers with hypothetical markets in which they can buy a health insurance policy. Since the elicited WTP values are contingent upon the particular hypothetical market described to the respondent, this approach is called the contingent valuation method (20).

Study setting: The study was conducted in the Iganga and Mayuge districts of eastern Uganda. Iganga and Mayuge districts were selected purposively because they are the most poverty-hit districts in the country and 80% of the population lives in rural areas. They also have 80–85% of the population belonging to the informal sector (7). Iganga District is bordered by Kaliro District to the north, Bugweri District to the east, Mayuge District to the south, Jinja District to the southwest, and Luuka District to the west. With a total population of 504,197 people, 9.9% of the households are 5 km or more from the nearest health facility, and 19.9% are near a public health facility (UBOS, 2016). It is composed of 3 counties, 8 sub counties, 66 parishes, and 395 villages. The major economic activity is farming. Mayuge district is in south-eastern Uganda with a population of 473,2394 and a growth rate of 3.5%, and it has thirteen sub counties. The major economic activities are farming and fishing. Mayuge district is bordered by Lake Victoria to the south, as well as Jinja and Bugiri districts. It has a total of 33 landing sites where fishing activities take place. Uganda has a decentralized healthcare system, having both public and private health facilities.

Study population: The study targeted the four major categories of informal sector workers: 1) farmers; 2) fishermen; 3) commercial cyclists; and 4) the business community (traders and market vendors) in the study area. The informal sector was chosen because they make up the largest (80%) portion of the population, without which the sustainability of the scheme will be questionable.

Sample size: The sample size was calculated using the Kish Leslie formula (21), considering a 95% confidence interval, a 50% expected level of WTP, a 5% level of precision, a design effect of 2, and a 10% non-response rate. This gave a total sample size of 853 informal sector workers. Four focus group discussions were conducted with women (four FGDs), and three FGDs were conducted with men (in the informal sector). This number depends on the level of saturation of information. This was determined by doing preliminary analysis while still in the field.

Sampling procedure: Stratified random sampling was used in this study. For Iganga district, the informal sector workers were divided into three major categories (strata): farmers, commercial cyclists, traders/market vendors. In Mayuge district, the informal sector workers were divided into four categories: fishermen, farmers, commercial cyclists, and market vendors. A random sample from each stratum was taken in a number proportional to the stratum's size when compared to the population. In Iganga district, two sub counties were randomly selected, and five villages from each subcounty were also randomly selected. A proportionate sample of 286 households from the 10 villages was selected, and the household heads were systematically selected for interviews. Seven commercial cyclist stages located along main roads and junctions were randomly selected, and 10 cyclists available at the time of the study were randomly selected

to participate in the interviews. A total of 84 traders were selected systematically from the two main markets for interviews. In Mayuge district, a total of 10/33 landing sites were randomly selected, and 248 fishermen were selected from the 10 landing sites proportionate to size. Sixty-two traders were randomly selected from three main markets systematically, and 62 commercial cyclists were randomly selected from six stages located along main roads in Mayuge town (Table 1). Men and women from each of the categories were purposively selected for the focus group discussions. These were selected and identified with the help of local leaders based on community members who can express themselves, but also are residents and working in the informal sectors of interest.

Table 1: Sampling procedure per category of informal sector

**Iganga district (**Iganga district planning office, 2018)				
**Category**	**Farmers/peasants**	**Commercial cyclists**	**Traders/vendors**	**Fisherfolk**
Proportion in the district	0.65	0.16	0.19	
Sample size	(0.65*440) =286	(0.16*440) =70	(0.19*440) =84	**-**
Selection procedure	2 sub counties (SC) and 5 villages from each SC were randomly selected. Households are proportionate to size selected.	7 commercial cyclist stages in the town were randomly selected, cyclists on stage randomly selected to be interviewed	Traders were systematically selected for the interviews two main markets	-
Mayuge district				
**Category**	**Farmers/peasants**	**Commercial cyclists**	**Traders/vendors**	**Fisherfolk**
Proportion in the district	0.1	0.15	0.15	0.6
Sample size	(0.1*413) =41	(0.15*413) =62	(0.15*413) =62	(0.6*413) =248
Selection procedure	One sub county and two villages were randomly selected, the	6 commercial cyclist stages in town were randomly selected, later cyclists on	Traders were systematically selected from 3 main markets	10/33 landing sites were randomly selected.

	number of households were determined proportionate to size.	stage at that time were randomly selected to be interviewed		Fishermen at each site were randomly selected, proportionate to size
**Total samples**	**327**	**132**	**146**	**248**

Data collection methods and tools

The WTP was assessed using a CVM using the “bidding game” technique. The bidding game was used to determine the maximum premium that a respondent is willing to pay for the proposed NHI plan. A bidding game technique was chosen because of its ease of implementation and its minimal biases. In addition, many studies have found the bidding game to be very reliable (22, 23). The respondent was asked if he or she was willing to pay the starting bid. If the respondent agreed, the interviewer would raise the bid by 10% of the first bid and again ask if the respondent was willing to pay the new bid. The interviewer continued to present the different bids until the respondent expressed an unwillingness to pay. On the other hand, if the respondent expressed unwillingness to pay the starting bid, the interviewer lowered the bid by 10% and repeated the query. This continued until a bid was reached where the respondent was willing to pay. The starting bid was 20,000 UGX (5.4 USD), since this was the average premium for beneficiaries in the existing community-based health insurance schemes in Luweero, Western Uganda, and Jinja. The proportion of those willing to pay, the average, and the median amount of money people were willing to pay were computed. The perceived ability to pay was measured by asking

respondents whether they were able to pay premiums for health insurance per year and how much they could afford without foregoing other basic needs.

Both quantitative and qualitative data were collected co currently (Convergent parrel mixed methods). A semi-structured questionnaire was used to collect quantitative data such as sociodemographic variables, WTP, perceived ability to pay, and associated factors from informal sector workers. The authors developed the tool based on literature and their prior knowledge on NHIs. The tool also captured a few responses qualitatively. The data collection tools were translated to the local language and then back translated to English to check whether the translated questions still held informational validity. The principal investigator recruited and trained research assistants so that they were familiar with the statement of the problem, objectives of the study, sampling procedure, data collection tools, and plan for data collection. Research assistants were oriented to the bidding game technique for assessing WTP. They were also taught the meaning of health insurance and how it intends to be operationalized in the country. Research assistants were also trained in the basic interview techniques, such as asking questions in a neutral manner, not showing by words or actions what answers were being expected of the study participants, and how to record answers, especially from open-ended questions, without interpreting them. The data collection tools were pre-tested in the neighboring district, Bugweri district. This was done to test the validity and ease of application of the tools. Filled electronic forms were checked at the point of data collection for completeness, and those found incomplete were completed before the respondent was discharged. The data were cleaned and edited by the research assistants before they submitted the complete forms to the server.

Focus group discussion (FGD) guides were also used to collect data from informal sector workers to understand the determinants of willingness to pay and their perceived ability to pay for the NHI. The FGDs were audio-taped. The FGD was composed of 6–8 participants. The discussion had a moderator and a note-taker, and the discussions lasted for an average of one hour. These were conducted in the local language, and these were audio recorded.

Data management and analysis: The quantitative data were downloaded from the server and imported into STATA 14 for cleaning and analysis. Descriptive statistics, including mean, median, standard deviations, frequencies, and percentages, were obtained. The information was presented in frequency distribution tables. Principal component analysis was done to generate wealth quintiles using the demographic health survey categorization. The wealth quintiles were generated based on nine household items, which included possession of a car, motorcycle, bicycle, radio, television, mobile phone, piece of land, owning a house, and having animals. Inferential statistics were obtained using logistic regression. This was done in two stages: 1) bivariate analysis was done to determine the potential variables associated with willingness to pay for health insurance. Crude odds ratios at a 95% confidence interval were obtained to measure the potential association. 2) A multivariate analysis was done to determine the actual factors associated with WTP. All variables that were statistically significant at bivariate analysis and the key factors known in the literature to be statistically significant, such as socio-economic status, occupation, and potential confounders like residence, were included in the model. Variables were tested for multicollinearity before being included in the model. The model was built step-by-step while testing it using *lfit* until the best model was obtained. Variables that

remained statistically significant at 95% confidence were considered the factors associated with WTP for health insurance. Adjusted odds ratios were obtained as measures of association.

Qualitative data was analyzed manually using a thematic-inductive approach. The recordings were transcribed verbatim and translated from the local language (Lusoga) to English. Transcripts were read by more than two independent analysts who identified codes, sub-themes, and then organized them into themes. The information was triangulated with the quantitative data findings to gain a deeper understanding.

Ethics statement

The study was approved by the Makerere University, School of Public Health Higher Degrees, Research and Ethics Committee (HDREC). At the district level, permission to conduct the study was obtained from the local leaders and the District Health Office. Permission was also sought from subcounty chiefs and local council one chairpersons. The informed written consent of each individual participant was obtained at the start of the study. Confidentiality and anonymity were maintained by using codes.

Results

Background characteristics of the respondents

Of the 853 respondents who participated in the study, 327 (38.3%) were peasant farmers, 248929.1%) were fishermen, 146 (17.1%) were traders, and 132 (15.5%) were commercial cyclists. The mean age of the respondents was 37.0 years (SD ± 11.3). The majority 667/853 (78.2%) of the respondents were males, 783/853 (91.8%) of the household heads were males,

and 618/853 (72.4%) resided in rural areas. The average household size was five people. The median monthly income was UGX 150,000 (USD 40.5). Only 158/853 (18.5%) were in the wealthiest quintile, while 349/853 (41.0%) of the households were in the second and poorest wealth quintiles (Table 2).

Table 2: Background characteristics of the respondents

Variable	Frequency (N=853)	Percentage (%)
**Sex of respondent**		
Male	667	78.2
Female	186	21.8
**Sex of the household head**		
Male	783	91.8
Female	70	8.2
**Residence**		
Rural	618	72.4
Urban	235	27.6
**Marital status**		
Married	694	81.4
Single	85	10.0
Separated	48	5.6
Widowed	26	3.0
**Age category**		
18-25	124	14.5
26-35	303	35.5
36-45	239	28.0
46-55	126	14.8
>55	61	7.2
**Education level**		
None	51	6.0
Primary	445	52.2
Secondary	280	32.8
Tertiary	77	9.0
**Religious denominations**		
Muslim	304	35.6
Protestant	276	32.4
Catholic	194	22.7
Born again	53	6.2

SDA	26	3.1
**Occupation**		
Farmer	327	38.3
Fishermen	248	29.1
Business	146	17.1
Commercial cyclists	132	15.5
**Wealth quintiles**		
Poorest (<-1.39)	172	20.2
Poor (-1.40 - -0.50)	177	20.8
Moderate (-0.51 -0.29)	163	19.1
Wealthy (0.30 -1.23)	183	21.5
Wealthiest(1.24-4.14)	158	18.5

Willingness to pay for the proposed NHIs

The majority 695/853 (81.5%) of the respondents were willing to pay for the proposed NHIs. Of those willing to pay, the median WTP was UGX 25,000 (USD 6.8) while the mean WTP was UGX 28,950 (USD 7.8) per person, inclusive of four dependents per year. Only 3.8% protested contributing to the scheme, and 126/853 (14.7%) had zero WTP because they believed they were too poor to pay for national health insurance (Table 3).

Qualitatively, most of the FGD participants were also willing to make contributions for health insurance. However, others resented making contributions, as noted below:

> *"Sincerely, the government is meant to provide all the medical services. So, there is no way that you can lie to us and say that the government cannot provide those services..."* **(FGD male-fishermen-Mayuge)**

Several FGD participants were not willing to pay because of poverty, while others criticized the government's misuse of taxes collected from the taxpayer on salaries for officials rather than on health services, as noted in the quotes below.

“The fact is that poverty is the biggest hindrance. Because at times you can fall sick, yet you do not have money, and you resort to borrowing from colleagues who may not even be able to help you out because they also do not have. So generally, I disagree with paying for health insurance." **(FGD-Business Women, Mayuge District)**

“I am not willing to pay because we are always charged a lot of tax to register our businesses. That is all tax. Where does that tax go? Let them reduce the salary of Members of Parliament so that part of it is utilized to buy medicine. Members of Parliament have no importance in our area." (**FFGD-Male-fishermen-Mayuge district)**

Willingness to pay with copayments:

A copayment is a cost-sharing arrangement where one covers a portion of the costs out of pocket each time one visits the health facility. Less than half, 309/695 (44.5%) of the respondents, were willing to co-pay. The mean co-payment amount was UGX 2,650 (USD 0.7), with a median of UGX 2000 (USD 0.5), each time someone accessed services. The average WTP with co-payment was UGX 23,000 (USD.6.2) and the median WTP was UGX 20,000 (USD.5.4) (Table 3).

Table 3: Willingness to pay for the proposed NHI annually

**WTP**	**Frequency (%)**	**Mean**	**Median**
Protests (WTP=0)	32 (3.8)	0	0
WTP=0 (zero WTP)	126 (14.7)	0	0
WTP>0	695(81.5)	**28,950**	**25,000**
Total	853 (100.0)	23,600	20,000
**Co-payment scenario**			
Amount willing to co-pay	309/695(44.5)	**2,650**	**2,000**
WTP with co payment	309/695(44.5)	23,000	20,000

Notes: *Protests-those protested attaching value to HI (WTP=0 but it has no economic sense). WTP=0 means Zero WTP. WTP>0 that it is positive -WTP is above zero.*

Qualitatively, most of the FGD participants were not in support of co-payments, as expressed by one of the FGD participants.

> *"I also do not support the idea that we pay money each time we visit the health facility, especially when we are paying every year. This will make people unable to access the services, and hence the problem of access to services will not have been solved. People are already poor, and that will mean people will continue to sell their property to go to health facilities."* (FGD Male Farmer)

Preferred mode of payment

Most (54.1%) respondents preferred to pay annually, while 21.0% preferred to pay per harvesting season. Only 13.1% wanted to pay according to their monthly income, with the majority (63.1%) preferring to pay 1 percent of their monthly income, followed by those who were willing to pay 5%.

Perceived ability to pay for the NHIs

More than half 633/853 (74.2%) of the respondents believed that they were able to pay for health insurance, with a mean of UGX 23,000 (USD.6.2) and median of UGX 20,000 (USD.5.4) per person per year.

Regarding the perceived ability to pay, most of the FGD participants noted that most of the people are poor and most likely may not be able to make periodic subscriptions, especially if the money is raised more than what people earn. This was attested to, as indicated below:

> *"Sincerely speaking, some people cannot afford to pay money; for instance, you may find a very old person who is no longer earning anything, yet they also need health care."* **(FGD-commercial cyclist-Iganga district)**

Another FGD participant, who was a business lady, noted that

> *"I support the idea. But not very much because of the issue of affordability. Not everyone can afford to pay and note that we earn differently. Because some of us play the role of mother and father in the family, there are a lot of requirements to provide."* **(FGD Businesswomen, Iganga)**

Furthermore, most of the participants noted that setting premiums should be based on people's income and ability to pay, as noted below:

> *"Since everyone earns differently, the charges should depend on how much someone earns. I could be able to afford UGX 50,000, but my colleague cannot at all since people in the village are used to getting free medicine. When some people are asked to pay just 500shs for drugs, they will consider it unworthy because they know that drugs/medication are freely given. So, if possible, we charge according to people's income."* **(FGD: businesswomen, Iganga district)**.

Determinants for level of WTP for the proposed NHIs

At bi-variable analysis, wealth quintiles, savings group membership, having a family member with chronic illness, use of traditional medicine, and hearing about health insurance were statistically significant for willingness to pay for health insurance. The odds of being willing to pay when in

the fourth wealth quintile were 2.88, compared to the odds of being willing to pay among those in the poorest category (COR: 2.88, 95% CI: 1.60–5.18, P<0.001). Not being in the saving group reduced the chances of being willing to pay for health insurance by 42% compared to those who had saving group memberships (COR: 0.58, 95% CI: 0.33-0.69, P<0.001) (Table 4).

After adjusting for any confounding factors, occupation, wealth, saving group membership, use of traditional medicine, and having heard about health insurance were significantly associated with the level of WTP for the proposed NHIs.

The odds of fishermen's WTP for NHIs were 1.70 compared to those of farmers after adjusting for other factors (AOR: 1.70, 95% CI: 1.04–2.79, P = 0.035). Respondents in the fourth wealth quintile were 2.98 times more likely to pay for NHIs compared to those in the poorest wealth quintile (AOR: 2.98, 95% CI: 1.56–5.65). Respondents who were not in any saving group were 0.51 times less likely to pay for the proposed NHIs compared to those with saving group membership (AOR: 0.51, 95% CI: 0.34-0.76, P<0.001), and individuals who had never heard about HI were 50% less willing to pay for the proposed NHIs compared to those who had ever heard about health insurance (AOR: 0.50, 95% CI: 0.23-0.86, P = 0.032) (Table 4).

Table 1: Multi variable analysis of factors associated with WTP for NHI scheme.

**Variable**	**Level of Willingness to pay**		**COR (95%CI)**	**AOR (95%CI)**
	**No (n=158)**	**Yes (n=695)**		
**Sex of respondent**				
Male	127(80.4)	540(77.7)	1.0	
Female	31(19.6)	155(22.3)	1.18(0.76-1.81)	1.30(0.80-2.11)
**Residence**				
Rural	110(69.6)	508(73.1)	1.0	

Urban	48(30.4)	187(26.9)	0.84(0.58-1.23)	1.09(0.52-2.26)
**Occupation**				
Farmer	60(38.0)	267(38.4)	1.0	
Business	32(20.2)	114(15.4)	0.80(0.49-1.30)	0.59(0.26-1.35)
Commercial cyclist	25(15.8)	107(15.4)	0.96(0.57-1.61)	1.04(0.50-2.18)
Fisherfolk	41(26.0)	207(29.8)	1.13(0.73-1.76)	1.70(1.04-2.79) *
**Wealth quintiles**				
Poorest	43(27.2)	129(18.6)	1.0	
Second poorest	32(20.3)	145(20.9)	1.51(0.90-2.53)	1.83(1.05-3.19) *
Middle	35(22.1)	128(18.4)	1.22(0.73-2.03)	1.23(0.70-2.14)
Fourth	19(12.0)	164(23.6)	2.88(1.60-5.18)	2.98(1.58-5.65) *
Wealthiest	29(18.4)	129(18.6)	1.48(0.87-2.52)	1.53(0.81-2.88)
**In saving group**				
Yes	49(31.0)	337(48.5)	1.0	
No	109(69.0)	358(51.5)	0.48(0.33-0.69)	0.51(0.34-0.76) **
**Family member with chronic illness**				
Yes	36(22.7)	241(34.7)	1.0	
No	122(77.2)	576(67.5)	0.56(0.37-0.83)	0.73(0.46-1.14)
**Ability to meet health care**				
Difficult	112(70.9)	571(82.2)	1.0	
Easy	46(29.1)	124(17.8)	0.53(0.36-0.78)	0.68(0.43-1.05)
**Use Traditional Medicine**				
No	59(38.8)	345(51.4)	1.0	
Yes	93(61.2)	326(48.6)	0.60(0.42-0.86)	0.71(0.48-0.92) *
**Ever heard about NHI**				
**Yes**	9(5.7)	83(11.9)	1.0	
**No**	149(94.3)	612(88.1)	0.45(0.22-0.91)	0.50(0.23-0.86) *

Note: *** p<0.001, *P<0.05*

Discussion

This study assessed the WTP and perceived ability to pay for the proposed NHIs and its determinants among informal sector workers in Iganga and Mayuge districts, Uganda. The study found that most informal sector workers were both willing and believed they were able to pay for the proposed NHI. WTP was significantly associated with occupation, wealth, awareness, and membership in saving groups.

In this study, most of the informal sector workers were willing to make contributions to the proposed NHIs. This could be perceived as support for the proposed NHIs among the informal sector workers. Our study findings are consistent with similar studies conducted in in Nepal (71%) and in South Sudan (68%) among the informal sector (13, 24). Similarly, a systematic review and meta-analysis of WTP found that the pooled WTP for NHI in Africa and Asia was 71.0% (25). It is also consistent with studies conducted in Togo (92%) (26) and 93% in Sierra Leone (27). The high level of WTP contributes to growing evidence showing the potential of informal sector workers participating in NHIs despite their nature of having unpredictable incomes and always poor (28).

This study found a median WTP of USD 6.8 and a mean WTP of USD 7.8 per person per year, which includes coverage for four dependents. This was slightly higher than the starting bid of USD 5.4 per year, thus the informal sector workers were willing to contribute more than the initially hypothesized amount. However, the WTP was lower than the 5.5 USD per person per month in Iran, 6.6 USD per person per month in Namibia, and 3 USD in Kenya per month (18, 24, 29). These discrepancies in WTP may reflect variations in socio-economic status and differing perceptions of health insurance value among study populations. As Uganda deliberates (as of 2025) the NHIs bill, it is important to recognize the relatively low WTP among informal sector workers compared to other contexts. This underscores the need to design affordable premium

structures tailored to the financial realities of this group. The informal sector, often characterized by irregular and unpredictable incomes, may struggle to afford fixed, high premiums. In Ethiopia, making premium systems more flexible eased payments (30).

In this study, membership in local savings groups was associated with a higher likelihood of WTP for the proposed NHIs. In Asian countries, saving groups were reported to be key in initiating community-based health insurance schemes (31). Savings groups provide members with a secure place to save money, generate a pool of funds, and offer loans to members in times of shock and also enable the community to meet the premiums (31). Given their widespread presence in Uganda and community trust (32), these groups present an opportunity to be leveraged on to mobilize the informal sector to save and contribute to the proposed NHIs.

Participants belonging to the highest wealth category were more likely to pay for the proposed national health insurance scheme. This is in agreement with studies conducted in Nigeria, Ethiopia, and Ghana, which have reported that socio-economic factors such as household income and wealth status have a bearing on the ease or difficulty of enrolling in a given scheme, with high premiums deterring enrollment in schemes (33-35). Similar studies conducted in Sierra Leone and Iran showed that the WTP for the HI scheme depended on the monthly income of the respondent (27, 29) . In Nepal, belonging to a lower wealth quartile was associated with a lower willingness to pay for National Health Insurance Scheme (36). These findings underscore that WTP for NHIs is closely tied to household economic status. Thus, the WTP for NHIs unavoidably depends on the wealth and incomes of the individuals. Efforts to improve household income levels will be essential to ensure broad participation in the proposed NHIs.

The use of traditional medicine was negatively associated with participation in the health insurance scheme. This may be due to a perceived lack of need for formal health services among those who rely on traditional medicine. Studies have shown that the presence and use of alternative medicine and other forms of healthcare, such as herbalists, negatively affects participation in a health insurance scheme, as NHIs are typically designed around access to and utilization of modern medical services (37). Studies conducted in Cameroon, Burkina Faso, Nigeria, and India have reported that those who use modern medicine are more willing to pay for health insurance than those who use traditional medicine (38-40). In Uganda's pluralistic health system, the ministry of health and other mandated regulatory bodies need to institute measures that not only regulate the alternative sector but also promote the utilization of modern healthcare services through education, trust-building, and service quality improvements.

The study noted that those who had never heard about NHIs were 50% less likely to pay compared to those who had prior information about health insurance. The level of knowledge on how health insurance operates was extremely low, and statistical tests of significance could not be used in this study. This was similar to studies conducted in other Africa and Asia where the knowledge was reported to be low (25). A systematic review and meta-analysis of factors influencing WTP for voluntary contributary health insurance schemes in LMICs revealed that knowledge and understanding of the functioning of the scheme positively influence the WTP and participation in the scheme (41, 42).

Implications for policy and practice

This study offers valuable insights for the ongoing design of Uganda's NHIs, especially regarding the premiums and including the informal sector. While the WTP among informal sector workers is promising, the modest contribution amounts suggest that affordability must be central to scheme design. The MoH should adopt income-sensitive premiums and flexible payment schedules, aligned with seasonal income flows such as post-harvest periods for agricultural workers. The MoH should design targeted subsidies for the poorest households to avoid exacerbating existing health inequities.

The existing local savings groups offer an opportunity to leverage on to facilitate premium collection, raise awareness, and foster a sense of local ownership. The MoH and partners should map these groups, build their capacity and support them, as a steppingstone to roll out NHIs.

Low awareness and understanding of health insurance among the informal sector pose a significant barrier to participation in NHIs. The MoH, in collaboration with district health teams, should invest in sustained awareness campaigns using trusted channels such as local radio, community meetings, and influential local leaders. Messaging should demystify the concept of health insurance, explain how contributions are used, and highlight the benefits of enrollment. The government should also foster trust among the population through improving quality of care and accountability mechanisms.

Finally, further research should assess the actual ability to pay among informal sector workers and explore how broader social health protection mechanisms can be extended to this group effectively.

Study limitations: As with most contingent valuation studies, the elicitation technique is always subject to bias, and the assignment of the first bid or amount is also prone to bias. However, this was triangulated with open-ended questions and qualitative data. The low level of knowledge about health insurance among respondents was another limitation in attaching value to the proposed scheme. This was addressed by educating the respondents about health insurance before eliciting their willingness to pay. Despite these limitations, the study provides valuable insights into the WTP for the proposed NHIs among informal sector workers, who are often excluded from social protection programs.

Conclusions

This study indicated that the informal sector workers are willing to make contributions to the proposed NHIs. However, the premium structure should be designed with careful consideration of the ability of the informal sector to pay, given the high levels of poverty and unpredictable incomes. The study also highlights that occupation, wealth status, use of traditional medicine, prior knowledge of health insurance and trust influence WTP for NHIs among the informal sector workers. Creating awareness and fostering trust is critical to the success of the proposed NHIs.

Declarations

Consent for publication

Not Applicable

Availability of data and materials

The dataset analyzed for this study has been uploaded as a supplementary file.

Competing interests

The authors declare that they have no competing interests

Funding

The author(s) received no specific funding for this work.

Authors' contributions

NN, SNK, AK conceived the study, NN collected, analyzed, and wrote the first draft of the manuscript. SNK and AK provided reviews on all drafts of manuscript. All the authors read and approved the final manuscript.

Acknowledgements

We thank the District Health Officers of Mayuge and Iganga districts for their cooperation, mobilization and ensuring that data collection went on smoothly. We also extend our sincere appreciation to the research assistants and all respondents in Iganga and Mayuge for sparing their time to contribute to this piece of work.

References

1. WHO. Universal health coverage (UHC): World Health Organization 2023 [Available from: https://www.who.int/news-room/fact-sheets/detail/universal-health-coverage-(uhc).

2. WHO. Health Systems Financing: The Path to Universal Coverage. Geneva: World Health Organization 2010.

3. World Bank. Financing health in low-income countries. Washington DC: World Bank Group; 2010.

4. WHO. Noncommunicable diseases: World Health Organization 2024 [Available from: https://www.who.int/news-room/fact-sheets/detail/noncommunicable-diseases.

5. MoH. National Health Accounts 2018/19-2020/21 Report. 2021.

6. Tashobya CK, Ssengooba F, Cruz VO, Yates R, Murindwa G, McPake B. Health systems reforms in Uganda: processes and outputs. Christine Kirunga Tashobya FS and, Valeria Oliveira Cruz, editors. 2006.

7. UBOS. The National Population and Housing Census 2014 – Main Report. Kampala, Uganda: Uganda Bureau of Statistics; 2016.

8. Twaweza. Health Check: Ugandans' experiences and opinions on health services Uganda: Twaweza; 2019 [Available from: https://www.twaweza.org/go/szw-ug-health-check.

9. Gottret P, Schieber G. Health financing revisited: a practitioner's guide: The World Bank; 2006.

10. Xu K, Evans DB, Carrin G, Aguilar-Rivera AM, Musgrove P, Evans T. Protecting households from catastrophic health spending. Health affairs. 2007;26(4):972-83.

11. De Allegri M, Sanon M, Bridges J, Sauerborn R. Understanding consumers' preferences and decision to enrol in community-based health insurance in rural West Africa. Health policy. 2006;76(1):58-71.

12. Kyomugisha E, Buregyeya E, Ekirapa E, Mugisha J, Bazeyo W. Strategies for sustainability and equity of prepayment health schemes in Uganda. African health sciences. 2009;9(2).

13. Basaza R, Alier PK, Kirabira P, Ogubi D, Lako RLL. Willingness to pay for National Health Insurance Fund among public servants in Juba City, South Sudan: a contingent evaluation. International journal for equity in health. 2017;16(1):158.

14. Nguyen H, Knowles J. Demand for voluntary health insurance in developing countries: the case of Vietnam's school-age children and adolescent student health insurance program. Social Science & Medicine. 2010;71(12):2074-82.

15. Acharya A, Vellakkal S, Taylor F, Masset E, Satija A, Burke M, et al. Impact of National Health Insurance for the Poor and the Informal sector in Low-and Middle-Income Countries. London: EPPI-Centre, Social Science Research Unit, Institute of Education, University of London; 2012.

16. Basaza R, Criel B, Van der Stuyft P. Community health insurance in Uganda: why does enrolment remain low? A view from beneath. Health policy. 2008;87(2):172-84.

17. Jowett M, Kutzin J, Organization WH. Raising revenues for health in support of UHC: strategic issues for policy makers. World Health Organization; 2015.

18. Gustafsson-Wright E, Asfaw A, van der Gaag J. Willingness to pay for health insurance: An analysis of the potential market for new low-cost health insurance products in Namibia. Social science & medicine. 2009;69(9):1351-9.

19. Russell S, Fox-Rushby J, Arhina D. Willingness and ability to pay for health care: a selection of methods and issues. Health Policy and Planning. 1995;10(1):94-101.

20. Mitchell RC, Carson RT. Using surveys to value public goods: the contingent valuation method: Rff Press; 2013.

21. Kish L. Survey sampling. Inc., New York.: John Wiley and Sons; 1965.

22. Whittington D, Smith VK, Okorafor A, Okore A, Liu JL, McPhail A. Giving respondents time to think in contingent valuation studies: a developing country application. Journal of Environmental Economics and Management. 1992;22(3):205-25.

23. Dong H, Kouyate B, Cairns J, Sauerborn R. A comparison of the reliability of the take-it-or-leave-it and the bidding game approaches to estimating willingness-to-pay in a rural population in West Africa. Social Science & Medicine. 2003;56(10):2181-9.

24. Barasa EW, Mwaura N, Rogo K, Andrawes L. Extending voluntary health insurance to the informal sector: experiences and expectations of the informal sector in Kenya. Wellcome open research. 2017;2.

25. Bayked EM, Assfaw AK, Toleha HN, Zewdie S, Biset G, Ibirongbe DO, et al. Willingness to pay for National Health Insurance Services and Associated Factors in Africa and Asia: a systematic review and meta-analysis. Frontiers in Public Health. 2024;12:1390937.

26. Djahini-Afawoubo DM, Atake E-H. Extension of mandatory health insurance to informal sector workers in Togo. Health economics review. 2018;8:1-13.

27. Jofre-Bonet M, Kamara J. Willingness to pay for health insurance in the informal sector of Sierra Leone. PloS one. 2018;13(5):e0189915.

28. ILO. Women and men in the informal economy: A statistical picture. Geneva ILO; 2018.

29. Nosratnejad S, Rashidian A, Mehrara M, Sari AA, Mahdavi G, Moeini M. Willingness to pay for social health Insurance in Iran. Global journal of health science. 2014;6(5):154.

30. Habte A, Tamene A, Ejajo T, Dessu S, Endale F, Gizachew A, et al. Towards universal health coverage: The level and determinants of enrollment in the Community-Based Health Insurance

(CBHI) scheme in Ethiopia: A systematic review and meta-analysis. PloS one. 2022;17(8):e0272959.

31. Shaikh BT, Noorani Q, Abbas S. Community based saving groups: an innovative approach to overcome the financial and social barriers in health care seeking by the women in the rural remote communities of Pakistan. Archives of Public Health. 2017;75(1):57.

32. Mutebi A, Muhumuza Kananura R, Ekirapa-Kiracho E, Bua J, Namusoke Kiwanuka S, Nammazi G, et al. Characteristics of community savings groups in rural Eastern Uganda: opportunities for improving access to maternal health services. Global health action. 2017;10(sup4):1347363.

33. Kotoh AM, Aryeetey GC, Van der Geest S. Factors that influence enrolment and retention in Ghana'National Health Insurance Scheme. International journal of health policy and management. 2018;7(5):443.

34. Odeyemi IA. Community-based health insurance programmes and the national health insurance scheme of Nigeria: challenges to uptake and integration. International journal for equity in health. 2014;13(1):20.

35. Mebratie AD, Sparrow R, Yilma Z, Alemu G, Bedi AS. Enrollment in Ethiopia's community-based health insurance scheme. World Development. 2015;74:58-76.

36. Khatiwada B, Ghimire S, N S. Willingness to pay for health insurance in mangalbare village development committee of illam district. MOJ Public Health. 2017;5(2):43-6.

37. Sarkodie AO. Effect of the National Health Insurance Scheme on healthcare utilization and out-of-pocket payment: evidence from GLSS 7. Humanities and Social Sciences Communications. 2021;8(1):1-10.

38. Mathiyazaghan K. Willingness to pay for rural health insurance through community participation in India. The International journal of health planning and management. 1998;13(1):47-67.

39. Donfouet HPP, Mahieu P-A, Malin E. Using respondents' uncertainty scores to mitigate hypothetical bias in community-based health insurance studies. The European Journal of Health Economics. 2013;14(2):277-85.

40. Oriakhi H, Onemolease E. Determinants of rural household's willingness to participate in community based health insurance scheme in Edo State, Nigeria. Studies on ethno-medicine. 2012;6(2):95-102.

41. Adebayo EF, Uthman OA, Wiysonge CS, Stern EA, Lamont KT, Ataguba JE. A systematic review of factors that affect uptake of community-based health insurance in low-income and middle-income countries. BMC health services research. 2015;15(1):543.

42. Dror DM, Hossain SS, Majumdar A, Koehlmoos TLP, John D, Panda PK. What factors affect voluntary uptake of community-based health insurance schemes in low-and middle-income countries? A systematic review and meta-analysis. PLoS One. 2016;11(8):e0160479.

Manuscript Click here to access/download;Manuscript;Clean Manuscript 13th May 2025 Final.docx

Willingness and perceived ability to pay for Uganda's proposed national health insurance scheme among informal sector workers in Iganga and Mayuge districts, Uganda: a contingent valuation method

Noel Namuhani[1*], Angela N Kisakye[1, 2] and Suzanne N Kiwanuka[1]

Institutional affiliations

[1]Makerere university school of public health, Department of health policy planning and management, Uganda

[2]African Field Epidemiology Network, Lugogo House, Plot 42, Lugogo By-Pass

*Corresponding author

Email: nnamuhani@musph.ac.ug

Abstract

Background: Access to healthcare remains a challenge, particularly among informal sector workers in most low-income countries, due to high out-of-pocket expenditures (OOPE). In Uganda, over 28% of healthcare costs are paid out of pocket. In response, Uganda has proposed a national health insurance scheme (NHIs). However, the willingness and ability to pay for the proposed NHIs within the informal sector have not yet been explored in Uganda. This study assessed the willingness and perceived ability to pay for the proposed NHIs, along with its determinants, among informal sector workers in Iganga and Mayuge districts, Uganda.

Methodology: A cross-sectional study was conducted in Iganga and Mayuge districts between 10/04/2019 and 23/05/2019. A contingent valuation method using the bidding game technique was used to elicit the willingness to pay (WTP). A total of 853 informal sector workers, including farmers, commercial motorists, fishermen, and traders, were randomly sampled. Seven focus group discussions (FGD) were also conducted. Logistic regression was done to identify the determinants of WTP for the proposed NHIs. Qualitative data was analyzed thematically.

Results: The majority 695/853, (81.5%) of the respondents were willing to pay for NHIs; the median WTP was UGX 25,000 (USD 6.8) annually; and 633/853, (74.2%) of the respondents believed that they were able to pay for the NHIs. WTP was significantly associated with being a fisher folk (AOR: 1.70 95%CI: 1.04-2.79, P = 0.035), being in the fourth wealth quintile (AOR: 2.98, 95% CI: 1.56–5.65), not hearing about health insurance (AOR: 0.50 95%CI: 0.23-0.86, P = 0.032), and not having saving group membership (AOR: 0.51, 95%CI: 0.34-0.76, P<0.001). Most of the

FGD participants were willing to pay for the proposed scheme; however, some of the participants doubted their ability to pay given their extreme poverty levels and their unstable income.

Conclusion: The WTP for the NHIs in the informal sector was high. Therefore, it is viable for the government to extend NHIs to the informal sector. However, building awareness and consideration of extreme poverty levels in setting appropriate premiums should be a priority.

Key words: National health insurance scheme, willingness to pay, ability to pay, informal sector, contingent valuation method.

Author summary

Many Ugandans, especially those in the informal sector, struggle to access healthcare due to high out-of-pocket healthcare costs. To address this, Uganda plans to establish a national health insurance scheme. However, the extent to which informal sector workers, who constitute the largest portion of the labor force, are willing and able to pay remains unclear. This paper, therefore, seeks to explore the willingness and ability of informal sector workers to pay for the proposed national health insurance scheme.

To answer this objective, we interviewed 853 workers engaged in farming, fishing, trade, and commercial cycling in Iganga and Mayuge districts. We found that most respondents (82%) were willing to pay for the proposed health insurance, with an average annual amount of UGX 25,000 (about USD 6.80). Over 74% believed they could afford to pay for the proposed scheme, while others expressed concerns about their ability to pay due to poverty and unstable incomes.

Our study findings suggest that extending the health insurance scheme to informal sector workers is feasible. However, the government needs to consider affordability when setting premiums, given the elevated levels of poverty.

Introduction

Globally, there is a growing commitment to achieving Universal Health Coverage (UHC), which is an important element for achieving the Sustainable Development Goals (SDGs) (1). UHC aims to address the poor access to quality and affordable health services, which many African countries are grappling with, mainly due to high out-of-pocket expenditures (OOPE) (2). In 2021, it was estimated that about 4.5 billion people (more than half) in the world were not fully covered by essential health services, while about 2 billion (a quarter) of the world's population were facing financial hardship due to out-of-pocket health spending (1).This put about 150 million people at risk of catastrophic healthcare costs annually, driving over 100 million people into poverty worldwide (2). According to the World Bank, direct modes of payment for health care services constitute 40% of health care expenditure in developing countries (3). However, developing countries suffer a higher burden of preventable disease, including the rising non communicable diseases (4). Thus, the need for health financing reforms to address this burden of disease, reduce OOPE and inequities.

In Uganda, although the national health accounts reported a reduction in the OOPE on health as a share of current health expenditure (CHE) from 38.6% in the financial year 2018/2019 to 27.4% in the financial year 2020/2021, the OOPE remains significantly high. This is compounded by the complex health financing landscape, where the general government health expenditure per

capita allocation is very low estimated at 11.6 $, heavy reliance on donor funding accounting for 50.3% of the total health expenditure which has significantly reduced over the years. (5). Uganda abolished user fees in public health facilities, however, accessing healthcare is still associated with several expenses, including transport, drugs, diagnostics, and consultancy fees, among other expenses related to personnel and capital costs (6).

Globally, more than 60% of the employment occurs in the informal sector and in Uganda, over 80% of the labor force belongs to the informal sector (7). These include peasant farmers, commercial cyclists, fishermen, and those employed in unregistered or small-scale enterprises. It is estimated that 59% of the people in the informal sector in Eastern Uganda cannot afford to access quality health care due to high poverty levels and OOPE for health care (8).

Globally, the implementation of health insurance schemes is one of the major responses to addressing OOPE on health care (2, 9) in low and middle-income countries. Health insurance has the ability to reduce financial barriers to health care access and protect individuals and families against the risk of unpredictable health care expenditures, hence aiding the attainment of UHC (10).

High coverage and enrollment in the proposed NHIs are critical for the success and sustainability of the scheme, given that premiums constitute the main proposed source of funding for the scheme. However, enrolment in health insurance schemes remains low, ranging from 2% to 40% in most African countries (11-13). The poor enrolment rates in the different countries have been attributed to a number of factors, including low incomes, large household sizes, long distances to health facilities, limited awareness, poor quality of health care services, inappropriate benefit

packages, a lack of trust in the systems, and high illiteracy levels, especially in the informal sector (14-16). The informal sector workers are sometimes of low socioeconomic status compared to the formal sector workers, hence having a lower ability to pay for health insurance (16). Furthermore, the informal sector is not well organized; they have unpredictable incomes, making it difficult to enroll and register them into the NHIs and collect regular contributions from them (13, 17).

Uganda is in the process of developing a NHIs to address the high OOPE and access to services. However, given the high poverty levels among Ugandans and more so in the informal sector, the policy question of WTP and their ability to pay remains unanswered. Eastern Uganda is the poorest region in Uganda, with 35.7% of most households living below the poverty line (7). The WTP for the proposed NHIs, especially within the informal sector, which makes up 80% of Uganda's population, is critical to the achievement of equity in access to care and the core risk pooling function of a health insurance scheme. This would translate into high enrollment rates, which foster the financial sustainability of the schemes (10).

The NHI Bill, 2019, proposes that the scheme will be mandatory for all Ugandans, covering both formal and informal sector workers. Each person will be required to pay a premium, the amount of which will be determined by the board, to access the scheme. The main sources of funding for the proposed scheme are premium contributions and government contributions for the indigent. Services will be provided in both public and accredited private health facilities. The bill was passed by Parliament in 2021 but was not assented to by the president due to disagreements

among stakeholders who were not consulted. As of 2025, the bill is being discussed by the MoH and other stakeholders before being re-tabled in the Parliament of Uganda.

There is a paucity of information on the willingness and ability to pay for the proposed NHIs scheme, particularly within the informal sector. Understanding the WTP and the associated factors is crucial in designing strategies to improve enrollment rates in the scheme. Therefore, this study aimed to document WTP and the perceived ability to pay for the proposed NHIs, along with the factors influencing WTP among informal sector workers. The study's findings will be used by the Ministry of Health (MOH) to determine affordable premiums and design strategies to ensure maximum participation, especially among informal sector workers. The findings will also inform the NHIs bill which is currently under discussion.

Materials and methods

Study design: This was a cross-sectional study design that involved mixed methods of data collection (convergent parrel mixed methods design). The quantitative and qualitative methods were conducted concurrently. The study was conducted from 10/04/2019 to 23/05/2019. This study adopted a contingent valuation method (CVM) using the bidding game technique to directly elicit or measure willingness to pay for a hypothetical (not yet on the market) health insurance package (18). This approach has been used because it has been proven to be an effective way of assessing WTP with minimal bias for goods that are not presently available on the market. CVM creates a hypothetical marketplace in which no actual transactions are made; hence, this approach has been successfully used for commodities that are not exchanged in regular markets. This approach makes the assumption that people have had no previous

experience buying the health service that is going to be put on the market and instead asks people their WTP based on their expectations and the benefits attached to the service (19). In the absence of markets for public goods, this method presents consumers with hypothetical markets in which they can buy a health insurance policy. Since the elicited WTP values are contingent upon the particular hypothetical market described to the respondent, this approach is called the contingent valuation method (20).

Study setting: The study was conducted in the Iganga and Mayuge districts of eastern Uganda. Iganga and Mayuge districts were selected purposively because they are the most poverty-hit districts in the country and 80% of the population lives in rural areas. They also have 80–85% of the population belonging to the informal sector (7). Iganga District is bordered by Kaliro District to the north, Bugweri District to the east, Mayuge District to the south, Jinja District to the southwest, and Luuka District to the west. With a total population of 504,197 people, 9.9% of the households are 5 km or more from the nearest health facility, and 19.9% are near a public health facility (UBOS, 2016). It is composed of 3 counties, 8 sub counties, 66 parishes, and 395 villages. The major economic activity is farming. Mayuge district is in south-eastern Uganda with a population of 473,2394 and a growth rate of 3.5%, and it has thirteen sub counties. The major economic activities are farming and fishing. Mayuge district is bordered by Lake Victoria to the south, as well as Jinja and Bugiri districts. It has a total of 33 landing sites where fishing activities take place. Uganda has a decentralized healthcare system, having both public and private health facilities.

Study population: The study targeted the four major categories of informal sector workers: 1) farmers; 2) fishermen; 3) commercial cyclists; and 4) the business community (traders and market vendors) in the study area. The informal sector was chosen because they make up the largest (80%) portion of the population, without which the sustainability of the scheme will be questionable.

Sample size: The sample size was calculated using the Kish Leslie formula (21), considering a 95% confidence interval, a 50% expected level of WTP, a 5% level of precision, a design effect of 2, and a 10% non-response rate. This gave a total sample size of 853 informal sector workers. Four focus group discussions were conducted with women (four FGDs), and three FGDs were conducted with men (in the informal sector). This number depends on the level of saturation of information. This was determined by doing preliminary analysis while still in the field.

Sampling procedure: Stratified random sampling was used in this study. For Iganga district, the informal sector workers were divided into three major categories (strata): farmers, commercial cyclists, traders/market vendors. In Mayuge district, the informal sector workers were divided into four categories: fishermen, farmers, commercial cyclists, and market vendors. A random sample from each stratum was taken in a number proportional to the stratum's size when compared to the population. In Iganga district, two sub counties were randomly selected, and five villages from each subcounty were also randomly selected. A proportionate sample of 286 households from the 10 villages was selected, and the household heads were systematically selected for interviews. Seven commercial cyclist stages located along main roads and junctions were randomly selected, and 10 cyclists available at the time of the study were randomly selected

to participate in the interviews. A total of 84 traders were selected systematically from the two main markets for interviews. In Mayuge district, a total of 10/33 landing sites were randomly selected, and 248 fishermen were selected from the 10 landing sites proportionate to size. Sixty-two traders were randomly selected from three main markets systematically, and 62 commercial cyclists were randomly selected from six stages located along main roads in Mayuge town (Table 1). Men and women from each of the categories were purposively selected for the focus group discussions. These were selected and identified with the help of local leaders based on community members who can express themselves, but also are residents and working in the informal sectors of interest.

Table 1: Sampling procedure per category of informal sector

**Iganga district (**Iganga district planning office, 2018)				
**Category**	**Farmers/peasants**	**Commercial cyclists**	**Traders/vendors**	**Fisherfolk**
Proportion in the district	0.65	0.16	0.19	
Sample size	(0.65*440) =286	(0.16*440) =70	(0.19*440) =84	**-**
Selection procedure	2 sub counties (SC) and 5 villages from each SC were randomly selected. Households are proportionate to size selected.	7 commercial cyclist stages in the town were randomly selected, cyclists on stage randomly selected to be interviewed	Traders were systematically selected for the interviews two main markets	-
Mayuge district				
**Category**	**Farmers/peasants**	**Commercial cyclists**	**Traders/vendors**	**Fisherfolk**
Proportion in the district	0.1	0.15	0.15	0.6
Sample size	(0.1*413) =41	(0.15*413) =62	(0.15*413) =62	(0.6*413) =248
Selection procedure	One sub county and two villages were randomly selected, the	6 commercial cyclist stages in town were randomly selected, later cyclists on	Traders were systematically selected from 3 main markets	10/33 landing sites were randomly selected.

	number of households were determined proportionate to size.	stage at that time were randomly selected to be interviewed		Fishermen at each site were randomly selected, proportionate to size
**Total samples**	**327**	**132**	**146**	**248**

Data collection methods and tools

The WTP was assessed using a CVM using the “bidding game” technique. The bidding game was used to determine the maximum premium that a respondent is willing to pay for the proposed NHI plan. A bidding game technique was chosen because of its ease of implementation and its minimal biases. In addition, many studies have found the bidding game to be very reliable (22, 23). The respondent was asked if he or she was willing to pay the starting bid. If the respondent agreed, the interviewer would raise the bid by 10% of the first bid and again ask if the respondent was willing to pay the new bid. The interviewer continued to present the different bids until the respondent expressed an unwillingness to pay. On the other hand, if the respondent expressed unwillingness to pay the starting bid, the interviewer lowered the bid by 10% and repeated the query. This continued until a bid was reached where the respondent was willing to pay. The starting bid was 20,000 UGX (5.4 USD), since this was the average premium for beneficiaries in the existing community-based health insurance schemes in Luweero, Western Uganda, and Jinja. The proportion of those willing to pay, the average, and the median amount of money people were willing to pay were computed. The perceived ability to pay was measured by asking

respondents whether they were able to pay premiums for health insurance per year and how much they could afford without foregoing other basic needs.

Both quantitative and qualitative data were collected co currently (Convergent parrel mixed methods). A semi-structured questionnaire was used to collect quantitative data such as sociodemographic variables, WTP, perceived ability to pay, and associated factors from informal sector workers. The authors developed the tool based on literature and their prior knowledge on NHIs. The tool also captured a few responses qualitatively. The data collection tools were translated to the local language and then back translated to English to check whether the translated questions still held informational validity. The principal investigator recruited and trained research assistants so that they were familiar with the statement of the problem, objectives of the study, sampling procedure, data collection tools, and plan for data collection. Research assistants were oriented to the bidding game technique for assessing WTP. They were also taught the meaning of health insurance and how it intends to be operationalized in the country. Research assistants were also trained in the basic interview techniques, such as asking questions in a neutral manner, not showing by words or actions what answers were being expected of the study participants, and how to record answers, especially from open-ended questions, without interpreting them. The data collection tools were pre-tested in the neighboring district, Bugweri district. This was done to test the validity and ease of application of the tools. Filled electronic forms were checked at the point of data collection for completeness, and those found incomplete were completed before the respondent was discharged. The data were cleaned and edited by the research assistants before they submitted the complete forms to the server.

Focus group discussion (FGD) guides were also used to collect data from informal sector workers to understand the determinants of willingness to pay and their perceived ability to pay for the NHI. The FGDs were audio-taped. The FGD was composed of 6–8 participants. The discussion had a moderator and a note-taker, and the discussions lasted for an average of one hour. These were conducted in the local language, and these were audio recorded.

Data management and analysis: The quantitative data were downloaded from the server and imported into STATA 14 for cleaning and analysis. The dataset has been uploaded as a supporting file, where columns represent the variable and variable names, whole the rows represent the responses. Descriptive statistics, including mean, median, standard deviations, frequencies, and percentages, were obtained. The information was presented in frequency distribution tables. Principal component analysis was done to generate wealth quintiles using the demographic health survey categorization. The wealth quintiles were generated based on nine household items, which included possession of a car, motorcycle, bicycle, radio, television, mobile phone, piece of land, owning a house, and having animals. Inferential statistics were obtained using logistic regression. This was done in two stages: 1) bivariate analysis was done to determine the potential variables associated with willingness to pay for health insurance. Crude odds ratios at a 95% confidence interval were obtained to measure the potential association. 2) A multivariate analysis was done to determine the actual factors associated with WTP. All variables that were statistically significant at bivariate analysis and the key factors known in the literature to be statistically significant, such as socio-economic status, occupation, and potential confounders like residence, were included in the model. Variables were tested for multicollinearity before being included in the model. The model was built step-by-step while testing it using *lfit* until the best

model was obtained. Variables that remained statistically significant at 95% confidence were considered the factors associated with WTP for health insurance. Adjusted odds ratios were obtained as measures of association.

Qualitative data was analyzed manually using a thematic-inductive approach. The recordings were transcribed verbatim and translated from the local language (Lusoga) to English. Transcripts were read by more than two independent analysts who identified codes, sub-themes, and then organized them into themes. The information was triangulated with the quantitative data findings to gain a deeper understanding.

Ethics statement

The study was approved by the Makerere University, School of Public Health Higher Degrees, Research and Ethics Committee (HDREC). At the district level, permission to conduct the study was obtained from the local leaders and the District Health Office. Permission was also sought from subcounty chiefs and local council one chairpersons. The informed written consent of each individual participant was obtained at the start of the study. Confidentiality and anonymity were maintained by using codes.

Results

Background characteristics of the respondents

Of the 853 respondents who participated in the study, 327 (38.3%) were peasant farmers, 248929.1%) were fishermen, 146 (17.1%) were traders, and 132 (15.5%) were commercial cyclists. The mean age of the respondents was 37.0 years (SD ± 11.3). The majority 667/853

(78.2%) of the respondents were males, 783/853 (91.8%) of the household heads were males, and 618/853 (72.4%) resided in rural areas. The average household size was five people. The median monthly income was UGX 150,000 (USD 40.5). Only 158/853 (18.5%) were in the wealthiest quintile, while 349/853 (41.0%) of the households were in the second and poorest wealth quintiles (Table 2).

Table 2: Background characteristics of the respondents

Variable	Frequency (N=853)	Percentage (%)
**Sex of respondent**		
Male	667	78.2
Female	186	21.8
**Sex of the household head**		
Male	783	91.8
Female	70	8.2
**Residence**		
Rural	618	72.4
Urban	235	27.6
**Marital status**		
Married	694	81.4
Single	85	10.0
Separated	48	5.6
Widowed	26	3.0
**Age category**		
18-25	124	14.5
26-35	303	35.5
36-45	239	28.0
46-55	126	14.8
>55	61	7.2
**Education level**		
None	51	6.0
Primary	445	52.2
Secondary	280	32.8
Tertiary	77	9.0
**Religious denominations**		
Muslim	304	35.6
Protestant	276	32.4

Catholic	194	22.7
Born again	53	6.2
SDA	26	3.1
**Occupation**		
Farmer	327	38.3
Fishermen	248	29.1
Business	146	17.1
Commercial cyclists	132	15.5
**Wealth quintiles**		
Poorest (<-1.39)	172	20.2
Poor (-1.40 - -0.50)	177	20.8
Moderate (-0.51 -0.29)	163	19.1
Wealthy (0.30 -1.23)	183	21.5
Wealthiest(1.24-4.14)	158	18.5

Willingness to pay for the proposed NHIs

The majority 695/853 (81.5%) of the respondents were willing to pay for the proposed NHIs. Of those willing to pay, the median WTP was UGX 25,000 (USD 6.8) while the mean WTP was UGX 28,950 (USD 7.8) per person, inclusive of four dependents per year. Only 3.8% protested contributing to the scheme, and 126/853 (14.7%) had zero WTP because they believed they were too poor to pay for national health insurance (Table 3).

Qualitatively, most of the FGD participants were also willing to make contributions for health insurance. However, others resented making contributions, as noted below:

> *"Sincerely, the government is meant to provide all the medical services. So, there is no way that you can lie to us and say that the government cannot provide those services..."* **(FGD male-fishermen-Mayuge)**

Several FGD participants were not willing to pay because of poverty, while others criticized the government's misuse of taxes collected from the taxpayer on salaries for officials rather than on health services, as noted in the quotes below.

> *"The fact is that poverty is the biggest hindrance. Because at times you can fall sick, yet you do not have money, and you resort to borrowing from colleagues who may not even be able to help you out because they also do not have. So generally, I disagree with paying for health insurance."* **(FGD-Business Women, Mayuge District)**

> *"I am not willing to pay because we are always charged a lot of tax to register our businesses. That is all tax. Where does that tax go? Let them reduce the salary of Members of Parliament so that part of it is utilized to buy medicine. Members of Parliament have no importance in our area."* (**FFGD-Male-fishermen-Mayuge district)**

Willingness to pay with copayments:

A copayment is a cost-sharing arrangement where one covers a portion of the costs out of pocket each time one visits the health facility. Less than half, 309/695 (44.5%) of the respondents, were willing to co-pay. The mean co-payment amount was UGX 2,650 (USD 0.7), with a median of UGX 2000 (USD 0.5), each time someone accessed services. The average WTP with co-payment was UGX 23,000 (USD.6.2) and the median WTP was UGX 20,000 (USD.5.4) (Table 3).

Table 3: Willingness to pay for the proposed NHI annually

**WTP**	**Frequency (%)**	**Mean**	**Median**
Protests (WTP=0)	32 (3.8)	0	0
WTP=0 (zero WTP)	126 (14.7)	0	0
WTP>0	695(81.5)	**28,950**	**25,000**

Total	853 (100.0)	23,600	20,000
**Co-payment scenario**			
Amount willing to co-pay	309/695(44.5)	**2,650**	**2,000**
WTP with co payment	309/695(44.5)	23,000	20,000

Notes: *Protests-those protested attaching value to HI (WTP=0 but it has no economic sense).*

WTP=0 means Zero WTP. WTP>0 that it is positive -WTP is above zero.

Qualitatively, most of the FGD participants were not in support of co-payments, as expressed by one of the FGD participants.

> *"I also do not support the idea that we pay money each time we visit the health facility, especially when we are paying every year. This will make people unable to access the services, and hence the problem of access to services will not have been solved. People are already poor, and that will mean people will continue to sell their property to go to health facilities."* (FGD Male Farmer)

Preferred mode of payment

Most (54.1%) respondents preferred to pay annually, while 21.0% preferred to pay per harvesting season. Only 13.1% wanted to pay according to their monthly income, with the majority (63.1%) preferring to pay 1 percent of their monthly income, followed by those who were willing to pay 5%.

Perceived ability to pay for the NHIs

More than half 633/853 (74.2%) of the respondents believed that they were able to pay for health insurance, with a mean of UGX 23,000 (USD.6.2) and median of UGX 20,000 (USD.5.4) per person per year.

Regarding the perceived ability to pay, most of the FGD participants noted that most of the people are poor and most likely may not be able to make periodic subscriptions, especially if the money is raised more than what people earn. This was attested to, as indicated below:

> *"Sincerely speaking, some people cannot afford to pay money; for instance, you may find a very old person who is no longer earning anything, yet they also need health care."* **(FGD-commercial cyclist-Iganga district)**

Another FGD participant, who was a business lady, noted that

> *"I support the idea. But not very much because of the issue of affordability. Not everyone can afford to pay and note that we earn differently. Because some of us play the role of mother and father in the family, there are a lot of requirements to provide."* **(FGD Businesswomen, Iganga)**

Furthermore, most of the participants noted that setting premiums should be based on people's income and ability to pay, as noted below:

> *"Since everyone earns differently, the charges should depend on how much someone earns. I could be able to afford UGX 50,000, but my colleague cannot at all since people in the village are used to getting free medicine. When some people are asked to pay just 500shs for drugs, they will consider it unworthy because they know that drugs/medication are freely given. So, if possible, we charge according to people's income."* **(FGD: businesswomen, Iganga district)**.

Determinants for level of WTP for the proposed NHIs

At bi-variable analysis, wealth quintiles, savings group membership, having a family member with chronic illness, use of traditional medicine, and hearing about health insurance were statistically significant for willingness to pay for health insurance. The odds of being willing to pay when in the fourth wealth quintile were 2.88, compared to the odds of being willing to pay among those in the poorest category (COR: 2.88, 95% CI: 1.60–5.18, P<0.001). Not being in the saving group reduced the chances of being willing to pay for health insurance by 42% compared to those who had saving group memberships (COR: 0.58, 95% CI: 0.33-0.69, P<0.001) (Table 4).

After adjusting for any confounding factors, occupation, wealth, saving group membership, use of traditional medicine, and having heard about health insurance were significantly associated with the level of WTP for the proposed NHIs.

The odds of fishermen's WTP for NHIs were 1.70 compared to those of farmers after adjusting for other factors (AOR: 1.70, 95% CI: 1.04–2.79, P = 0.035). Respondents in the fourth wealth quintile were 2.98 times more likely to pay for NHIs compared to those in the poorest wealth quintile (AOR: 2.98, 95% CI: 1.56–5.65). Respondents who were not in any saving group were 0.51 times less likely to pay for the proposed NHIs compared to those with saving group membership (AOR: 0.51, 95% CI: 0.34-0.76, P<0.001), and individuals who had never heard about HI were 50% less willing to pay for the proposed NHIs compared to those who had ever heard about health insurance (AOR: 0.50, 95% CI: 0.23-0.86, P = 0.032) (Table 4).

Table 1: Multi variable analysis of factors associated with WTP for NHI scheme.

**Variable**	**Level of Willingness to pay**		**COR (95%CI)**	**AOR (95%CI)**
	**No (n=158)**	**Yes (n=695)**		
**Sex of respondent**				

Male	127(80.4)	540(77.7)	1.0	
Female	31(19.6)	155(22.3)	1.18(0.76-1.81)	1.30(0.80-2.11)
**Residence**				
Rural	110(69.6)	508(73.1)	1.0	
Urban	48(30.4)	187(26.9)	0.84(0.58-1.23)	1.09(0.52-2.26)
**Occupation**				
Farmer	60(38.0)	267(38.4)	1.0	
Business	32(20.2)	114(15.4)	0.80(0.49-1.30)	0.59(0.26-1.35)
Commercial cyclist	25(15.8)	107(15.4)	0.96(0.57-1.61)	1.04(0.50-2.18)
Fisherfolk	41(26.0)	207(29.8)	1.13(0.73-1.76)	1.70(1.04-2.79) *
**Wealth quintiles**				
Poorest	43(27.2)	129(18.6)	1.0	
Second poorest	32(20.3)	145(20.9)	1.51(0.90-2.53)	1.83(1.05-3.19) *
Middle	35(22.1)	128(18.4)	1.22(0.73-2.03)	1.23(0.70-2.14)
Fourth	19(12.0)	164(23.6)	2.88(1.60-5.18)	2.98(1.58-5.65) *
Wealthiest	29(18.4)	129(18.6)	1.48(0.87-2.52)	1.53(0.81-2.88)
**In saving group**				
Yes	49(31.0)	337(48.5)	1.0	
No	109(69.0)	358(51.5)	0.48(0.33-0.69)	0.51(0.34-0.76) **
**Family member with chronic illness**				
Yes	36(22.7)	241(34.7)	1.0	
No	122(77.2)	576(67.5)	0.56(0.37-0.83)	0.73(0.46-1.14)
**Ability to meet health care**				
Difficult	112(70.9)	571(82.2)	1.0	
Easy	46(29.1)	124(17.8)	0.53(0.36-0.78)	0.68(0.43-1.05)
**Use Traditional Medicine**				
No	59(38.8)	345(51.4)	1.0	
Yes	93(61.2)	326(48.6)	0.60(0.42-0.86)	0.71(0.48-0.92) *
**Ever heard about NHI**				
**Yes**	9(5.7)	83(11.9)	1.0	

**No**	149(94.3)	612(88.1)	0.45(0.22-0.91)	0.50(0.23-0.86) *

Note: ** $p<0.001$, *$P<0.05$

Discussion

This study assessed the WTP and perceived ability to pay for the proposed NHIs and its determinants among informal sector workers in Iganga and Mayuge districts, Uganda. The study found that most informal sector workers were both willing and believed they were able to pay for the proposed NHI. WTP was significantly associated with occupation, wealth, awareness, and membership in saving groups.

In this study, most of the informal sector workers were willing to make contributions to the proposed NHIs. This could be perceived as support for the proposed NHIs among the informal sector workers. Our study findings are consistent with similar studies conducted in in Nepal (71%) and in South Sudan (68%) among the informal sector (13, 24). Similarly, a systematic review and meta-analysis of WTP found that the pooled WTP for NHI in Africa and Asia was 71.0% (25). It is also consistent with studies conducted in Togo (92%) (26) and 93% in Sierra Leone (27). The high level of WTP contributes to growing evidence showing the potential of informal sector workers participating in NHIs despite their nature of having unpredictable incomes and always poor (28).

This study found a median WTP of USD 6.8 and a mean WTP of USD 7.8 per person per year, which includes coverage for four dependents. This was slightly higher than the starting bid of USD 5.4 per year, thus the informal sector workers were willing to contribute more than the initially hypothesized amount. However, the WTP was lower than the 5.5 USD per person per month in Iran, 6.6 USD per person per month in Namibia, and 3 USD in Kenya per month (18, 24,

29). These discrepancies in WTP may reflect variations in socio-economic status and differing perceptions of health insurance value among study populations. As Uganda deliberates (as of 2025) the NHIs bill, it is important to recognize the relatively low WTP among informal sector workers compared to other contexts. This underscores the need to design affordable premium structures tailored to the financial realities of this group. The informal sector, often characterized by irregular and unpredictable incomes, may struggle to afford fixed, high premiums. In Ethiopia, making premium systems more flexible eased payments (30).

In this study, membership in local savings groups was associated with a higher likelihood of WTP for the proposed NHIs. In Asian countries, saving groups were reported to be key in initiating community-based health insurance schemes (31). Savings groups provide members with a secure place to save money, generate a pool of funds, and offer loans to members in times of shock and also enable the community to meet the premiums (31). Given their widespread presence in Uganda and community trust (32), these groups present an opportunity to be leveraged on to mobilize the informal sector to save and contribute to the proposed NHIs.

Participants belonging to the highest wealth category were more likely to pay for the proposed national health insurance scheme. This is in agreement with studies conducted in Nigeria, Ethiopia, and Ghana, which have reported that socio-economic factors such as household income and wealth status have a bearing on the ease or difficulty of enrolling in a given scheme, with high premiums deterring enrollment in schemes (33-35). Similar studies conducted in Sierra Leone and Iran showed that the WTP for the HI scheme depended on the monthly income of the respondent (27, 29) . In Nepal, belonging to a lower wealth quartile was associated with a lower willingness to pay for National Health Insurance Scheme (36). These findings underscore that

WTP for NHIs is closely tied to household economic status. Thus, the WTP for NHIs unavoidably depends on the wealth and incomes of the individuals. Efforts to improve household income levels will be essential to ensure broad participation in the proposed NHIs.

The use of traditional medicine was negatively associated with participation in the health insurance scheme. This may be due to a perceived lack of need for formal health services among those who rely on traditional medicine. Studies have shown that the presence and use of alternative medicine and other forms of healthcare, such as herbalists, negatively affects participation in a health insurance scheme, as NHIs are typically designed around access to and utilization of modern medical services (37). Studies conducted in Cameroon, Burkina Faso, Nigeria, and India have reported that those who use modern medicine are more willing to pay for health insurance than those who use traditional medicine (38-40). In Uganda's pluralistic health system, the ministry of health and other mandated regulatory bodies need to institute measures that not only regulate the alternative sector but also promote the utilization of modern healthcare services through education, trust-building, and service quality improvements.

The study noted that those who had never heard about NHIs were 50% less likely to pay compared to those who had prior information about health insurance. The level of knowledge on how health insurance operates was extremely low, and statistical tests of significance could not be used in this study. This was similar to studies conducted in other Africa and Asia where the knowledge was reported to be low (25). A systematic review and meta-analysis of factors influencing WTP for voluntary contributary health insurance schemes in LMICs revealed that knowledge and understanding of the functioning of the scheme positively influence the WTP and participation in the scheme (41, 42).

Implications for policy and practice

This study offers valuable insights for the ongoing design of Uganda's NHIs, especially regarding the premiums and including the informal sector. While the WTP among informal sector workers is promising, the modest contribution amounts suggest that affordability must be central to scheme design. The MoH should adopt income-sensitive premiums and flexible payment schedules, aligned with seasonal income flows such as post-harvest periods for agricultural workers. The MoH should design targeted subsidies for the poorest households to avoid exacerbating existing health inequities.

The existing local savings groups offer an opportunity to leverage on to facilitate premium collection, raise awareness, and foster a sense of local ownership. The MoH and partners should map these groups, build their capacity and support them, as a steppingstone to roll out NHIs.

Low awareness and understanding of health insurance among the informal sector pose a significant barrier to participation in NHIs. The MoH, in collaboration with district health teams, should invest in sustained awareness campaigns using trusted channels such as local radio, community meetings, and influential local leaders. Messaging should demystify the concept of health insurance, explain how contributions are used, and highlight the benefits of enrollment. The government should also foster trust among the population through improving quality of care and accountability mechanisms.

Finally, further research should assess the actual ability to pay among informal sector workers and explore how broader social health protection mechanisms can be extended to this group effectively.

Study limitations: As with most contingent valuation studies, the elicitation technique is always subject to bias, and the assignment of the first bid or amount is also prone to bias. However, this was triangulated with open-ended questions and qualitative data. The low level of knowledge about health insurance among respondents was another limitation in attaching value to the proposed scheme. This was addressed by educating the respondents about health insurance before eliciting their willingness to pay. Despite these limitations, the study provides valuable insights into the WTP for the proposed NHIs among informal sector workers, who are often excluded from social protection programs.

Conclusions

This study indicated that the informal sector workers are willing to make contributions to the proposed NHIs. However, the premium structure should be designed with careful consideration of the ability of the informal sector to pay, given the high levels of poverty and unpredictable incomes. The study also highlights that occupation, wealth status, use of traditional medicine, prior knowledge of health insurance and trust influence WTP for NHIs among the informal sector workers. Creating awareness and fostering trust is critical to the success of the proposed NHIs.

Declarations

Consent for publication

Not Applicable

Availability of data and materials

The dataset analyzed for this study has been uploaded as a supplementary file. The columns represents with the first row as the variable names.

Competing interests

The authors declare that they have no competing interests

Funding

The author(s) received no specific funding for this work.

Authors' contributions

NN, SNK, AK conceived the study, NN collected, analyzed, and wrote the first draft of the manuscript. SNK and AK provided reviews on all drafts of manuscript. All the authors read and approved the final manuscript.

Acknowledgements

We thank the District Health Officers of Mayuge and Iganga districts for their cooperation, mobilization and ensuring that data collection went on smoothly. We also extend our sincere appreciation to the research assistants and all respondents in Iganga and Mayuge for sparing their time to contribute to this piece of work.

References

1. WHO. Universal health coverage (UHC): World Health Organization 2023 [Available from: https://www.who.int/news-room/fact-sheets/detail/universal-health-coverage-(uhc).

2. WHO. Health Systems Financing: The Path to Universal Coverage. Geneva: World Health Organization 2010.

3. World Bank. Financing health in low-income countries. Washington DC: World Bank Group; 2010.

4. WHO. Noncommunicable diseases: World Health Organization 2024 [Available from: https://www.who.int/news-room/fact-sheets/detail/noncommunicable-diseases.

5. MoH. National Health Accounts 2018/19-2020/21 Report. 2021.

6. Tashobya CK, Ssengooba F, Cruz VO, Yates R, Murindwa G, McPake B. Health systems reforms in Uganda: processes and outputs. Christine Kirunga Tashobya FS and, Valeria Oliveira Cruz, editors. 2006.

7. UBOS. The National Population and Housing Census 2014 – Main Report. Kampala, Uganda: Uganda Bureau of Statistics; 2016.

8. Twaweza. Health Check: Ugandans' experiences and opinions on health services Uganda: Twaweza; 2019 [Available from: https://www.twaweza.org/go/szw-ug-health-check.

9. Gottret P, Schieber G. Health financing revisited: a practitioner's guide: The World Bank; 2006.

10. Xu K, Evans DB, Carrin G, Aguilar-Rivera AM, Musgrove P, Evans T. Protecting households from catastrophic health spending. Health affairs. 2007;26(4):972-83.

11. De Allegri M, Sanon M, Bridges J, Sauerborn R. Understanding consumers’ preferences and decision to enrol in community-based health insurance in rural West Africa. Health policy. 2006;76(1):58-71.

12. Kyomugisha E, Buregyeya E, Ekirapa E, Mugisha J, Bazeyo W. Strategies for sustainability and equity of prepayment health schemes in Uganda. African health sciences. 2009;9(2).

13. Basaza R, Alier PK, Kirabira P, Ogubi D, Lako RLL. Willingness to pay for National Health Insurance Fund among public servants in Juba City, South Sudan: a contingent evaluation. International journal for equity in health. 2017;16(1):158.

14. Nguyen H, Knowles J. Demand for voluntary health insurance in developing countries: the case of Vietnam's school-age children and adolescent student health insurance program. Social Science & Medicine. 2010;71(12):2074-82.

15. Acharya A, Vellakkal S, Taylor F, Masset E, Satija A, Burke M, et al. Impact of National Health Insurance for the Poor and the Informal sector in Low-and Middle-Income Countries. London: EPPI-Centre, Social Science Research Unit, Institute of Education, University of London; 2012.

16. Basaza R, Criel B, Van der Stuyft P. Community health insurance in Uganda: why does enrolment remain low? A view from beneath. Health policy. 2008;87(2):172-84.

17. Jowett M, Kutzin J, Organization WH. Raising revenues for health in support of UHC: strategic issues for policy makers. World Health Organization; 2015.

18. Gustafsson-Wright E, Asfaw A, van der Gaag J. Willingness to pay for health insurance: An analysis of the potential market for new low-cost health insurance products in Namibia. Social science & medicine. 2009;69(9):1351-9.

19. Russell S, Fox-Rushby J, Arhina D. Willingness and ability to pay for health care: a selection of methods and issues. Health Policy and Planning. 1995;10(1):94-101.

20. Mitchell RC, Carson RT. Using surveys to value public goods: the contingent valuation method: Rff Press; 2013.

21. Kish L. Survey sampling. Inc., New York.: John Wiley and Sons; 1965.

22. Whittington D, Smith VK, Okorafor A, Okore A, Liu JL, McPhail A. Giving respondents time to think in contingent valuation studies: a developing country application. Journal of Environmental Economics and Management. 1992;22(3):205-25.

23. Dong H, Kouyate B, Cairns J, Sauerborn R. A comparison of the reliability of the take-it-or-leave-it and the bidding game approaches to estimating willingness-to-pay in a rural population in West Africa. Social Science & Medicine. 2003;56(10):2181-9.

24. Barasa EW, Mwaura N, Rogo K, Andrawes L. Extending voluntary health insurance to the informal sector: experiences and expectations of the informal sector in Kenya. Wellcome open research. 2017;2.

25. Bayked EM, Assfaw AK, Toleha HN, Zewdie S, Biset G, Ibirongbe DO, et al. Willingness to pay for National Health Insurance Services and Associated Factors in Africa and Asia: a systematic review and meta-analysis. Frontiers in Public Health. 2024;12:1390937.

26. Djahini-Afawoubo DM, Atake E-H. Extension of mandatory health insurance to informal sector workers in Togo. Health economics review. 2018;8:1-13.

27. Jofre-Bonet M, Kamara J. Willingness to pay for health insurance in the informal sector of Sierra Leone. PloS one. 2018;13(5):e0189915.

28. ILO. Women and men in the informal economy: A statistical picture. Geneva ILO; 2018.

29. Nosratnejad S, Rashidian A, Mehrara M, Sari AA, Mahdavi G, Moeini M. Willingness to pay for social health Insurance in Iran. Global journal of health science. 2014;6(5):154.

30. Habte A, Tamene A, Ejajo T, Dessu S, Endale F, Gizachew A, et al. Towards universal health coverage: The level and determinants of enrollment in the Community-Based Health Insurance (CBHI) scheme in Ethiopia: A systematic review and meta-analysis. PloS one. 2022;17(8):e0272959.

31. Shaikh BT, Noorani Q, Abbas S. Community based saving groups: an innovative approach to overcome the financial and social barriers in health care seeking by the women in the rural remote communities of Pakistan. Archives of Public Health. 2017;75(1):57.

32. Mutebi A, Muhumuza Kananura R, Ekirapa-Kiracho E, Bua J, Namusoke Kiwanuka S, Nammazi G, et al. Characteristics of community savings groups in rural Eastern Uganda: opportunities for improving access to maternal health services. Global health action. 2017;10(sup4):1347363.

33. Kotoh AM, Aryeetey GC, Van der Geest S. Factors that influence enrolment and retention in Ghana'National Health Insurance Scheme. International journal of health policy and management. 2018;7(5):443.

34. Odeyemi IA. Community-based health insurance programmes and the national health insurance scheme of Nigeria: challenges to uptake and integration. International journal for equity in health. 2014;13(1):20.

35. Mebratie AD, Sparrow R, Yilma Z, Alemu G, Bedi AS. Enrollment in Ethiopia's community-based health insurance scheme. World Development. 2015;74:58-76.

36. Khatiwada B, Ghimire S, N S. Willingness to pay for health insurance in mangalbare village development committee of illam district. MOJ Public Health. 2017;5(2):43-6.

37. Sarkodie AO. Effect of the National Health Insurance Scheme on healthcare utilization and out-of-pocket payment: evidence from GLSS 7. Humanities and Social Sciences Communications. 2021;8(1):1-10.

38. Mathiyazaghan K. Willingness to pay for rural health insurance through community participation in India. The International journal of health planning and management. 1998;13(1):47-67.

39. Donfouet HPP, Mahieu P-A, Malin E. Using respondents' uncertainty scores to mitigate hypothetical bias in community-based health insurance studies. The European Journal of Health Economics. 2013;14(2):277-85.

40. Oriakhi H, Onemolease E. Determinants of rural household's willingness to participate in community based health insurance scheme in Edo State, Nigeria. Studies on ethno-medicine. 2012;6(2):95-102.

41. Adebayo EF, Uthman OA, Wiysonge CS, Stern EA, Lamont KT, Ataguba JE. A systematic review of factors that affect uptake of community-based health insurance in low-income and middle-income countries. BMC health services research. 2015;15(1):543.

42. Dror DM, Hossain SS, Majumdar A, Koehlmoos TLP, John D, Panda PK. What factors affect voluntary uptake of community-based health insurance schemes in low-and middle-income countries? A systematic review and meta-analysis. PLoS One. 2016;11(8):e0160479.

Click here to access/download
Supporting Information
Dataset-De identified 13 May 2025.xlsx

Data set

Click here to access/download
Supporting Information
Dataset-De identified 8th May 2025.xlsx

Click here to access/download
Other
Namuhani_IRB_Approval_letter_April_2019.pdf

Willingness and Perceived ability to pay for Uganda's Proposed National Health Insurance Scheme among Informal Sector workers in Iganga and Mayuge districts, Uganda: A Contingent Valuation Method.

Noel Namuhani[1*], Angela Kisakye[1, 2] and Suzanne N Kiwanuka[1]

Institutional affiliations

[1]Makerere University School of public health, Department of Health policy planning and management, Uganda

[2]African Field Epidemiology Network, Lugogo House, Plot 42, Lugogo By-Pass

*Corresponding Author

Email: nnamuhani@musph.ac.ug

Abstract

Background: Access to health care remains a challenge, especially among the informal sector workers in most low-income countries, due to high out-of-pocket ~~(OOP)~~ expenditures (OOPE), with Uganda spending over 28.0% out of pocket on health care. In response, Uganda has proposed a national health insurance scheme (NHI). However, the willingness and ability to pay for the proposed NHI scheme within the informal sector have not yet been explored in Uganda. This study assessed the willingness and perceived ability to pay for the proposed NHI scheme and its determinants among the informal sector workers in Iganga and Mayuge districts, Uganda.

Methodology: A cross-sectional study was conducted in Iganga and Mayuge districts between 10/04/2019 and 23/05/2019. A contingent valuation method using the bidding game technique was used to elicit the willingness to pay (WTP). A total of 853 informal sector workers, including farmers, commercial motorists, fishermen, and traders, were randomly sampled. Seven focus group discussions (FGD) were also conducted. Logistic regression was done to identify the determinants of willingness to pay for the proposed NHI scheme. Qualitative data was analyzed thematically.

Results: The majority 695/853, (81.5%) of the respondents were willing to pay for NHI; the median WTP was UGX 25,000 (USD 6.8) annually; and 633/853, (74.2%) of the respondents believed that they were able to pay for the health insurance. Willingness to Pay was significantly associated with being a fisher folk (AOR: 1.70 95%CI: 1.04-2.79, P = 0.035), being in the fourth wealth quintile (AOR: 2.98, 95% CI: 1.56–5.65), not hearing about health insurance (AOR: 0.50 95%CI: 0.23-0.86, P = 0.032), and not having saving group membership (AOR: 0.51, 95%CI: 0.34-

0.76, P<0.001). Most of the FGD participants were willing to pay for the proposed scheme; however, some of the participants doubted their ability to pay for the scheme given their high poverty levels and their unstable income.

Conclusion: The willingness to pay for health insurance in the informal sector is high. Therefore, it is viable for the government to extend NHI to the informal sector. However, awareness building and due consideration of high poverty levels in setting appropriate premiums should be a priority.

Key words: National Health insurance scheme, Willingness to pay, Ability to pay, informal sector, Contingent Valuation method.

Author Summary

Many Ugandans especially those in the informal sector struggle to access healthcare due to high out-of-pocket healthcare costs. To solve this, Uganda plans to establish a national health insurance scheme. However, the extent to which the informal sector workers who constitute the largest portion of the labor force are willing and able to pay is not well understood. In this paper therefore, we sought to explore the extent to which the informal sector workers are willing and able to pay for the proposed national health insurance scheme.

To answer our objective, we interviewed 853 workers who participate in farming, fishing, traders and commercial cyclists in Iganga and Mayuge districts. We found that most respondents (82%) were willing to pay for the proposed health insurance, an average annual amount of UGX 25,000

(about USD 6.80). Over 74% believed they could afford to pay for the proposed scheme, while others expressed concerns about their capacity to pay due to poverty and unstable incomes.

Our study findings show that extending health insurance scheme to informal sector workers is feasible, however government need to factor in affordability when setting premiums given the high poverty levels.

Formatted: Font: Not Bold

Introduction

Globally, there is a growing commitment to achieving Universal Health Coverage (UHC), which is an important element for achieving the Sustainable Development Goals (SDGs) (1). UHC aims to address the poor access to quality and affordable health services, which many African countries are grappling with~~Access to health care remains a big challenge, especially among the informal sector in most low-income countries~~ (2, 3), mainly due to high out-of-pocket expenditures(OOPe) ~~expenditures~~ (4). In 2021, it was estimated that about 4.5 billion people (more than half) in the world were not fully covered by essential health services, while about 2 billion (a quarter) of the world's population were facing financial hardship due to out-of-pocket health spending (1). This put about 150 million people at risk of catastrophic healthcare costs annually, driving over 100 million people into poverty worldwide (4). According to the World Bank, direct modes of payment for health care services constitute 40% of health care expenditure in developing countries (5).

Formatted: Font: (Default) +Body (Calibri)

Formatted: Space After: 8 pt

Formatted: Font: (Default) +Body (Calibri)

Formatted: Font: (Default) +Body (Calibri)

Formatted: Font: (Default) +Body (Calibri)

Formatted: Font: (Default) +Body (Calibri)

Formatted: Font: (Asian) Chinese (PRC)

In Uganda, although the national health accounts reported a reduction in the out-of-pocket expenditure on health as a share of current health expenditure (CHE) from 38.6% in the financial year 2018/2019 to 27.4% in the financial year 2020/2021, the OOPE remains significantly high. This is compounded by the complex health financing landscape, where the general government

health expenditure per capita allocation is very low estimated at 11.6 $, heavy reliance on donor funding accounting for 50.3% of the total health expenditure which has significantly reduced over the years. (6). Uganda abolished user fees in public health facilities, however, a~~A~~ccessing healthcare is still associated with several expenses, including transport, drugs, diagnostics, and consultancy fees, among other expenses related to personnel and capital costs (7).

Globally, more than 60% of the employment occurs in the informal sector and i~~I~~n Uganda, over 80% of the labor force belongs to the informal sector (8). These include peasant farmers, commercial cyclists, fishermen, and those employed in unregistered or small-scale enterprises. It is estimated that 59% of the people in the informal sector in Eastern Uganda cannot afford to access quality health care due to high poverty levels and OOP for health care (9).

Formatted: Font: 11 pt

Formatted: Font: 11 pt

Globally, the implementation of health insurance schemes is one of the major responses to addressing out-of-pocket payments for health care (4, 10) in low and middle-income countries. Health insurance has the ability to reduce financial barriers to health care access and protect individuals and families against the risk of unpredictable health care expenditures, hence aiding the attainment of UHC (11). This has improved access to healthcare, especially in developed countries.

~~High~~High coverage and enrollment in the health insurance scheme are critical for the success and sustainability of the scheme, given that premiums constitute the proposed main source of funding for the scheme. However, enrolment in health insurance schemes remains low, ranging from 2% to 40% in most African countries (12-14). The poor enrolment rates in the different countries have been attributed to a number of factors, including low incomes, large household

sizes, long distances to health facilities, limited awareness, poor quality of health care services, inappropriate benefit packages, a lack of trust in the systems, and high illiteracy levels, especially in the informal sector (15-17). The informal sector workers are sometimes of low socioeconomic status compared to the formal sector workers, hence having a lower ability to pay for health insurance (17). Furthermore, the informal sector is not well organized; they have unpredictable incomes, making it difficult to enroll and register them into the NHI scheme and collect regular contributions from them (14, 18).

Uganda is in the process of developing a NHI scheme to address the high OOP and access to services. However, given the high poverty levels among Ugandans and more so in the informal sector, the policy question of how much money people are willing to spend and their ability to pay remains unanswered. Eastern Uganda is the poorest region in Uganda, with 35.7% of most households living below the poverty line (8). The willingness to pay for the proposed NHI scheme, especially within the informal sector, which makes up 80% of Uganda's population, is critical to the achievement of equity in access to care and the core risk pooling function of a health insurance scheme. This would translate into high enrollment rates, which foster the financial sustainability of the schemes (11).

The national health insurance bill, 2019, proposes that the scheme will be mandatory for all Ugandans, covering both the formal sector and informal sector workers and each person is to pay a premium that will be determined by the board, to access the scheme. The main source of funding for the proposed scheme are premium contributions and government contributions for the indigents. The services will be obtained both in public and accredited private health facilities.

The bill was passed by parliament in 2021 but was not assented to by the president due to disagreements among stakeholders who were not consulted. As of 2024, the bill is being discussed by the Ministry of Health and stakeholders before being re-tabled in the Parliament of Uganda.

There is a paucity of information on the willingness and ability to pay for the proposed NHI, especially within the informal sector. Understanding the level of and determinants for willingness to enroll and pay for the proposed NHI plays an important role in designing strategies for improving enrollment rates into the scheme. Therefore, this study aimed at documenting the willingness to pay and the perceived ability to pay for the proposed NHI and factors that influence willingness to pay for the proposed NHI scheme among the informal sector workers. This study's findings will be used by the MOH in determining affordable premiums, ~~and how they should be paid.~~designing strategies for ensuring maximum participation especially among the informal sector workers. The study findings will also inform the National health insurance scheme bill under discussion.

Materials and Methods

Study design: This was a cross-sectional study design that involved mixed methods of data collection. The quantitative and qualitative methods were conducted concurrently. The study was conducted from 10/04/2019 to 23/05/2019. This study adopted a contingent valuation method (CVM) using the bidding game technique to directly elicit or measure willingness to pay for a hypothetical (not yet on the market) health insurance package (19). This approach has been used because it has been proven to be an effective way of assessing willingness to pay with

minimal bias for goods that are not presently available on the market. The CVM creates a hypothetical marketplace in which no actual transactions are made; hence, this approach has been successfully used for commodities that are not exchanged in regular markets. This approach makes the assumption that people have had no previous experience buying the health service that is going to be put on the market and instead asks people their willingness to pay on the basis of their expectations and the benefits attached to the service (20). In the absence of markets for public goods, this method presents consumers with hypothetical markets in which they have the (hypothetical) opportunity to buy the good, health insurance in this case ~~d in question~~. Since the elicited WTP values are contingent upon the particular hypothetical market described to the respondent, this approach is called the contingent valuation method (21).

Study setting: The study was conducted in the Iganga and Mayuge districts of eastern Uganda. Iganga and Mayuge districts were selected purposively because they are the most poverty-hit districts in the country and 80% of the population lives in rural areas. They also have 80–85% of the population belonging to the informal sector (8). Iganga District is bordered by Kaliro District to the north, Bugweri District to the east, Mayuge District to the south, Jinja District to the southwest, and Luuka District to the west. With a total population of 504,197 people, 9.9% of the households are 5 km or more from the nearest health facility, and 19.9% are near a public health facility (UBOS, 2016). It is composed of 3 counties, 8 sub counties, 66 parishes, and 395 villages. The major economic activity is farming. Mayuge district is located in south-eastern Uganda with a population of 473,2394 and a growth rate of 3.5%, and it has thirteen subcounties. The major economic activities are farming and fishing. Mayuge district is bordered by Lake Victoria to the south, as well as Jinja and Bugiri districts. It has a total of 33 landing sites where fishing activities

take place. Uganda has a decentralized healthcare system, having both public and private health facilities. The MoH at the center is the regulator and it will regulate the operations of the NHIs. At regional level, there are regional referral hospitals, and district level, where policies are implemented. At the district level exist district hospitals, health center IVs at county level, health center IIIs at the subcounty level, heakth center IIs at parish level and village health teams at village level.

Study Population: The study targeted the four major categories of informal sector workers: 1) farmers; 2) fishermen; 3) commercial cyclists; and 4) the business community (traders and market vendors) in the study area. The informal sector was chosen because they make up the largest (80%) portion of the population, without which the sustainability of the scheme will be questionable.

Sample Size: The sample size was calculated using the Kish Leslie formula (22), considering a 95% confidence interval, a 50% expected level of WTP, a 5% level of precision, a design effect of 2, and a 10% non-response rate. This gave a total sample size of 853 informal sector workers. Four focus group discussions were conducted with women (four FGDs), and three FGDs were conducted with men (in the informal sector). This number depends on the level of information saturation. This was determined by doing preliminary analysis while still in the field.

Sampling Procedure: Stratified random sampling was used in this study. For Iganga district, the informal sector workers were divided into three major categories (strata): farmers, commercial cyclists, traders/market vendors. In Mayuge district, the informal sector workers were divided into four categories: fishermen, farmers, commercial cyclists, and market vendors. A random

sample from each stratum was taken in a number proportional to the stratum's size when compared to the population. In Iganga district, two subcounties were randomly selected, and five villages from each subcounty were also randomly selected. A proportionate sample of 286 households from the 10 villages was selected, and the household heads were systematically selected for interviews. Seven *bodaboda* stages located along main roads and junctions were randomly selected, and 10 cyclists available at the time of the study were randomly selected to participate in the interviews. A total of 84 traders were selected systematically from the two main markets for interviews. In Mayuge district, a total of 10/33 landing sites were randomly selected, and 248 fishermen were selected from the 10 landing sites proportionate to size. Sixty-two traders were randomly selected from three main markets systematically, and 62 bodaboda cyclists were randomly selected from six stages located along main roads in Mayuge town (Table 1). Men and women from each of the categories were purposively selected for the focus group discussions. These were selected and identified with the help of local leaders based on community members who can express themselves, but also are residents and working in the informal sectors of interest.

Table 1: Sampling procedure per category of informal sector

**Iganga district (**Iganga district planning office, 2018)				
**Category**	**Farmers/peasants**	**Commercial cyclists**	**Traders/vendors**	**Fisherfolk**
Proportion in the district	0.65	0.16	0.19	
Sample size	(0.65*440) =286	(0.16*440) =70	(0.19*440) =84	-
Selection procedure	2 sub counties (SC) and 5 villages from each SC were randomly selected.	7 *Boda boda* stages in the town were randomly selected, cyclists on stage	Traders were systematically selected for the interviews two main markets	-

	Households proportionate to size selected.	randomly selected to be interviewed		
Mayuge district				
**Category**	**Farmers/peasants**	**Commercial cyclists**	**Traders/vendors**	**Fisherfolk**
Proportion in the district	0.1	0.15	0.15	0.6
Sample size	(0.1*413) =41	(0.15*413) =62	(0.15*413) =62	(0.6*413) =248
Selection procedure	One sub county and two villages were randomly selected, the number of households were determined proportionate to size.	6 *Boda boda* stages in town were randomly selected, later cyclists on stage at that time were randomly selected to be interviewed	Traders were systematically selected from 3 main markets	10/33 landing sites were randomly selected. Fishermen at each site were randomly selected, proportionate to size
**Total samples**	**327**	**132**	**146**	**248**

Data collection methods and tools

The willingness to pay was assessed using a contingent valuation method using the "bidding game" technique. The bidding game was used to determine the maximum premium that a respondent is willing to pay for the proposed NHI plan. A bidding game technique was chosen because of its ease of implementation and its minimal biases. In addition, many studies have found the bidding game to be very reliable (23, 24). The respondent was asked if he or she was willing to pay the starting bid. If the respondent agreed, the interviewer would raise the bid by 10% of the first bid and again ask if the respondent was willing to pay the new bid. The interviewer continued to present the different bids until the respondent expressed an unwillingness to pay. On the other hand, if the respondent expressed unwillingness to pay the

starting bid, the interviewer lowered the bid by 10% and repeated the query. This was continued until a bid was reached where the respondent was willing to pay. The starting bid was 20,000 UGX (5.4 USD), since this was the average premium for beneficiaries in the existing community-based health insurance schemes in Luweero, Western Uganda, and Jinja. The proportion of those willing to pay, the average, and the median amount of money people were willing to pay were computed. The perceived ability to pay was measured by asking respondents whether they were able to pay premiums for health insurance per year and how much they could afford without foregoing other basic needs.

A semi-structured questionnaire was used to collect quantitative data such as sociodemographics, WTP, perceived ability to pay, and associated factors from informal sector workers. The data collection tools were translated to the local language and then back translated to English to check whether the translated questions still held informational validity. The principal investigator recruited and trained research assistants so that they were familiar with the statement of the problem, objectives of the study, sampling procedure, data collection tools, and plan for data collection. Research assistants were oriented to the bidding game technique for assessing WTP. They were also taught the meaning of health insurance and how it intends to be operationalized in the country. Research assistants were also trained on the basic interview techniques, such as asking questions in a neutral manner, not showing by words or actions what answers were being expected of the study participants, and how to record answers, especially from open-ended questions, without interpreting them. The data collection tools were pre-tested in the neighboring district, Bugweri district. This was done to test the validity and ease of application of the tools. Filled electronic forms were checked at the point of data collection for

completeness, and those found incomplete were completed before the respondent was discharged. The data were cleaned and edited by the research assistants before they submitted the complete forms to the server.

Focus group discussion (FGD) guides were also used to collect data from informal sector workers to understand the determinants of willingness to pay and their perceived ability to pay for the NHI. The FGDs were audio-taped. The FGD was composed of 6–8 participants. The discussion had a moderator and a note-taker, and the discussions lasted for an average of one hour. These were conducted in the local language, and these were audio recorded.

Data management and analysis: The quantitative data were downloaded from the server and imported into STATA 14 for cleaning and analysis. Descriptive statistics, including mean, median, standard deviations, frequencies, and percentages, were obtained. The information was presented in frequency distribution tables. Principal component analysis was done to generate wealth quintiles using the demographic health survey categorization. The wealth quintiles were generated based on nine household items, which included possession of a car, motorcycle, bicycle, radio, television, mobile phone, piece of land, owning a house, and having animals. Inferential statistics were obtained using logistic regression. This was done in two stages: 1) bivariate analysis was done to determine the potential variables associated with willingness to pay for health insurance. Crude odds ratios at a 95% confidence interval were obtained to measure the potential association. 2) A multivariate analysis was done to determine the actual factors associated with WTP. All variables that were statistically significant at bivariate analysis and the important factors known in the literature to be statistically significant, such as socio-

economic status, occupation, and potential confounders like residence, were included in the model. Variables were tested for multicollinearity before being included in the model. The model was built step-by-step while testing it using lfit until the best model was obtained. Variables that remained statistically significant at 95% confidence were considered the factors associated with WTP for health insurance. Adjusted odds ratios were obtained as measures of association.

Qualitative data was analyzed manually using a thematic-inductive approach. The recordings were transcribed verbatim and translated from the local language (Lusoga) to English. Transcripts were read by more than two independent analysts who identified codes, sub-themes, and then organized them into themes. The information was triangulated with the quantitative data findings to gain a deeper understanding.

Ethics statement

The study was approved by the Makerere University, School of Public Health Higher Degrees, Research and Ethics Committee (HDREC). At the district level, permission to conduct the study was obtained from the local leaders and the District Health Office. Permission was also sought from subcounty chiefs and local council one chairpersons. The informed written consent of each individual participant was obtained at the start of the study. Confidentiality and anonymity were maintained by the use of codes.

Results

Background Characteristics of the Respondents

Of the 853 respondents who participated in the study, 327 (38.3%) were peasant farmers, 248929.1%) were fishermen, 146 (17.1%) were business people, and 132 (15.5%) were commercial cyclists. The mean age of the respondents was 37.0 years (SD $\pm$ 11.3). The majority 667/853 (78.2%) of the respondents were males, 783/853 (91.8%) of the household heads were males, and 618/853 (72.4%) resided in rural areas. The average household size was 5 persons. The median monthly income was UGX 150,000 (USD 40.5). Only 158/853 (18.5%) were in the wealthiest quintile, while 349/853 (41.0%) of the households were in the second and poorest wealth quintiles (Table 2).

Table 2: Background Characteristics of the Respondents

**Variable**	**Frequency (N=853)**	**Percentage (%)**
**Sex of respondent**		
Male	667	78.2
Female	186	21.8
**Sex of the household head**		
Male	783	91.8
Female	70	8.2
**Residence**		
Rural	618	72.4
Urban	235	27.6
**Marital status**		
Married	694	81.4
Single	85	10.0
Separated	48	5.6
Widowed	26	3.0
**Age category**		
18-25	124	14.5
26-35	303	35.5
36-45	239	28.0
46-55	126	14.8
56 above	61	7.2
**Education level**		
None	51	6.0
Primary	445	52.2

Secondary	280	32.8
Tertiary	77	9.0
**Religion**		
Muslim	304	35.6
Protestant	276	32.4
Catholic	194	22.7
Born again	53	6.2
SDA	26	3.1
**Occupation**		
Farmer	327	38.3
Fishermen	248	29.1
Business	146	17.1
Bodaboda	132	15.5
**Wealth quintiles**		
Poorest (<-1.39)	172	20.2
Poor (-1.40 - -0.50)	177	20.8
Moderate (-0.51 -0.29)	163	19.1
Wealthy (0.30 -1.23)	183	21.5
Wealthiest(1.24-4.14)	158	18.5

Willingness to Pay for the Proposed NHI

The majority 695/853 (81.5%) of the respondents were willing to pay for the proposed NHI. Of those willing to pay, the median willingness to pay was UGX 25,000 (USD 6.8) while the mean WTP was UGX 28,950 (USD 7.8) per person, inclusive of 4 dependents per year. Only 3.8% protested contributing to the scheme, and 126/853 (14.7%) gave WTP zero because they believed they were too poor to pay for national health insurance (Table 3).

Qualitatively, most of the FGD participants were also willing to make contributions for health insurance. However, others resented making contributions, as noted below:

> *"Sincerely, the government is meant to provide all the medical services. So, there is no way that you can lie to us and say that the government can't provide those services..."* **(FGD male-fishermen-Mayuge)**

A number of FGD participants were not willing to pay because of poverty, while others criticized the government's misuse of taxes collected from the tax payer on salaries for officials rather than on health services, as noted in the quotes below.

> *"The fact is that poverty is the biggest hindrance. Because at times you can fall sick, yet you don't have money, and you resort to borrowing from colleagues who may not even be able to help you out because they also don't have. So generally, I disagree with paying for health insurance."* **(FGD-Business Women, Mayuge District)**

“I am not willing to pay because we are always charged a lot of tax to register our businesses. That is all tax. Where does that tax go? Let them reduce the salary of Members of Parliament so that part of it is utilized to buy medicine. Members of Parliament have no importance in our area." (**FFGD-Male-fishermen-Mayuge district)**

Willingness to Pay with Copayments:

A copayment is a cost-sharing arrangement where one covers a portion of the costs out of pocket each time one visits the health facility. Less than half, 309/695 (44.5%) of the respondents, were willing to co-pay. The mean co-pay amount was UGX 2,650 (USD 0.7), with a median of UGX 2000 (USD 0.5), each time someone accessed services. The average maximum amount of money that respondents were willing to pay with the copayment was UGX 23,000 (USD.6.2) and the median was UGX 20,000 (USD.5.4) (Table 3).

Table 3: Willingness to Pay for the Proposed NHI annually

**WTP**	**Frequency (%)**	**Mean**	**Median**
Protests (WTP=0)	32 (3.8)	0	0
WTP=0	126 (14.7)	0	0
WTP>0	695(81.5)	**28,950**	**25,000**
Total	853 (100.0)	23,600	20,000
**Copayment scenario**			
Amount willing to copay	309/695(44.5)	**2,650**	**2,000**
WTP with co payment	309/695(44.5)	23,000	20,000

Notes; *Protests-those protested attaching valuing to HI (WTP=0 but it has no economic sense). WTP=0 means willingness to pay is actually zero. WTP>0 that it is positive -WTP is above zero.*

Qualitatively, most of the FGD participants were not in support of copayments, as expressed by one of the FGD participants.

"I also do not support the idea that we pay money each time we visit the health facility, especially when we are paying every year. This will make people unable to access the services, and hence the problem of access to services will not have been solved. People are already poor, and that will mean people will continue to sell their property to go to health facilities." (FGD Male Farmer)

Preferred Mode of Payment

Most (54.1%) respondents preferred to pay annually, while 21.0% preferred to pay per harvesting season. Only 13.1% wanted to pay according to their monthly income, with the majority (63.1%) preferring to pay 1 percent of their monthly income, followed by those who were willing to pay 5%.

Perceived Ability to Pay for the Health Insurance Scheme

More than half 633/853 (74.2%) of the respondents believed that they were able to pay for health insurance, with a mean of UGX 23,000 (USD.6.2) and median of UGX 20,000 (USD.5.4) per person per year.

Regarding the perceived ability to pay, most of the FGD participants noted that most of the people are poor and most likely may not be able to make periodic subscriptions, especially if the money is raised more than what people earn. This was attested to, as indicated below:

"Sincerely speaking, some people cannot afford to pay money; for instance, you may find a very old person who is no longer earning anything, yet they also need health care." **(FGD-Boda-boda-Iganga district)**

Another FGD participant, who was a business lady, noted that

> *"I support the idea. But not very much because of the issue of affordability. Not everyone can afford to pay, and note that we earn differently. Because some of us play the role of mother and father in the family, there are a lot of requirements to provide."* **(FGD Business Women, Iganga)**

Furthermore, most of the participants noted that setting premiums should be based on people's income and ability to pay, as noted below:

> *"Since everyone earns differently, the charges should depend on how much someone earns. I could be in a position to afford UGX 50,000, but yet my colleague cannot at all since people in the village are used to getting free medicine. When some people are asked to pay just 500shs for drugs, they will consider it unworthy because they know that drugs/medication are freely given. So, if possible, we charge according to people's income."* **(FGD: business women, Iganga district)**.

Determinants for level of Willingness to Pay for Health Insurance Scheme

At bi-variable analysis, wealth quintiles, having savings group membership, having a family member with chronic illness, use of traditional medicine, and hearing about health insurance were statistically significant for willingness to pay for health insurance. The odds of being willing to pay when in the fourth wealth quintile were 2.88, compared to the odds of being willing to pay among those in the poorest category (COR: 2.88, 95% CI: 1.60–5.18, P<0.001). Not being in the saving group reduced the chances of being willing to pay for health insurance by 42%

compared to those who had saving group memberships (COR: 0.58, 95% CI: 0.33-0.69, P<0.001) (Table 4).

After adjusting for any confounding factors, occupation, wealth, saving group membership, use of traditional medicine, and having heard about health insurance were significantly associated with the level of willingness to pay for the proposed health insurance scheme.

The odds of fishermen's willingness to pay for health insurance were 1.70 compared to those of farmers after adjusting for other factors (AOR: 1.70, 95% CI: 1.04–2.79, P = 0.035). Respondents in the fourth wealth quintile were 2.98 times more likely to pay for health insurance compared to those in the poorest wealth quintile (AOR: 2.98, 95% CI: 1.56–5.65). Respondents who were not in any saving group were 0.51 times less likely to pay for the proposed health insurance compared to those with saving group membership (AOR: 0.51, 95% CI: 0.34-0.76, P<0.001), and individuals who had never heard about HI were 50% less willing to pay for the proposed National Health Insurance compared to those who had ever heard about health insurance (AOR: 0.50, 95% CI: 0.23-0.86, P = 0.032) (Table 4).

Table 1: Multi variable analysis of factors associated with WTP for NHI scheme.

**Variable**	**Level of Willingness to pay**		**COR (95%CI)**	**AOR (95%CI)**
	**No (n=158)**	**Yes (n=695)**		
**Sex of respondent**				
Male	127(80.4)	540(77.7)	1.0	
Female	31(19.6)	155(22.3)	1.18(0.76-1.81)	1.30(0.80-2.11)
**Residence**				
Rural	110(69.6)	508(73.1)	1.0	
Urban	48(30.4)	187(26.9)	0.84(0.58-1.23)	1.09(0.52-2.26)
**Occupation**				

Farmer	60(38.0)	267(38.4)	1.0	
Business	32(20.2)	114(15.4)	0.80(0.49-1.30)	0.59(0.26-1.35)
Boda boda	25(15.8)	107(15.4)	0.96(0.57-1.61)	1.04(0.50-2.18)
Fishermen	41(26.0)	207(29.8)	1.13(0.73-1.76)	1.70(1.04-2.79) *
**Wealth quintiles**				
Poorest	43(27.2)	129(18.6)	1.0	
Second poorest	32(20.3)	145(20.9)	1.51(0.90-2.53)	1.83(1.05-3.19) *
Middle	35(22.1)	128(18.4)	1.22(0.73-2.03)	1.23(0.70-2.14)
Fourth	19(12.0)	164(23.6)	2.88(1.60-5.18)	2.98(1.58-5.65) *
Wealthiest	29(18.4)	129(18.6)	1.48(0.87-2.52)	1.53(0.81-2.88)
**In saving group**				
Yes	49(31.0)	337(48.5)	1.0	
No	109(69.0)	358(51.5)	0.48(0.33-0.69)	0.51(0.34-0.76) **
**Family member with chronic illness**				
Yes	36(22.7)	241(34.7)	1.0	
No	122(77.2)	576(67.5)	0.56(0.37-0.83)	0.73(0.46-1.14)
**Ability to meet health care**				
Difficult	112(70.9)	571(82.2)	1.0	
Easy	46(29.1)	124(17.8)	0.53(0.36-0.78)	0.68(0.43-1.05)
**Use Traditional Medicine**				
No	59(38.8)	345(51.4)	1.0	
Yes	93(61.2)	326(48.6)	0.60(0.42-0.86)	0.71(0.48-0.92) *
**Ever heard about NHI**				
**Yes**	9(5.7)	83(11.9)	1.0	
**No**	149(94.3)	612(88.1)	0.45(0.22-0.91)	0.50(0.23-0.86) *

Note: *** p<0.001, *P<0.05*

Discussions

This study assessed the willingness and perceived ability to pay for the proposed NHI scheme and its determinants among the informal sector workers in Iganga and Mayuge districts, Uganda. The study reported that the majority of the informal sector workers were willing and believed that they were able to pay for the proposed NHI. Willingness to pay was significantly associated with occupation, wealth, awareness and saving group membership.

~~Firstly, this study is unique in its assessment of the level of WTP, perceived ability to pay, and determinants of WTP for the proposed national health insurance scheme among the informal sector workers in Uganda, who constitute 80% of the Ugandan population. These findings are pertinent if the proposed NHI is to achieve adequate coverage. Secondly, the study applied a contingent valuation approach using a bidding game technique to hypothetically elicit the informal sector's willingness to pay. The highlight of this study is the high level of willingness to pay for NHI among informal sector workers, as well as their preferred modes of engagement. These findings provide valuable insights for fine-tuning the proposed NHI.~~

The level of WTP for the proposed NHI was high, with a median willingness to pay of UGX 25,000 (USD 6.8) and a mean WTP of UGX 28,950 (USD 7.8) per person, inclusive of 4 dependents per year. This level of willingness to pay was higher than the reported WTP in Nepal (71%) and in South Sudan (68%) among the informal sector (14, 25). However, the maximum amount of money people were willing to pay was lower than the 5.5 USD per person per month in Iran, 6.6 USD per person per month in Namibia, and 3 USD in Kenya per month (19, 25, 26). The differences in WTP across the different countries could be due to the differences in the socio-economic status of the different populations in the different countries. The high level of willingness to pay implies a high level of support for the proposed National Health Insurance scheme among the informal

Formatted: Font: 12 pt

Formatted: Font: 12 pt

Formatted: Font:

Formatted: Font: 12 pt

sector contributeworkers, contribute to the proposed NHIs and therefore, the need for government accelerate efforts of implementing the scheme. health insurance highlights high chances of the scheme being successful. However, the suggested premium being higher than the stated WTP may hinder many from participating in the scheme. But this also brings up the policy question of whether the WTP can sustain the scheme since premiums are the major source of funding for the scheme. Thus, there is a need for careful revision of the premiums before Parliament passes the NHI bill. The premiums should be based on the ability to pay.

Formatted: Font: 12 pt

The ability to pay is another important consideration when planning to implement health insurance, especially among the informal sector, whose incomes are unpredictable. A high number of participants believed that they could afford to make periodic contributions to the health insurance scheme in this study. This finding further highlights the potential for the NHI to succeed if the informal sector is included. However, the ability to pay needs to be considered when setting premiums, given the fact that less than 20% of the studied population were in the wealthiest category.

Although more than half of the respondents earned more than 100,000 UGX (27 USD) a month, there are substantial sections of the informal sector that will struggle to pay for health insurance, and therefore, these will require significant government subsidies to be included. This further highlights the need to consider the idea of making contributions as a proportion of people's earnings and wealth status because it would make people pay according to their ability and is more equitable than just having a flat figure for all people. Moreover, by virtue of the unpredictable nature of their income, informal sector workers expressed the desire to have premiums synchronized to earning cycles such as harvest seasons. This implies that the NHI will

have to explore flexible premium schedules for informal sector workers in order to enhance their uptake of the scheme. In Ethiopia, making premium systems more flexible eased payments (27).

In this study, having a savings group membership increased the likelihood of willingness to pay for the proposed national health insurance scheme. In Asian countries, saving groups were reported to be key in initiating community-based health insurance schemes (28). Savings groups provide members with a secure place to save money, generate a pool fund, and have the opportunity to borrow in small amounts. They also provide affordable basic insurance services and enable the community to meet the premiums(28). Local savings groups have become one of the most famous financial support systems in rural Uganda over the last decade (29). Savings and burial groups present an opportunity to leverage in enhancing access to health care in rural communities. This implies that as countries plan to initiate NHI, saving groups can become a great resource to tap into to ensure community participation and payment of the premiums for the scheme.

Participants belonging to the highest wealth category were more likely to pay for the proposed national health insurance scheme. This is in agreement with studies conducted in Nigeria, Ethiopia, and Ghana, which have reported that socio-economic factors such as poverty, income, or wealth status have a bearing on the ease or difficulty of enrolling in a given scheme, with high premiums deterring enrollment in schemes (30-32). Similar studies conducted in Sierra Leone and Iran showed that the WTP for the HI scheme depended on the monthly income of the respondent (26, 33) . In Nepal, belonging to a lower wealth quartile was associated with a lower willingness to pay for National Health Insurance Scheme (34). This therefore underscores the

need for income generating projects to be implemented within the communities to aid them make contributions for health insurance.

The use of traditional medicine was negatively associated with participation in the health insurance scheme. This is because members who use traditional medicine see no need to utilize modern health care services, making them less likely to pay for health insurance. Studies have shown that the presence and use of alternative medicine and other forms of healthcare, such as herbalists, negatively affects participation in a health insurance scheme since insurance requires regular use of modern medicine. Studies conducted in Cameroon, Burkina Faso, Nigeria, and India have reported that those who use modern medicine are more willing to pay for health insurance than those who use traditional medicine (35-37). In Uganda's pluralistic health system, the ministry of health and other mandated regulatory bodies need to institute measures that not only regulate the alternative sector but ultimately attract people to use modern medicine as opposed to traditional medicine.

It is not surprising that those who had never heard about health insurance had 50% less WTP compared to those who had prior information about health insurance. Indeed, the level of knowledge on how health insurance operates was very low, and statistical tests of significance could not be used in this study. However, one systematic review and meta-analysis of factors influencing WTP for voluntary contributary health insurance schemes in LMICs has revealed that knowledge and understanding of the functioning of the scheme positively influence the willingness to pay for and participate in the scheme (38, 39). As the Ugandan government moves to implement health insurance, information, education, and communication will be paramount to the success of the proposed scheme.

Study Limitations: As with most contingent valuation studies, the elicitation technique is always subject to bias, and the assignment of the first bid or amount is also biased. However, this was triangulated with an open-ended question and qualitative data. The low level of knowledge about health insurance among respondents was also a limitation for attaching value to the proposed scheme. This was solved by educating the respondent about health insurance before eliciting a willingness to pay. However, the study provides valuable insights on the willingness to pay for the proposed scheme among the informal sector workers who are often leftout in social protection programs. ~~Firstly, this study is unique in its assessment of the level of WTP, perceived ability to pay, and determinants of WTP for the proposed national health insurance scheme among the informal sector workers in Uganda, who constitute 80% of the Ugandan population. These findings are pertinent if the proposed NHI is to achieve adequate coverage. Secondly, the study applied a contingent valuation approach using a bidding game technique to hypothetically elicit the informal sector's willingness to pay. The highlight of this study is the high level of willingness to pay for NHI among informal sector workers, as well as their preferred modes of engagement. T~~hese findings provide valuable insights for fine-tuning the proposed NHI.

Formatted: Adjust space between Latin and Asian text, Adjust space between Asian text and numbers

Conclusions

This study indicated a high level of WTP, and the majority believed they could afford to pay for the health insurance ~~scheme.~~scheme. Therefore, this indicates that the informal sector is willing to make contribution to their health and thus feasible for the government to introduce a contributory scheme and the public bears some costs towards their health care. However, there

is need to revise the premiums bearing in mind that a large section of the informal sector will struggle to meet the proposed premiums, which will affect coverage. However, studies on the ability to pay need to be conducted, as this study is based on the perceived ability to pay for health insurance schemes. The high level of WTP indicates that the informal sector is willing to make a contribution to their health, making it feasible for the government to propose a flexible and affordable scheme within which the informal sector can bear some costs towards their health care. To support the ability to pay, there is a need to build local solidarity groups and strengthen local initiatives to aid poor members. The determinants for willingness to pay were occupation, wealth status, use of traditional medicine and having ever heard about health insurance. This implies that building trust among households and improving awareness are critical to the success of the scheme. There is need for MOH and the district health departments to design an information education campaign to sensitize the general public about how health insurance works and to buy in and build trust among the informal sector

Declarations

Consent for publication

Not Applicable

Availability of data and materials

The datasets analyzed for this study has been uploaded as a supplementary file. are available from the corresponding author on reasonable request.

Competing interests

The authors declare that they have no competing interests

Funding

The author(s) received no specific funding for this work

Authors' contributions

NN, SNK, AK conceived the study, NN collected, analyzed and wrote the first draft of the manuscript. SNK and AK provided reviews on all drafts of manuscript. All the authors read and approved the final manuscript.

Acknowledgements

We wish to thank the District Health Officers of Mayuge and Iganga districts for their cooperation, mobilization and ensuring that data collection went on smoothly. We also extend our sincere appreciation to the research assistants and all respondents in Iganga and Mayuge for sparing their time to contribute to this piece of work.

References

1. WHO. Universal health coverage (UHC): World Health Organization 2023 [Available from: https://www.who.int/news-room/fact-sheets/detail/universal-health-coverage-(uhc).
2. Sarker AR, Sultana M, Mahumud RA, Ahmed S, Islam Z, Morton A, et al. Determinants of enrollment of informal sector workers in cooperative based health scheme in Bangladesh. PloS one. 2017;12(7):e0181706.
3. Kiwanuka S, Ekirapa E, Peterson S, Okui O, Rahman MH, Peters D, et al. Access to and utilisation of health services for the poor in Uganda: a systematic review of available evidence. Transactions of the Royal Society of Tropical Medicine and Hygiene. 2008;102(11):1067-74.
4. WHO. Health Systems Financing: The Path to Universal Coverage. Geneva: World Health Organization 2010.
5. World Bank. Financing health in low-income countries. Washington DC: World Bank Group; 2010.
6. MoH. National Health Accounts 2018/19-2020/21 Report. 2021.

7. Tashobya CK, Ssengooba F, Cruz VO, Yates R, Murindwa G, McPake B. Health systems reforms in Uganda: processes and outputs. Christine Kirunga Tashobya FS and, Valeria Oliveira Cruz, editors. 2006.
8. UBOS. The National Population and Housing Census 2014 – Main Report. Kampala, Uganda: Uganda Bureau of Statistics; 2016.
9. Twaweza. Health Check: Ugandans' experiences and opinions on health services Uganda: Twaweza; 2019 [Available from: https://www.twaweza.org/go/szw-ug-health-check.
10. Gottret P, Schieber G. Health financing revisited: a practitioner's guide: The World Bank; 2006.
11. Xu K, Evans DB, Carrin G, Aguilar-Rivera AM, Musgrove P, Evans T. Protecting households from catastrophic health spending. Health affairs. 2007;26(4):972-83.
12. De Allegri M, Sanon M, Bridges J, Sauerborn R. Understanding consumers' preferences and decision to enrol in community-based health insurance in rural West Africa. Health policy. 2006;76(1):58-71.
13. Kyomugisha E, Buregyeya E, Ekirapa E, Mugisha J, Bazeyo W. Strategies for sustainability and equity of prepayment health schemes in Uganda. African health sciences. 2009;9(2).
14. Basaza R, Alier PK, Kirabira P, Ogubi D, Lako RLL. Willingness to pay for National Health Insurance Fund among public servants in Juba City, South Sudan: a contingent evaluation. International journal for equity in health. 2017;16(1):158.
15. Nguyen H, Knowles J. Demand for voluntary health insurance in developing countries: the case of Vietnam's school-age children and adolescent student health insurance program. Social Science & Medicine. 2010;71(12):2074-82.
16. Acharya A, Vellakkal S, Taylor F, Masset E, Satija A, Burke M, et al. Impact of National Health Insurance for the Poor and the Informal sector in Low-and Middle-Income Countries. London: EPPI-Centre, Social Science Research Unit, Institute of Education, University of London; 2012.
17. Basaza R, Criel B, Van der Stuyft P. Community health insurance in Uganda: why does enrolment remain low? A view from beneath. Health Policy. 2008;87(2):172-84.
18. Jowett M, Kutzin J, Organization WH. Raising revenues for health in support of UHC: strategic issues for policy makers. World Health Organization; 2015.
19. Gustafsson-Wright E, Asfaw A, van der Gaag J. Willingness to pay for health insurance: An analysis of the potential market for new low-cost health insurance products in Namibia. Social science & medicine. 2009;69(9):1351-9.
20. Russell S, Fox-Rushby J, Arhina D. Willingness and ability to pay for health care: a selection of methods and issues. Health Policy and Planning. 1995;10(1):94-101.
21. Mitchell RC, Carson RT. Using surveys to value public goods: the contingent valuation method: Rff Press; 2013.
22. Kish L. Survey sampling. Inc., New York.: John Wiley and Sons; 1965.
23. Whittington D, Smith VK, Okorafor A, Okore A, Liu JL, McPhail A. Giving respondents time to think in contingent valuation studies: a developing country application. Journal of Environmental Economics and Management. 1992;22(3):205-25.
24. Dong H, Kouyate B, Cairns J, Sauerborn R. A comparison of the reliability of the take-it-or-leave-it and the bidding game approaches to estimating willingness-to-pay in a rural population in West Africa. Social Science & Medicine. 2003;56(10):2181-9.
25. Barasa EW, Mwaura N, Rogo K, Andrawes L. Extending voluntary health insurance to the informal sector: experiences and expectations of the informal sector in Kenya. Wellcome open research. 2017;2.
26. Nosratnejad S, Rashidian A, Mehrara M, Sari AA, Mahdavi G, Moeini M. Willingness to pay for social health Insurance in Iran. Global journal of health science. 2014;6(5):154.

27. Habte A, Tamene A, Ejajo T, Dessu S, Endale F, Gizachew A, et al. Towards universal health coverage: The level and determinants of enrollment in the Community-Based Health Insurance (CBHI) scheme in Ethiopia: A systematic review and meta-analysis. PloS one. 2022;17(8):e0272959.
28. Shaikh BT, Noorani Q, Abbas S. Community based saving groups: an innovative approach to overcome the financial and social barriers in health care seeking by the women in the rural remote communities of Pakistan. Archives of Public Health. 2017;75(1):57.
29. Mutebi A, Muhumuza Kananura R, Ekirapa-Kiracho E, Bua J, Namusoke Kiwanuka S, Nammazi G, et al. Characteristics of community savings groups in rural Eastern Uganda: opportunities for improving access to maternal health services. Global health action. 2017;10(sup4):1347363.
30. Kotoh AM, Aryeetey GC, Van der Geest S. Factors that influence enrolment and retention in Ghana'National Health Insurance Scheme. International journal of health policy and management. 2018;7(5):443.
31. Odeyemi IA. Community-based health insurance programmes and the national health insurance scheme of Nigeria: challenges to uptake and integration. International journal for equity in health. 2014;13(1):20.
32. Mebratie AD, Sparrow R, Yilma Z, Alemu G, Bedi AS. Enrollment in Ethiopia's community-based health insurance scheme. World Development. 2015;74:58-76.
33. Jofre-Bonet M, Kamara J. Willingness to pay for health insurance in the informal sector of Sierra Leone. PloS one. 2018;13(5):e0189915.
34. Khatiwada B, Ghimire S, N S. Willingness to pay for health insurance in mangalbare village development committee of illam district. MOJ Public Health. 2017;5(2):43-6.
35. Mathiyazaghan K. Willingness to pay for rural health insurance through community participation in India. The International journal of health planning and management. 1998;13(1):47-67.
36. Donfouet HPP, Mahieu P-A, Malin E. Using respondents' uncertainty scores to mitigate hypothetical bias in community-based health insurance studies. The European Journal of Health Economics. 2013;14(2):277-85.
37. Oriakhi H, Onemolease E. Determinants of rural household's willingness to participate in community based health insurance scheme in Edo State, Nigeria. Studies on ethno-medicine. 2012;6(2):95-102.
38. Adebayo EF, Uthman OA, Wiysonge CS, Stern EA, Lamont KT, Ataguba JE. A systematic review of factors that affect uptake of community-based health insurance in low-income and middle-income countries. BMC health services research. 2015;15(1):543.
39. Dror DM, Hossain SS, Majumdar A, Koehlmoos TLP, John D, Panda PK. What factors affect voluntary uptake of community-based health insurance schemes in low-and middle-income countries? A systematic review and meta-analysis. PLoS One. 2016;11(8):e0160479.

Revised Manuscript with Track Changes

Willingness and p~~P~~erceived ability to pay for Uganda's p~~P~~roposed n~~N~~ational h~~H~~ealth i~~I~~nsurance s~~S~~cheme among i~~I~~nformal s~~S~~ector workers in Iganga and Mayuge districts, Uganda: a c~~C~~ontingent v~~V~~aluation m~~M~~ethod~~.~~

Noel Namuhani[1*], Angela N Kisakye[1, 2] and Suzanne N Kiwanuka[1]

Institutional affiliations

[1]Makerere u~~U~~niversity s~~S~~chool of public health, Department of h~~H~~ealth policy planning and management, Uganda

[2]African Field Epidemiology Network, Lugogo House, Plot 42, Lugogo By-Pass

*Corresponding a~~A~~uthor

Email: nnamuhani@musph.ac.ug

Abstract

Background: Access to healthcare remains a challenge, particularly among informal sector workers in most low-income countries, due to high out-of-pocket expenditures (OOPE). In Uganda, over 28% of healthcare costs are paid out of pocket. In response, Uganda has proposed a national health insurance scheme (NHIs). However, the willingness and ability to pay for the proposed NHIs ~~scheme~~ within the informal sector have not yet been explored in Uganda. This study assessed the willingness and perceived ability to pay for the proposed NHIs ~~scheme~~, along with its determinants, among informal sector workers in Iganga and Mayuge districts, Uganda.

Methodology: A cross-sectional study was conducted in Iganga and Mayuge districts between 10/04/2019 and 23/05/2019. A contingent valuation method using the bidding game technique was used to elicit the willingness to pay (WTP). A total of 853 informal sector workers, including farmers, commercial motorists, fishermen, and traders, were randomly sampled. Seven focus group discussions (FGD) were also conducted. Logistic regression was done to identify the determinants of willingness to pay for the proposed NHIs ~~scheme~~. Qualitative data was analyzed thematically.

Results: The majority 695/853, (81.5%) of the respondents were willing to pay for NHIs; the median WTP was UGX 25,000 (USD 6.8) annually; and 633/853, (74.2%) of the respondents believed that they were able to pay for the ~~health insurance~~. WTP~~Willingness to Pay~~ was significantly associated with being a fisher folk (AOR: 1.70 95%CI: 1.04-2.79, P = 0.035), being in the fourth wealth quintile (AOR: 2.98, 95% CI: 1.56–5.65), not hearing about health insurance (AOR: 0.50 95%CI: 0.23-0.86, P = 0.032), and not having saving group membership (AOR: 0.51,

95%CI: 0.34-0.76, P<0.001). Most of the FGD participants were willing to pay for the proposed scheme; however, some of the participants doubted their ability to pay for the scheme given their ~~high poverty~~extreme poverty levels and their unstable income.

Conclusion: The WTP ~~willingness to pay~~ for the NHIs ~~health insurance~~ in the informal sector is high. Therefore, it is viable for the government to extend NHIs to the informal sector. However, awareness building and ~~due consideration~~consideration of ~~high poverty~~extreme poverty levels in setting appropriate premiums should be a priority.

Key words: National Health insurance scheme, Willingness to pay, Ability to pay, informal sector, Contingent Valuation method.

Author s~~S~~ummary

Many Ugandans, especially those in the informal sector, struggle to access healthcare due to high out-of-pocket healthcare costs. To address this, Uganda plans to establish a national health insurance scheme. However, the extent to which informal sector workers — who constitute the largest portion of the labor force — are willing and able to pay remains unclear. This paper, therefore, seeks to explore the willingness and ability of informal sector workers to pay for the proposed national health insurance scheme.

To answer this objective, we interviewed 853 workers engaged in farming, fishing, trade, and commercial cycling in Iganga and Mayuge districts. We found that most respondents (82%) were willing to pay for the proposed health insurance, with an average annual amount of UGX 25,000

(about USD 6.80). Over 74% believed they could afford to pay for the proposed scheme, while others expressed concerns about their ability to pay due to poverty and unstable incomes.

Our study findings suggest that extending the health insurance scheme to informal sector workers is feasible. However, the government needs to consider affordability when setting premiums, given the ~~high levels~~elevated levels of poverty.

Introduction

Globally, there is a growing commitment to achieving Universal Health Coverage (UHC), which is an important element for achieving the Sustainable Development Goals (SDGs) (1). UHC aims to address the poor access to quality and affordable health services, which many African countries are grappling with, mainly due to high out-of-pocket expenditures(OOPE~~e~~) (2). In 2021, it was estimated that about 4.5 billion people (more than half) in the world were not fully covered by essential health services, while about 2 billion (a quarter) of the world's population were facing financial hardship due to out-of-pocket health spending (1).This put about 150 million people at risk of catastrophic healthcare costs annually, driving over 100 million people into poverty worldwide (2). According to the World Bank, direct modes of payment for health care services constitute 40% of health care expenditure in developing countries (3). However, developing countries suffer a higher burden of preventable disease, including the rising non communicable diseases (4). Thus, the need for health financing reforms to address this burden of disease, reduce OOPE and inequities.

Formatted: Font: (Asian) Japanese

In Uganda, although the national health accounts reported a reduction in the OOPE ~~out-of-pocket expenditure~~ on health as a share of current health expenditure (CHE) from 38.6% in the financial

year 2018/2019 to 27.4% in the financial year 2020/2021, the OOPE remains significantly high. This is compounded by the complex health financing landscape, where the general government health expenditure per capita allocation is very low estimated at 11.6 $, heavy reliance on donor funding accounting for 50.3% of the total health expenditure which has significantly reduced over the years. (5). Uganda abolished user fees in public health facilities, however, accessing healthcare is still associated with several expenses, including transport, drugs, diagnostics, and consultancy fees, among other expenses related to personnel and capital costs (6).

Globally, more than 60% of the employment occurs in the informal sector and in Uganda, over 80% of the labor force belongs to the informal sector (7). These include peasant farmers, commercial cyclists, fishermen, and those employed in unregistered or small-scale enterprises. It is estimated that 59% of the people in the informal sector in Eastern Uganda cannot afford to access quality health care due to high poverty levels and OOPE for health care (8).

Globally, the implementation of health insurance schemes is one of the major responses to addressing out-of-pocket payments for health care (2, 9) in low and middle-income countries. Health insurance has the ability to reduce financial barriers to health care access and protect individuals and families against the risk of unpredictable health care expenditures, hence aiding the attainment of UHC (10). This has improved access to healthcare, especially in developed countries.

High coverage and enrollment in the health insurance scheme are critical for the success and sustainability of the scheme, given that premiums constitute the proposed main source of funding for the scheme. However, enrolment in health insurance schemes remains low, ranging

from 2% to 40% in most African countries (11-13). The poor enrolment rates in the different countries have been attributed to a number of factors, including low incomes, large household sizes, long distances to health facilities, limited awareness, poor quality of health care services, inappropriate benefit packages, a lack of trust in the systems, and high illiteracy levels, especially in the informal sector (14-16). The informal sector workers are sometimes of low socioeconomic status compared to the formal sector workers, hence having a lower ability to pay for health insurance (16). Furthermore, the informal sector is not well organized; they have unpredictable incomes, making it difficult to enroll and register them into the NHIs~~ scheme~~ and collect regular contributions from them (13, 17).

Uganda is in the process of developing a NHIs~~ scheme~~ to address the high OOPE and access to services. However, given the high poverty levels among Ugandans and more so in the informal sector, the policy question of WTP ~~how much money people are willing to spend~~ and their ability to pay remains unanswered. Eastern Uganda is the poorest region in Uganda, with 35.7% of most households living below the poverty line (7). The WTP ~~willingness to pay~~ for the proposed NHIs ~~scheme~~, especially within the informal sector, which makes up 80% of Uganda's population, is critical to the achievement of equity in access to care and the core risk pooling function of a health insurance scheme. This would translate into high enrollment rates, which foster the financial sustainability of the schemes (10).

The NHI~~National Health Insurance~~ Bill, 2019, proposes that the scheme will be mandatory for all Ugandans, covering both formal and informal sector workers. Each person will be required to pay a premium, the amount of which will be determined by the board, to access the scheme. The

main sources of funding for the proposed scheme are premium contributions and government contributions for the indigent. Services will be provided in both public and accredited private health facilities. The bill was passed by Parliament in 2021 but was not assented to by the president due to disagreements among stakeholders who were not consulted. As of 2024, the bill is being discussed by the MoH~~inistry of Health~~ and other stakeholders before being re-tabled in the Parliament of Uganda.

There is a paucity of information on the willingness and ability to pay for the proposed ~~National Health Insurance (~~NHIs~~)~~ scheme, particularly within the informal sector. Understanding the level of willingness to enroll and the factors that influence the willingness to pay (WTP) is crucial in designing strategies to improve enrollment rates in the scheme. Therefore, this study aimed to document the WTP~~willingness to pay~~ and the perceived ability to pay for the proposed NHIs, along with the factors influencing willingness to pay among informal sector workers. The study's findings will be used by the Ministry of Health (MOH) to determine affordable premiums and design strategies to ensure maximum participation, especially among informal sector workers. The findings will also inform the NHIs ~~National Health Insurance Scheme~~ bill currently under discussion.

Materials and m~~M~~ethods

Study design: This was a cross-sectional study design that involved mixed methods of data collection. The quantitative and qualitative methods were conducted concurrently. The study was conducted from 10/04/2019 to 23/05/2019. This study adopted a contingent valuation method (CVM) using the bidding game technique to directly elicit or measure willingness to pay

for a hypothetical (not yet on the market) health insurance package (18). This approach has been used because it has been proven to be an effective way of assessing WTP ~~willingness to pay~~ with minimal bias for goods that are not presently available on the market. ~~The CVM~~CVM creates a hypothetical marketplace in which no actual transactions are made; hence, this approach has been successfully used for commodities that are not exchanged in regular markets. This approach makes the assumption that people have had no previous experience buying the health service that is going to be put on the market and instead asks people their ~~willingness to pay~~ WTP based on ~~the basis of~~ their expectations and the benefits attached to the service (19). In the absence of markets for public goods, this method presents consumers with hypothetical markets in which they have ~~the (hypothetical)~~the opportunity to buy ~~the good,~~a health insurance policy ~~in this case~~. Since the elicited WTP values are contingent upon the particular hypothetical market described to the respondent, this approach is called the contingent valuation method (20).

Study setting: The study was conducted in the Iganga and Mayuge districts of eastern Uganda. Iganga and Mayuge districts were selected purposively because they are the most poverty-hit districts in the country and 80% of the population lives in rural areas. They also have 80–85% of the population belonging to the informal sector (7). Iganga District is bordered by Kaliro District to the north, Bugweri District to the east, Mayuge District to the south, Jinja District to the southwest, and Luuka District to the west. With a total population of 504,197 people, 9.9% of the households are 5 km or more from the nearest health facility, and 19.9% are near a public health facility (UBOS, 2016). It is composed of 3 counties, 8 sub counties, 66 parishes, and 395 villages. The major economic activity is farming. Mayuge district ~~is located in~~is in south-eastern Uganda with a population of 473,2394 and a growth rate of 3.5%, and it has thirteen ~~subcounties~~sub

counties. The major economic activities are farming and fishing. Mayuge district is bordered by Lake Victoria to the south, as well as Jinja and Bugiri districts. It has a total of 33 landing sites where fishing activities take place. Uganda has a decentralized healthcare system, having both public and private health facilities. ~~The MoH at the center is the regulator and it will regulate the operations of the NHIs. At regional level, there are regional referral hospitals, and district level, where policies are implemented. At the district level exist district hospitals, health center IVs at county level, health center IIIs at the subcounty level, health center IIs at parish level and village health teams at village level.~~

Study p~~P~~opulation: The study targeted the four major categories of informal sector workers: 1) farmers; 2) fishermen; 3) commercial cyclists; and 4) the business community (traders and market vendors) in the study area. The informal sector was chosen because they make up the largest (80%) portion of the population, without which the sustainability of the scheme will be questionable.

Sample s~~S~~ize: The sample size was calculated using the Kish Leslie formula (21), considering a 95% confidence interval, a 50% expected level of WTP, a 5% level of precision, a design effect of 2, and a 10% non-response rate. This gave a total sample size of 853 informal sector workers. Four focus group discussions were conducted with women (four FGDs), and three FGDs were conducted with men (in the informal sector). This number depends on the level of ~~information saturation~~saturation of information. This was determined by doing preliminary analysis while still in the field.

Sampling pProcedure: Stratified random sampling was used in this study. For Iganga district, the informal sector workers were divided into three major categories (strata): farmers, commercial cyclists, traders/market vendors. In Mayuge district, the informal sector workers were divided into four categories: fishermen, farmers, commercial cyclists, and market vendors. A random sample from each stratum was taken in a number proportional to the stratum's size when compared to the population. In Iganga district, two sub counties were randomly selected, and five villages from each subcounty were also randomly selected. A proportionate sample of 286 households from the 10 villages was selected, and the household heads were systematically selected for interviews. Seven *bodaboda* stages located along main roads and junctions were randomly selected, and 10 cyclists available at the time of the study were randomly selected to participate in the interviews. A total of 84 traders were selected systematically from the two main markets for interviews. In Mayuge district, a total of 10/33 landing sites were randomly selected, and 248 fishermen were selected from the 10 landing sites proportionate to size. Sixty-two traders were randomly selected from three main markets systematically, and 62 bodaboda cyclists were randomly selected from six stages located along main roads in Mayuge town (Table 1). Men and women from each of the categories were purposively selected for the focus group discussions. These were selected and identified with the help of local leaders based on community members who can express themselves, but also are residents and working in the informal sectors of interest.

Table 1: Sampling procedure per category of informal sector

**Iganga district (**Iganga district planning office, 2018)				
**Category**	**Farmers/peasants**	**Commercial cyclists**	**Traders/vendors**	**Fisherfolk**

Proportion in the district	0.65	0.16	0.19	
Sample size	(0.65*440) =286	(0.16*440) =70	(0.19*440) =84	-
Selection procedure	2 sub counties (SC) and 5 villages from each SC were randomly selected. Households ~~proportionate~~are proportionate to size selected.	7 *Boda boda* stages in the town were randomly selected, cyclists on stage randomly selected to be interviewed	Traders were systematically selected for the interviews two main markets	-
Mayuge district				
**Category**	**Farmers/peasants**	**Commercial cyclists**	**Traders/vendors**	**Fisherfolk**
Proportion in the district	0.1	0.15	0.15	0.6
Sample size	(0.1*413) =41	(0.15*413) =62	(0.15*413) =62	(0.6*413) =248
Selection procedure	One sub county and two villages were randomly selected, the number of households were determined proportionate to size.	6 *Boda boda* stages in town were randomly selected, later cyclists on stage at that time were randomly selected to be interviewed	Traders were systematically selected from 3 main markets	10/33 landing sites were randomly selected. Fishermen at each site were randomly selected, proportionate to size
**Total samples**	**327**	**132**	**146**	**248**

Data collection methods and tools

The WTP ~~willingness to pay~~ was assessed using a CVM ~~contingent valuation method~~ using the "bidding game" technique. The bidding game was used to determine the maximum premium that a respondent is willing to pay for the proposed NHI plan. A bidding game technique was chosen because of its ease of implementation and its minimal biases. In addition, many studies have found the bidding game to be very reliable (22, 23). The respondent was asked if he or she was

willing to pay the starting bid. If the respondent agreed, the interviewer would raise the bid by 10% of the first bid and again ask if the respondent was willing to pay the new bid. The interviewer continued to present the different bids until the respondent expressed an unwillingness to pay. On the other hand, if the respondent expressed unwillingness to pay the starting bid, the interviewer lowered the bid by 10% and repeated the query. This was continued until a bid was reached where the respondent was willing to pay. The starting bid was 20,000 UGX (5.4 USD), since this was the average premium for beneficiaries in the existing community-based health insurance schemes in Luweero, Western Uganda, and Jinja. The proportion of those willing to pay, the average, and the median amount of money people were willing to pay were computed. The perceived ability to pay was measured by asking respondents whether they were able to pay premiums for health insurance per year and how much they could afford without foregoing other basic needs.

Both quantitative and qualitative data were collected co currently. A semi-structured questionnaire was used to collect quantitative data such as ~~sociodemographics~~sociodemographic variables, WTP, perceived ability to pay, and associated factors from informal sector workers. The authors developed the tool based on literature and their prior knowledge on NHIs. The tool also captured a few responses qualitatively. The data collection tools were translated to the local language and then back translated to English to check whether the translated questions still held informational validity. The principal investigator recruited and trained research assistants so that they were familiar with the statement of the problem, objectives of the study, sampling procedure, data collection tools, and plan for data collection. Research assistants were oriented to the bidding game technique for assessing WTP.

They were also taught the meaning of health insurance and how it intends to be operationalized in the country. Research assistants were also trained ~~on~~in the basic interview techniques, such as asking questions in a neutral manner, not showing by words or actions what answers were being expected of the study participants, and how to record answers, especially from open-ended questions, without interpreting them. The data collection tools were pre-tested in the neighboring district, Bugweri district. This was done to test the validity and ease of application of the tools. Filled electronic forms were checked at the point of data collection for completeness, and those found incomplete were completed before the respondent was discharged. The data were cleaned and edited by the research assistants before they submitted the complete forms to the server.

Focus group discussion (FGD) guides were also used to collect data from informal sector workers to understand the determinants of willingness to pay and their perceived ability to pay for the NHI. The FGDs were audio-taped. The FGD was composed of 6–8 participants. The discussion had a moderator and a note-taker, and the discussions lasted for an average of one hour. These were conducted in the local language, and these were audio recorded.

Data management and analysis: The quantitative data were downloaded from the server and imported into STATA 14 for cleaning and analysis. Descriptive statistics, including mean, median, standard deviations, frequencies, and percentages, were obtained. The information was presented in frequency distribution tables. Principal component analysis was done to generate wealth quintiles using the demographic health survey categorization. The wealth quintiles were generated based on nine household items, which included possession of a car, motorcycle,

bicycle, radio, television, mobile phone, piece of land, owning a house, and having animals. Inferential statistics were obtained using logistic regression. This was done in two stages: 1) bivariate analysis was done to determine the potential variables associated with willingness to pay for health insurance. Crude odds ratios at a 95% confidence interval were obtained to measure the potential association. 2) A multivariate analysis was done to determine the actual factors associated with WTP. All variables that were statistically significant at bivariate analysis and the ~~important factors~~key factors known in the literature to be statistically significant, such as socio-economic status, occupation, and potential confounders like residence, were included in the model. Variables were tested for multicollinearity before being included in the model. The model was built step-by-step while testing it using lfit until the best model was obtained. Variables that remained statistically significant at 95% confidence were considered the factors associated with WTP for health insurance. Adjusted odds ratios were obtained as measures of association.

Qualitative data was analyzed manually using a thematic-inductive approach. The recordings were transcribed verbatim and translated from the local language (Lusoga) to English. Transcripts were read by more than two independent analysts who identified codes, sub-themes, and then organized them into themes. The information was triangulated with the quantitative data findings to gain a deeper understanding.

Ethics statement

The study was approved by the Makerere University, School of Public Health Higher Degrees, Research and Ethics Committee (HDREC). At the district level, permission to conduct the study

was obtained from the local leaders and the District Health Office. Permission was also sought from subcounty chiefs and local council one chairpersons. The informed written consent of each individual participant was obtained at the start of the study. Confidentiality and anonymity were maintained by ~~by the use of~~using codes.

Results

Background c~~C~~haracteristics of the r~~R~~espondents

Of the 853 respondents who participated in the study, 327 (38.3%) were peasant farmers, 248929.1%) were fishermen, 146 (17.1%) were business people, and 132 (15.5%) were commercial cyclists. The mean age of the respondents was 37.0 years (SD ± 11.3). The majority 667/853 (78.2%) of the respondents were males, 783/853 (91.8%) of the household heads were males, and 618/853 (72.4%) resided in rural areas. The average household size was ~~5~~five persons. The median monthly income was UGX 150,000 (USD 40.5). Only 158/853 (18.5%) were in the wealthiest quintile, while 349/853 (41.0%) of the households were in the second and poorest wealth quintiles (Table 2).

Table 2: Background c~~C~~haracteristics of the r~~R~~espondents

Variable	Frequency (N=853)	Percentage (%)
**Sex of respondent**		
Male	667	78.2
Female	186	21.8
**Sex of the household head**		
Male	783	91.8
Female	70	8.2
**Residence**		
Rural	618	72.4
Urban	235	27.6

**Marital status**		
Married	694	81.4
Single	85	10.0
Separated	48	5.6
Widowed	26	3.0
**Age category**		
18-25	124	14.5
26-35	303	35.5
36-45	239	28.0
46-55	126	14.8
56 above	61	7.2
**Education level**		
None	51	6.0
Primary	445	52.2
Secondary	280	32.8
Tertiary	77	9.0
**Religious denominations ~~ion~~**		
Muslim	304	35.6
Protestant	276	32.4
Catholic	194	22.7
Born again	53	6.2
SDA	26	3.1
**Occupation**		
Farmer	327	38.3
Fishermen	248	29.1
Business	146	17.1
Bodaboda	132	15.5
**Wealth quintiles**		
Poorest (<-1.39)	172	20.2
Poor (-1.40 - -0.50)	177	20.8
Moderate (-0.51 -0.29)	163	19.1
Wealthy (0.30 -1.23)	183	21.5
Wealthiest(1.24-4.14)	158	18.5

Formatted: Font: (Asian) Japanese

Willingness to p~~P~~ay for the p~~P~~roposed NHI

The majority 695/853 (81.5%) of the respondents were willing to pay for the proposed NHIs. Of those willing to pay, the median WTP ~~willingness to pay~~ was UGX 25,000 (USD 6.8) while the mean WTP was UGX 28,950 (USD 7.8) per person, inclusive of ~~4~~four dependents per year. Only 3.8% protested contributing to the scheme, and 126/853 (14.7%) had

a zero ~~gave~~ WTP ~~zero~~ because they believed they were too poor to pay for national health insurance (Table 3).

Qualitatively, most of the FGD participants were also willing to make contributions for health insurance. However, others resented making contributions, as noted below:

> *"Sincerely, the government is meant to provide all the medical services. So, there is no way that you can lie to us and say that the government ~~can't~~cannot provide those services..."* **(FGD male-fishermen-Mayuge)**

A number of FGD participants were not willing to pay because of poverty, while others criticized the government's misuse of taxes collected from the tax payer on salaries for officials rather than on health services, as noted in the quotes below.

> *"The fact is that poverty is the biggest hindrance. Because at times you can fall sick, yet you ~~don't~~do not have money, and you resort to borrowing from colleagues who may not even be able to help you out because they also ~~don't~~do not have. So generally, I disagree with paying for health insurance."* **(FGD-Business Women, Mayuge District)**

> *"I am not willing to pay because we are always charged a lot of tax to register our businesses. That is all tax. Where does that tax go? Let them reduce the salary of Members of Parliament so that part of it is utilized to buy medicine. Members of Parliament have no importance in our area."* (**FFGD-Male-fishermen-Mayuge district)**

Willingness to p~~P~~ay with c~~C~~opayments:

A copayment is a cost-sharing arrangement where one covers a portion of the costs out of pocket each time one visits the health facility. Less than half, 309/695 (44.5%) of the respondents, were willing to co-pay. The mean co-pay amount was UGX 2,650 (USD 0.7), with a median of UGX 2000 (USD 0.5), each time someone accessed services. The average maximum amount of money that respondents were willing to pay with the copayment was UGX 23,000 (USD.6.2) and the median was UGX 20,000 (USD.5.4) (Table 3).

Table 3: Willingness to p~~P~~ay for the p~~P~~roposed NHI annually

WTP	Frequency (%)	Mean	Median
Protests (WTP=0)	32 (3.8)	0	0
WTP=0 (zero WTP)	126 (14.7)	0	0
WTP>0	695(81.5)	**28,950**	**25,000**
Total	853 (100.0)	23,600	20,000
**Copayment scenario**			
Amount willing to copay	309/695(44.5)	**2,650**	**2,000**
WTP with co payment	309/695(44.5)	23,000	20,000

Formatted: Font: (Asian) Japanese

Notes; *Protests-those protested attaching valuing to HI (WTP=0 but it has no economic sense). WTP=0 means Zero WTP~~willingness to pay is actually zero~~. WTP>0 that it is positive -WTP is above zero.*

Qualitatively, most of the FGD participants were not in support of copayments, as expressed by one of the FGD participants.

> *"I also do not support the idea that we pay money each time we visit the health facility, especially when we are paying every year. This will make people unable to access the services, and hence the problem of access to services will not have been solved. People are already poor, and that will mean people will continue to sell their property to go to health facilities."* (FGD Male Farmer)

Preferred m~~M~~ode of p~~P~~ayment

Most (54.1%) respondents preferred to pay annually, while 21.0% preferred to pay per harvesting season. Only 13.1% wanted to pay according to their monthly income, with the majority (63.1%) preferring to pay 1 percent of their monthly income, followed by those who were willing to pay 5%.

Perceived a~~A~~bility to p~~P~~ay for the NHIs~~Health Insurance Scheme~~

More than half 633/853 (74.2%) of the respondents believed that they were able to pay for health insurance, with a mean of UGX 23,000 (USD.6.2) and median of UGX 20,000 (USD.5.4) per person per year.

Regarding the perceived ability to pay, most of the FGD participants noted that most of the people are poor and most likely may not be able to make periodic subscriptions, especially if the money is raised more than what people earn. This was attested to, as indicated below:

> *"Sincerely speaking, some people cannot afford to pay money; for instance, you may find a very old person who is no longer earning anything, yet they also need health care."* **(FGD-Boda-boda-Iganga district)**

Another FGD participant, who was a business lady, noted that

> *"I support the idea. But not very much because of the issue of affordability. Not everyone can afford to pay, and note that we earn differently. Because some of us play the role of mother and father in the family, there are a lot of requirements to provide."* **(FGD Business w~~W~~omen, Iganga)**

Furthermore, most of the participants noted that setting premiums should be based on people's income and ability to pay, as noted below:

> *"Since everyone earns differently, the charges should depend on how much someone earns. I could be in a position to afford UGX 50,000, but yet my colleague cannot at all since people in the village are used to getting free medicine. When some people are asked to pay just 500shs for drugs, they will consider it unworthy because they know that drugs/medication are freely given. So, if possible, we charge according to people's income."* **(FGD: business women, Iganga district)**.

Determinants for level of w~~W~~illingness to p~~P~~ay for NHIs ~~Health Insurance Scheme~~

At bi-variable analysis, wealth quintiles, having savings group membership, having a family member with chronic illness, use of traditional medicine, and hearing about health insurance were statistically significant for willingness to pay for health insurance. The odds of being willing to pay when in the fourth wealth quintile were 2.88, compared to the odds of being willing to pay among those in the poorest category (COR: 2.88, 95% CI: 1.60–5.18, P<0.001). Not being in the saving group reduced the chances of being willing to pay for health insurance by 42% compared to those who had saving group memberships (COR: 0.58, 95% CI: 0.33-0.69, P<0.001) (Table 4).

After adjusting for any confounding factors, occupation, wealth, saving group membership, use of traditional medicine, and having heard about health insurance were significantly associated with the level of willingness to pay for the proposed health insurance scheme.

The odds of fishermen's willingness to pay for health insurance were 1.70 compared to those of farmers after adjusting for other factors (AOR: 1.70, 95% CI: 1.04–2.79, P = 0.035). Respondents in the fourth wealth quintile were 2.98 times more likely to pay for health insurance compared to those in the poorest wealth quintile (AOR: 2.98, 95% CI: 1.56–5.65). Respondents who were not in any saving group were 0.51 times less likely to pay for the proposed health insurance compared to those with saving group membership (AOR: 0.51, 95% CI: 0.34-0.76, P<0.001), and individuals who had never heard about HI were 50% less willing to pay for the proposed National Health Insurance compared to those who had ever heard about health insurance (AOR: 0.50, 95% CI: 0.23-0.86, P = 0.032) (Table 4).

Table 1: Multi variable analysis of factors associated with WTP for NHI scheme.

**Variable**	**Level of Willingness to pay**		**COR (95%CI)**	**AOR (95%CI)**
	**No (n=158)**	**Yes (n=695)**		
**Sex of respondent**				
Male	127(80.4)	540(77.7)	1.0	
Female	31(19.6)	155(22.3)	1.18(0.76-1.81)	1.30(0.80-2.11)
**Residence**				
Rural	110(69.6)	508(73.1)	1.0	
Urban	48(30.4)	187(26.9)	0.84(0.58-1.23)	1.09(0.52-2.26)
**Occupation**				
Farmer	60(38.0)	267(38.4)	1.0	
Business	32(20.2)	114(15.4)	0.80(0.49-1.30)	0.59(0.26-1.35)
Boda-boda	25(15.8)	107(15.4)	0.96(0.57-1.61)	1.04(0.50-2.18)
Fishermen	41(26.0)	207(29.8)	1.13(0.73-1.76)	1.70(1.04-2.79) *
**Wealth quintiles**				
Poorest	43(27.2)	129(18.6)	1.0	
Second poorest	32(20.3)	145(20.9)	1.51(0.90-2.53)	1.83(1.05-3.19) *

Formatted: Font: Italic

Middle	35(22.1)	128(18.4)	1.22(0.73-2.03)	1.23(0.70-2.14)
Fourth	19(12.0)	164(23.6)	2.88(1.60-5.18)	2.98(1.58-5.65) *
Wealthiest	29(18.4)	129(18.6)	1.48(0.87-2.52)	1.53(0.81-2.88)
**In saving group**				
Yes	49(31.0)	337(48.5)	1.0	
No	109(69.0)	358(51.5)	0.48(0.33-0.69)	0.51(0.34-0.76) **
**Family member with chronic illness**				
Yes	36(22.7)	241(34.7)	1.0	
No	122(77.2)	576(67.5)	0.56(0.37-0.83)	0.73(0.46-1.14)
**Ability to meet health care**				
Difficult	112(70.9)	571(82.2)	1.0	
Easy	46(29.1)	124(17.8)	0.53(0.36-0.78)	0.68(0.43-1.05)
**Use Traditional Medicine**				
No	59(38.8)	345(51.4)	1.0	
Yes	93(61.2)	326(48.6)	0.60(0.42-0.86)	0.71(0.48-0.92) *
**Ever heard about NHI**				
**Yes**	9(5.7)	83(11.9)	1.0	
**No**	149(94.3)	612(88.1)	0.45(0.22-0.91)	0.50(0.23-0.86) *

Note: ** *p<0.001, *P<0.05*

Discussion

This study assessed the WTP willingness and perceived ability to pay for the proposed National Health Insurance (NHIs) scheme and its determinants among informal sector workers in Iganga and Mayuge districts, Uganda. The study found that most informal sector workers were both willing and believed they were able to pay for the proposed NHI. WTPillingness to pay was significantly associated with occupation, wealth, awareness, and membership in saving groups.

In this study, most of the informal sector workers were willing to make contributions to the proposed NHIs. This could be perceived as a high support to NHIs among the informal sector. Our study findings are consistent with similar studies conducted in in Nepal (71%) and in South Sudan (68%) among the informal sector (13, 24). Similarly, systematic review and meta-analysis of WTP found that the pooled WTP for NHI in Africa and Asia was 71.0% (25). It is also consistent with a study conducted in Togo (92%) (26), 93% in Sierra Leone (27). The high level of WTP contributes to growing evidence showing the potential of informal sector workers participating in NHIs despite their nature of having unpredictable incomes and always poor (28).

This study found that the median ~~willingness to pay~~ WTP of USD 6.8 and a mean WTP of USD 7.8 per person, inclusive of ~~4~~ four dependents per year. This was slightly higher than the starting bid of USD 5.4 per year, thus the informal sector workers were willing to contribute more than what the study had hypothesized. However, the WTP was lower than the 5.5 USD per person per month in Iran, 6.6 USD per person per month in Namibia, and 3 USD in Kenya per month (18, 24, 29). The differences in WTP across the different countries could be due to the differences in the socio-economic status of the different populations in the different countries, and the differences in the way health insurance schemes are valued by the study populations. As Uganda discusses the NHIs bill, there is need to consider the low WTP amount among the informal sector in Uganda as compared to other countries and design affordable premiums for the informal sector. The ability to pay is another important consideration when planning to implement health insurance, especially among the informal sector, whose incomes are unpredictable. A high number of participants believed that they could afford to make periodic contributions to the health

insurance scheme in this study. This finding further highlights the potential for the NHI to ~~succeed if~~succeed if the informal sector is included in the scheme. However, the ability to pay needs to be considered when setting premiums, given the fact that less than 20% of the studied population were in the wealthiest category.

Although more than half of the respondents earned more than 100,000 UGX (27 USD) a month, there are substantial sections of the informal sector that will struggle to pay for health insurance, and therefore, these will require significant government subsidies to be included. This further highlights the need to consider the idea of making contributions as a proportion of people's earnings and wealth status because it would make people pay according to their ability and is more equitable than just having a flat figure for all people. Moreover, by virtue of the unpredictable nature of their income, informal sector workers expressed the desire to have premiums synchronized to earning cycles such as harvest seasons. This implies that the NHI will have to explore flexible premium schedules for informal sector workers in order to enhance their uptake of the scheme. In Ethiopia, making premium systems more flexible eased payments (30).

In this study, having a savings group membership increased the likelihood of willingness to pay for the proposed national health insurance scheme. In Asian countries, saving groups were reported to be key in initiating community-based health insurance schemes (31). Savings groups provide members with a secure place to save money, generate a pool fund, and have the opportunity to borrow in small amounts. They also provide affordable basic insurance services and enable the community to meet the premiums(31). Local savings groups have become one of the most famous financial support systems in rural Uganda over the last decade (32). Savings and burial groups present an opportunity to leverage in enhancing access to health care in rural

communities. ~~This implies that as countries plan to initiate NHI, saving groups can become a great resource to tap into to ensure community participation and payment of the premiums for the scheme.~~

Participants belonging to the highest wealth category were more likely to pay for the proposed national health insurance scheme. This is in agreement with studies conducted in Nigeria, Ethiopia, and Ghana, which have reported that socio-economic factors such as poverty, income, or wealth status have a bearing on the ease or difficulty of enrolling in a given scheme, with high premiums deterring enrollment in schemes (33-35). Similar studies conducted in Sierra Leone and Iran showed that the WTP for the HI scheme depended on the monthly income of the respondent (27, 29) . In Nepal, belonging to a lower wealth quartile was associated with a lower willingness to pay for National Health Insurance Scheme (36). Thus the WTP for the NHIs unavoidably depends on the wealth and incomes of the individuals. This needs to be considered by policy makers when designing premiums. Measure to boost people incomes will be critical to the success of the scheme. ~~This therefore underscores the need for income generating projects to be implemented within the communities to aid them make contributions for health insurance.~~

The use of traditional medicine was negatively associated with participation in the health insurance scheme. This is because members who use traditional medicine see no need to utilize modern health care services, making them less likely to pay for health insurance. Studies have shown that the presence and use of alternative medicine and other forms of healthcare, such as herbalists, negatively affects participation in a health insurance scheme since insurance requires regular use of modern medicine. Studies conducted in Cameroon, Burkina Faso, Nigeria, and India have reported that those who use modern medicine are more willing to pay for health insurance

than those who use traditional medicine (37-39). In Uganda's pluralistic health system, the ministry of health and other mandated regulatory bodies need to institute measures that not only regulate the alternative sector but ultimately attract people to use modern medicine as opposed to traditional medicine.

It is not surprising that those who had never heard about health insurance had 50% less WTP compared to those who had prior information about health insurance. Indeed, the level of knowledge on how health insurance operates was ~~very low~~extremely low, and statistical tests of significance could not be used in this study. This was similar to studies conducted in other Africa and Asia where the knowledge was reported to be low (25). A systematic review and meta-analysis of factors influencing WTP for voluntary contributary health insurance schemes in LMICs revealed that knowledge and understanding of the functioning of the scheme positively influence the WTP~~willingness to pay for~~ and participatione~~e~~ in the scheme (40, 41). ~~As the Ugandan government moves to implement health insurance, information, education, and communication will be paramount to the success of the proposed scheme.~~

Implications for policy and practice

Formatted: Font: Bold

This study offers key policy insights as Uganda design a NHIs to include the informal sector. The high level of WTP is promising, but the relatively modest amounts they were prepared to contribute should not be overlooked. For the scheme to be inclusive and financially sustainable, the premiums must reflect what people can realistically afford. A one-size-fits-all approach is unlikely to succeed. Therefore, the MoH should design premiums according to people's incomes and have flexible payment schedules aligned with seasonal income cycles such as post-harvest

periods for farmers. The government should also consider subsidies for the very poor informal sector workers so that inequity gaps are not widened further. Furthermore, to support the ability to pay, the MoH working with partners should build local solidarity groups and local initiatives to assist poorer members to afford premiums.

The strong connection between being part of a savings group and WTP scheme highlights a real opportunity. These groups are already trusted and embedded in their communities, which makes them well-placed to support things like collecting premiums and encouraging participation. By working more closely with them, the scheme could become much more effective on the ground. Local governments and health officials could team up with savings groups to identify members, collect payments, and spread the word about the benefits of joining. This will not only boost enrollment but also build a stronger sense of community ownership and accountability, something that top-down approaches often miss.

One thing that stood out clearly in both the surveys and the focus groups was that many people don’t fully understand how health insurance works. This lack of awareness could seriously affect how many people sign up—and how much they trust the system. That’s why it’s so important for the Ministry of Health and district health teams to focus on public education. They could use tools like local radio, community gatherings, and respected local leaders to reach people, especially in rural and hard-to-reach areas. The goal should be to break down what health insurance is, explain where the money goes, and reassure people that they’ll get good care. Just as crucial is making sure the care provided under the scheme feels better, like shorter waiting times, reliable access to medicines, and being treated with dignity can really help people feel that the system is working for them and worth being part of.

Future research needs to consider evaluating the ~~Additionally, studies on the~~ actual ability to ~~pay~~ pay and how NHIs and the broader social health protection can be extended to the informal sector.

Study ~~Limitations~~limitations: As with most contingent valuation studies, the elicitation technique is always subject to bias, and the assignment of the first bid or amount is also prone to bias. However, this was triangulated with ~~an open~~open-ended question and qualitative data. The low level of knowledge about health insurance among respondents was another limitation in attaching value to the proposed scheme. This was addressed by educating the respondents about health insurance before eliciting their willingness to pay. Despite these limitations, the study provides valuable insights into the willingness to pay for the proposed scheme among informal sector workers, who are often excluded from social protection programs.

Conclusions

This study indicated that the informal sector workers are willing to make contributions to the proposed NHIs. However, the premium structure should be designed with careful consideration of the ability of the informal sector to pay, given the high levels of poverty and unpredictable incomes. The study also highlights that ~~a high level of willingness to pay (WTP), with the majority believing they could afford to pay for the health insurance scheme. This suggests that the informal sector is willing to contribute to their health, making it feasible for the government to introduce a contributory scheme, with the public bearing some costs towards their healthcare. However, there is a need to revise the proposed premiums, considering that a large portion of the informal sector may struggle to meet them, which could affect coverage.~~

~~Additionally, studies on the actual ability to pay should be conducted, as this study is based on the perceived ability to pay for health insurance schemes. To support the ability to pay, local solidarity groups should be built, and local initiatives to assist poorer members should be strengthened.~~

~~The determinants of willingness to pay included~~ occupation, wealth status, use of traditional medicine, ~~and~~ prior knowledge of health insurance and trust influence WTP for NHIs among the informal sector workers. Creating awareness and fostering trust is critical to the success of the proposed NHIs.

~~This suggests that building trust among households and improving awareness are critical to the success of the scheme. There is a need for the Ministry of Health (MOH) and district health departments to design an information and education campaign to sensitize the general public about how health insurance works, encouraging buy-in and fostering trust, especially among the informal sector.~~

Declarations

Consent for publication

Not Applicable

Availability of data and materials

The dataset analyzed for this study has been uploaded as a supplementary file.

Competing interests

The authors declare that they have no competing interests

Funding

The author(s) received no specific funding for this ~~work~~work.

Authors' contributions

NN, SNK, AK conceived the study, NN collected, ~~analyzed~~analyzed, and wrote the first draft of the manuscript. SNK and AK provided reviews on all drafts of manuscript~~.~~. All the authors read and approved the final manuscript.

Acknowledgements

We ~~wish to thank~~thank the District Health Officers of Mayuge and Iganga districts for their cooperation, mobilization and ensuring that data collection went on smoothly. We also extend our sincere appreciation to the research assistants and all respondents in Iganga and Mayuge for sparing their time to contribute to this piece of work.

References

1. WHO. Universal health coverage (UHC): World Health Organization 2023 [Available from: https://www.who.int/news-room/fact-sheets/detail/universal-health-coverage-(uhc).
2. WHO. Health Systems Financing: The Path to Universal Coverage. Geneva: World Health Organization 2010.
3. World Bank. Financing health in low-income countries. Washington DC: World Bank Group; 2010.
4. WHO. Noncommunicable diseases: World Health Organization 2024 [Available from: https://www.who.int/news-room/fact-sheets/detail/noncommunicable-diseases.
5. MoH. National Health Accounts 2018/19-2020/21 Report. 2021.
6. Tashobya CK, Ssengooba F, Cruz VO, Yates R, Murindwa G, McPake B. Health systems reforms in Uganda: processes and outputs. Christine Kirunga Tashobya FS and, Valeria Oliveira Cruz, editors. 2006.
7. UBOS. The National Population and Housing Census 2014 – Main Report. Kampala, Uganda: Uganda Bureau of Statistics; 2016.

8. Twaweza. Health Check: Ugandans' experiences and opinions on health services Uganda: Twaweza; 2019 [Available from: https://www.twaweza.org/go/szw-ug-health-check.
9. Gottret P, Schieber G. Health financing revisited: a practitioner's guide: The World Bank; 2006.
10. Xu K, Evans DB, Carrin G, Aguilar-Rivera AM, Musgrove P, Evans T. Protecting households from catastrophic health spending. Health affairs. 2007;26(4):972-83.
11. De Allegri M, Sanon M, Bridges J, Sauerborn R. Understanding consumers' preferences and decision to enrol in community-based health insurance in rural West Africa. Health policy. 2006;76(1):58-71.
12. Kyomugisha E, Buregyeya E, Ekirapa E, Mugisha J, Bazeyo W. Strategies for sustainability and equity of prepayment health schemes in Uganda. African health sciences. 2009;9(2).
13. Basaza R, Alier PK, Kirabira P, Ogubi D, Lako RLL. Willingness to pay for National Health Insurance Fund among public servants in Juba City, South Sudan: a contingent evaluation. International journal for equity in health. 2017;16(1):158.
14. Nguyen H, Knowles J. Demand for voluntary health insurance in developing countries: the case of Vietnam's school-age children and adolescent student health insurance program. Social Science & Medicine. 2010;71(12):2074-82.
15. Acharya A, Vellakkal S, Taylor F, Masset E, Satija A, Burke M, et al. Impact of National Health Insurance for the Poor and the Informal sector in Low-and Middle-Income Countries. London: EPPI-Centre, Social Science Research Unit, Institute of Education, University of London; 2012.
16. Basaza R, Criel B, Van der Stuyft P. Community health insurance in Uganda: why does enrolment remain low? A view from beneath. Health policy. 2008;87(2):172-84.
17. Jowett M, Kutzin J, Organization WH. Raising revenues for health in support of UHC: strategic issues for policy makers. World Health Organization; 2015.
18. Gustafsson-Wright E, Asfaw A, van der Gaag J. Willingness to pay for health insurance: An analysis of the potential market for new low-cost health insurance products in Namibia. Social science & medicine. 2009;69(9):1351-9.
19. Russell S, Fox-Rushby J, Arhina D. Willingness and ability to pay for health care: a selection of methods and issues. Health Policy and Planning. 1995;10(1):94-101.
20. Mitchell RC, Carson RT. Using surveys to value public goods: the contingent valuation method: Rff Press; 2013.
21. Kish L. Survey sampling. Inc., New York.: John Wiley and Sons; 1965.
22. Whittington D, Smith VK, Okorafor A, Okore A, Liu JL, McPhail A. Giving respondents time to think in contingent valuation studies: a developing country application. Journal of Environmental Economics and Management. 1992;22(3):205-25.
23. Dong H, Kouyate B, Cairns J, Sauerborn R. A comparison of the reliability of the take-it-or-leave-it and the bidding game approaches to estimating willingness-to-pay in a rural population in West Africa. Social Science & Medicine. 2003;56(10):2181-9.
24. Barasa EW, Mwaura N, Rogo K, Andrawes L. Extending voluntary health insurance to the informal sector: experiences and expectations of the informal sector in Kenya. Wellcome open research. 2017;2.
25. Bayked EM, Assfaw AK, Toleha HN, Zewdie S, Biset G, Ibirongbe DO, et al. Willingness to pay for National Health Insurance Services and Associated Factors in Africa and Asia: a systematic review and meta-analysis. Frontiers in Public Health. 2024;12:1390937.
26. Djahini-Afawoubo DM, Atake E-H. Extension of mandatory health insurance to informal sector workers in Togo. Health economics review. 2018;8:1-13.
27. Jofre-Bonet M, Kamara J. Willingness to pay for health insurance in the informal sector of Sierra Leone. PloS one. 2018;13(5):e0189915.
28. ILO. Women and men in the informal economy: A statistical picture. Geneva ILO; 2018.

29. Nosratnejad S, Rashidian A, Mehrara M, Sari AA, Mahdavi G, Moeini M. Willingness to pay for social health Insurance in Iran. Global journal of health science. 2014;6(5):154.
30. Habte A, Tamene A, Ejajo T, Dessu S, Endale F, Gizachew A, et al. Towards universal health coverage: The level and determinants of enrollment in the Community-Based Health Insurance (CBHI) scheme in Ethiopia: A systematic review and meta-analysis. PloS one. 2022;17(8):e0272959.
31. Shaikh BT, Noorani Q, Abbas S. Community based saving groups: an innovative approach to overcome the financial and social barriers in health care seeking by the women in the rural remote communities of Pakistan. Archives of Public Health. 2017;75(1):57.
32. Mutebi A, Muhumuza Kananura R, Ekirapa-Kiracho E, Bua J, Namusoke Kiwanuka S, Nammazi G, et al. Characteristics of community savings groups in rural Eastern Uganda: opportunities for improving access to maternal health services. Global health action. 2017;10(sup4):1347363.
33. Kotoh AM, Aryeetey GC, Van der Geest S. Factors that influence enrolment and retention in Ghana'National Health Insurance Scheme. International journal of health policy and management. 2018;7(5):443.
34. Odeyemi IA. Community-based health insurance programmes and the national health insurance scheme of Nigeria: challenges to uptake and integration. International journal for equity in health. 2014;13(1):20.
35. Mebratie AD, Sparrow R, Yilma Z, Alemu G, Bedi AS. Enrollment in Ethiopia's community-based health insurance scheme. World Development. 2015;74:58-76.
36. Khatiwada B, Ghimire S, N S. Willingness to pay for health insurance in mangalbare village development committee of illam district. MOJ Public Health. 2017;5(2):43-6.
37. Mathiyazaghan K. Willingness to pay for rural health insurance through community participation in India. The International journal of health planning and management. 1998;13(1):47-67.
38. Donfouet HPP, Mahieu P-A, Malin E. Using respondents' uncertainty scores to mitigate hypothetical bias in community-based health insurance studies. The European Journal of Health Economics. 2013;14(2):277-85.
39. Oriakhi H, Onemolease E. Determinants of rural household's willingness to participate in community based health insurance scheme in Edo State, Nigeria. Studies on ethno-medicine. 2012;6(2):95-102.
40. Adebayo EF, Uthman OA, Wiysonge CS, Stern EA, Lamont KT, Ataguba JE. A systematic review of factors that affect uptake of community-based health insurance in low-income and middle-income countries. BMC health services research. 2015;15(1):543.
41. Dror DM, Hossain SS, Majumdar A, Koehlmoos TLP, John D, Panda PK. What factors affect voluntary uptake of community-based health insurance schemes in low-and middle-income countries? A systematic review and meta-analysis. PLoS One. 2016;11(8):e0160479.

Date: 20th /December/2024

The Editor,
PLOS Global Public Health

Dear Sir/Madam,

Re; **Address of Comments for the manuscript PGPH-D-24-01717**

We thank you and the reviewers for the time taken to review our manuscript. We appreciate all the comments and suggestions given. Below is how we attempted to address them and improve the paper.

**Comment**	**How it was addressed**
Journal requirements	
1. In the online submission form, you indicated that "The datasets analyzed for this study are available from the corresponding author on reasonable request.".<br><br>All PLOS journals now require all data underlying the findings described in their manuscript to be freely available to other researchers, either<br>1. In a public repository,<br>2. Within the manuscript itself, or<br>3. Uploaded as supplementary information.	The dataset has been uploaded as a supplementary information and, we have changed the statement accordingly to reflect that the dataset has been uploaded as a supplementary file.
2. Please provide an Author Summary. This should appear in your manuscript between the Abstract (if applicable) and the Introduction, and should be 150–200 words long. The aim should be to make your findings accessible to a wide audience that includes both scientists and non-scientists.	This has been added
3. We have noticed that you have cited Table 4 in the manuscript file but there are no corresponding tables in the manuscript. Please amend your manuscript to include this table, noting that tables should not be uploaded as individual files.	Actually table 4, does exist on page 19, with subheading<br><br>*Table 4: Multi variable analysis of factors associated with WTP for NHI scheme.*
Reviewer #1: The manuscript addresses an important aspect of health financing in Uganda. The manuscript is also very well written with	Thank you so much for your kind comments. We have addressed the comments noted, as below.

sufficient transparency about the approach and its limitations. I have included a few general comments for the authors' consideration in shaping the writeup.	
1. I understand that bill has not been passed yet but it will be good to have a sense of the proposed funding sources for the scheme. It is quite unclear when the paper suggest that the scheme would unsustainable without informal sector subscribers. For example in Ghana, premiums form less than 1% of the funding and therefore increasing subscription without commensurate funding is actually a threat to the scheme.	The main sources of funding for the scheme are premium contributions and government allocations for the indigents. This has been included.
2. It will be great if possible to also have a sense of current health financing landscape particularly in the regions being studied. How are they paying for health care? What is the level of insurance penetration?	A paragraph on health financing landscape in Uganda is given, which is assumed to that of the two studied districts.
3. The variation in WTP by age would have been very interesting even though i notice it was omitted from the multivariate analysis.	At bi variable analysis, age was not significantly associated with WTP, and hence omitted from the mult-variate analysis model. Furthermore, adding it to the model shows no significant effect on the determinants for WTP in this study.
4. In the discussion it will be good to keep in mind that a wholesale call for increased enrolment will also come with budget (cost) implications. At the moment it is not clear if the premiums can support the funds required for the scheme.	Since the main source of funding is premium contributions, increased enrollment will further generate more revenue for the scheme, and less constrain the budget. This point has been emphasized in the discussion.
Thank you for putting together this interesting manuscript.	Thank you so much.
Reviewer #2: This study sought to explore the underlying factors influencing willingness and ability to pay for NHI in Uganda. The topic is relevant for the ongoing health system reforms in Uganda, particularly in the area of healthcare financing towards achieving UHC. That said, there are pertinent issues requiring authors' attention.	Thank you for raising the pertinent issues. We have improved these as below.

**Introduction**<br>I think the entire manuscript must be revised to demonstrate its relevance internationally beyond its current narrow scope.	We have tried to bring in the global perspectives in the background and discussions, especially on its relevance to achieving global goals such as universal health coverage and sustainable development goals.
Define OOPE at first use. OOP is defined but not OOPE	This has been defined
It is not clear what problem this study seeks to address. Please elaborate on statement of the problem. Also, please what knowledge gap is it aiming to bridge? Further, linked to the problem statement, the justification for the study is weak, authors need to strengthen it.	The problem statement has been strengthened in the background, and the justification for the study.
Please include a paragraph on the structure of the Ugandan health system, indicating clearly service coverage and payment mechanisms, and levels of the service delivery structure that services under the NHI are provided, including where the private facilities stand in the scheme of things. Also, elaborate on the NHI in terms of focus, coverage, access etc.	The structure of the health system has been included in the methods under study settings.<br>In the background we have added details of the proposed NHI in terms of focus, who will access and be covered by the scheme.
Methods<br>Authors wrote on page 9 lines 102 – 104 that this was a cross-sectional study design that involved mixed methods of data collection. Please indicate the type of mixed method study and how it was operationalized.	This was a concurrent mixed method and this has been specified
In describing the contingent valuation method (CVM), I think it would be appropriate for authors to weave in the relevance of this study and how it fits with the model.	The relevance of the study has been included
Authors have done a good job at describing the sampling technique used, but not how the FGD participants were selected. Please indicate the approach used?	How FGDs participants were purposively selected. This has been explained
Discussion<br>I suggest authors summarize the key findings in beginning paragraph, then proceed to discuss. The	The first paragraph summarizing findings has been added

discussion is haphazard and failed to properly situate the findings on the reasons for the WTP for the proposed NHI, ability to pay and NHI coverage within existing body of knowledge and contextual dynamics. Please review and properly structure the whole section.	
In your discussion section, you must relate this to the international literature and point out the policy implications or implications for practice.	This has been emphasized
Please include a section on the study's implication for policy and practice.	The implications for policy and practice have been added in the discussions at the end of each of discussion point.
Conclusion<br>If WTP level is high, and the majority believed they could afford to pay for the health insurance, then I think there is the need for a strong conclusion and recommendations to make this study relevant in the scheme of things. In fact, there is no clear implication for policy. Please provide a clear and succinct but cogent conclusion based on your study findings – which may be inclusive of recommendations relevant for similar contextual settings.	This section has been strengthened with implications of the study findings and recommendations
General comments<br>The paper will benefit from proofreading. There are a number of sentence construction issues and, incoherent statements and paragraphs that need attention.	We have proof read the paper and corrected the grammatical issues

Thank you for your consideration.

Sincerely,

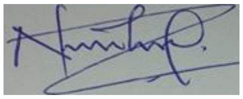

Noel Namuhani, Research associate, Makerere University School of Public Health

Date: 8th /May/2025

The Editor,
PLOS Global Public Health

Dear Sir/Madam,

Re: Address of Comments for the manuscript PGPH-D-24-01717R2

Please, below is how we addressed the comment regarding potentially identifying information in the data set.

**Comment**	**How it was addressed**
We note that there is potentially-identifying data in the Supporting Information file 'Dataset-De identified.xlsx' (specific location data). Therefore, we have hidden this file in the system to prevent it from being published mistakenly. Prior to sharing human research participant data, authors should consult with an ethics committee to ensure data are shared in accordance with participant consent and all applicable local laws. Data sharing should never compromise participant privacy. It is therefore not appropriate to publicly share personally identifiable data on human research participants.	Thank you for your observation. The data set has been fully de-identified and re uploaded. We removed columns containing identifying information such addresses, age, names. We have replaced the household codes with numbers so that these can not be traced.

Thank you for your consideration.

Sincerely,

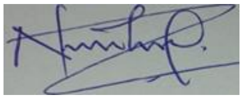

Noel Namuhani, Research associate, Makerere University School of Public Health

Date: 13th /May/2025

The Editor,
PLOS Global Public Health

Dear Sir/Madam,

Re: Address of Comments for the manuscript PGPH-D-24-01717R2

Please, below is how we addressed the comment regarding potentially identifying information in the data set.

**Comment**	**How it was addressed**
We note that there is potentially-identifying data in the Supporting Information file 'Dataset-De identified.xlsx' (specific location data). Therefore, we have hidden this file in the system to prevent it from being published mistakenly. Prior to sharing human research participant data, authors should consult with an ethics committee to ensure data are shared in accordance with participant consent and all applicable local laws. Data sharing should never compromise participant privacy. It is therefore not appropriate to publicly share personally identifiable data on human research participants.	Thank you for your observation. The data set has been fully de-identified and re uploaded as supporting information and legend provided in the manuscript. We removed columns containing identifying information such addresses, age, names. We have replaced the household codes with numbers so that these can not be traced.

Thank you for your consideration.

Sincerely,

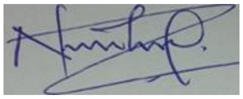

Noel Namuhani, Research associate, Makerere University School of Public Health
