# Supplementary material for: Willingness and perceived ability to pay for Uganda’s proposed national health insurance scheme among informal sector workers in Iganga and Mayuge districts, Uganda: A contingent valuation method
Source: PLOS Glob Public Health. 2026 Mar 11;6(3):e0003603. doi: 10.1371/journal.pgph.0003603 (PMC12978500; doi:10.1371/journal.pgph.0003603)
Supplement: Attachment — Submitted filename: Response to comments 20-12-24.docx [file pgph.0003603.s003.docx]

Date: 20^th^ /December/2024

**The Editor,**

PLOS Global Public Health

Dear Sir/Madam,

Re; **Address of Comments for the manuscript PGPH-D-24-01717**

We thank you and the reviewers for the time taken to review our manuscript. We appreciate all the comments and suggestions given. Below is how we attempted to address them and improve the paper.

| **Comment** | **How it was addressed** |
| --- | --- |
| ***Journal requirements*** |  |
| 1. In the online submission form, you indicated that "The datasets analyzed for this study are available from the corresponding author on reasonable request.".    All PLOS journals now require all data underlying the findings described in their manuscript to be freely available to other researchers, either  1. In a public repository,  2. Within the manuscript itself, or  3. Uploaded as supplementary information. | The dataset has been uploaded as a supplementary information and, we have changed the statement accordingly to reflect that the dataset has been uploaded as a supplementary file. |
| 2. Please provide an Author Summary. This should appear in your manuscript between the Abstract (if applicable) and the Introduction, and should be 150–200 words long. The aim should be to make your findings accessible to a wide audience that includes both scientists and non-scientists. | This has been added |
| 3. We have noticed that you have cited Table 4 in the manuscript file but there are no corresponding tables in the manuscript. Please amend your manuscript to include this table, noting that tables should not be uploaded as individual files. | Actually table 4, does exist on page 19, with subheading  *Table 4: Multi variable analysis of factors associated with WTP for NHI scheme.* |
| ***Reviewer #1:*** The manuscript addresses an important aspect of health financing in Uganda. The manuscript is also very well written with sufficient transparency about the approach and its limitations. I have included a few general comments for the authors' consideration in shaping the writeup. | Thank you so much for your kind comments. We have addressed the comments noted, as below. |
| 1. I understand that bill has not been passed yet but it will be good to have a sense of the proposed funding sources for the scheme. It is quite unclear when the paper suggest that the scheme would unsustainable without informal sector subscribers. For example in Ghana, premiums form less than 1% of the funding and therefore increasing subscription without commensurate funding is actually a threat to the scheme. | The main sources of funding for the scheme are premium contributions and government allocations for the indigents. This has been included. |
| 2. It will be great if possible to also have a sense of current health financing landscape particularly in the regions being studied. How are they paying for health care? What is the level of insurance penetration? | A paragraph on health financing landscape in Uganda is given, which is assumed to that of the two studied districts. |
| 3. The variation in WTP by age would have been very interesting even though i notice it was omitted from the multivariate analysis. | At bi variable analysis, age was not significantly associated with WTP, and hence omitted from the mult-variate analysis model. Furthermore, adding it to the model shows no significant effect on the determinants for WTP in this study. |
| 4. In the discussion it will be good to keep in mind that a wholesale call for increased enrolment will also come with budget (cost) implications. At the moment it is not clear if the premiums can support the funds required for the scheme. | Since the main source of funding is premium contributions, increased enrollment will further generate more revenue for the scheme, and less constrain the budget. This point has been emphasized in the discussion. |
| Thank you for putting together this interesting manuscript. | Thank you so much. |
| ***Reviewer #2:*** This study sought to explore the underlying factors influencing willingness and ability to pay for NHI in Uganda. The topic is relevant for the ongoing health system reforms in Uganda, particularly in the area of healthcare financing towards achieving UHC. That said, there are pertinent issues requiring authors’ attention. | Thank you for raising the pertinent issues. We have improved these as below. |
| **Introduction** I think the entire manuscript must be revised to demonstrate its relevance internationally beyond its current narrow scope. | We have tried to bring in the global perspectives in the background and discussions, especially on its relevance to achieving global goals such as universal health coverage and sustainable development goals. |
| Define OOPE at first use. OOP is defined but not OOPE | This has been defined |
| It is not clear what problem this study seeks to address. Please elaborate on statement of the problem. Also, please what knowledge gap is it aiming to bridge? Further, linked to the problem statement, the justification for the study is weak, authors need to strengthen it. | The problem statement has been strengthened in the background, and the justification for the study. |
| Please include a paragraph on the structure of the Ugandan health system, indicating clearly service coverage and payment mechanisms, and levels of the service delivery structure that services under the NHI are provided, including where the private facilities stand in the scheme of things. Also, elaborate on the NHI in terms of focus, coverage, access etc. | The structure of the health system has been included in the methods under study settings.  In the background we have added details of the proposed NHI in terms of focus, who will access and be covered by the scheme. |
| Methods Authors wrote on page 9 lines 102 – 104 that this was a cross-sectional study design that involved mixed methods of data collection. Please indicate the type of mixed method study and how it was operationalized. | This was a concurrent mixed method and this has been specified |
| In describing the contingent valuation method (CVM), I think it would be appropriate for authors to weave in the relevance of this study and how it fits with the model. | The relevance of the study has been included |
| Authors have done a good job at describing the sampling technique used, but not how the FGD participants were selected. Please indicate the approach used? | How FGDs participants were purposively selected. This has been explained |
| Discussion I suggest authors summarize the key findings in beginning paragraph, then proceed to discuss. The discussion is haphazard and failed to properly situate the findings on the reasons for the WTP for the proposed NHI, ability to pay and NHI coverage within existing body of knowledge and contextual dynamics. Please review and properly structure the whole section. | The first paragraph summarizing findings has been added |
| In your discussion section, you must relate this to the international literature and point out the policy implications or implications for practice. | This has been emphasized |
| Please include a section on the study’s implication for policy and practice. | The implications for policy and practice have been added in the discussions at the end of each of discussion point. |
| Conclusion If WTP level is high, and the majority believed they could afford to pay for the health insurance, then I think there is the need for a strong conclusion and recommendations to make this study relevant in the scheme of things. In fact, there is no clear implication for policy. Please provide a clear and succinct but cogent conclusion based on your study findings – which may be inclusive of recommendations relevant for similar contextual settings. | This section has been strengthened with implications of the study findings and recommendations |
| General comments The paper will benefit from proofreading. There are a number of sentence construction issues and, incoherent statements and paragraphs that need attention. | We have proof read the paper and corrected the grammatical issues |

Thank you for your consideration.

Sincerely,

Noel Namuhani, Research associate, Makerere University School of Public Health
