# Supplementary material for: Willingness and perceived ability to pay for Uganda’s proposed national health insurance scheme among informal sector workers in Iganga and Mayuge districts, Uganda: A contingent valuation method
Source: PLOS Glob Public Health. 2026 Mar 11;6(3):e0003603. doi: 10.1371/journal.pgph.0003603 (PMC12978500; doi:10.1371/journal.pgph.0003603)
Supplement: Attachment — Submitted filename: Response to comments 13th May 2025.docx [file pgph.0003603.s005.docx]

**Date: 13th /May/2025**

**The Editor,**

PLOS Global Public Health

Dear Sir/Madam,

**Re:** **Address of Comments for the manuscript PGPH-D-24-01717R2**

Please, below is how we addressed the comment regarding potentially identifying information in the data set.

| **Comment** | **How it was addressed** |
| --- | --- |
| We note that there is potentially-identifying data in the Supporting Information file ‘Dataset-De identified.xlsx’ (specific location data). Therefore, we have hidden this file in the system to prevent it from being published mistakenly. Prior to sharing human research participant data, authors should consult with an ethics committee to ensure data are shared in accordance with participant consent and all applicable local laws. Data sharing should never compromise participant privacy. It is therefore not appropriate to publicly share personally identifiable data on human research participants. | Thank you for your observation. The data set has been fully de-identified and re uploaded as supporting information and legend provided in the manuscript. We removed columns containing identifying information such addresses, age, names. We have replaced the household codes with numbers so that these can not be traced. |

Thank you for your consideration.

Sincerely,

Noel Namuhani, Research associate, Makerere University School of Public Health
