# Supplementary material for: Willingness and perceived ability to pay for Uganda’s proposed national health insurance scheme among informal sector workers in Iganga and Mayuge districts, Uganda: A contingent valuation method
Source: PLOS Glob Public Health. 2026 Mar 11;6(3):e0003603. doi: 10.1371/journal.pgph.0003603 (PMC12978500; doi:10.1371/journal.pgph.0003603)
Supplement: Attachment — Submitted filename: Response to comments 8th July 2025.docx [file pgph.0003603.s006.docx]

**Date: 8th /July/2025**

**The Editor,**

PLOS Global Public Health

Dear Sir/Madam,

**Re:** **Address of Comments for the manuscript PGPH-D-24-01717R1**

We thank you and the reviewers for the time taken to review our manuscript. We appreciate all the comments and suggestions given. Below is how we attempted to address them and improve the paper.

| **Comment** | **How it was addressed** |
| --- | --- |
| Language editing | The entire manuscript has been edited. |
| Reviewer #2: The study aimed to explore the underlying factors influencing willingness and ability to pay for NHI in Uganda. The topic is relevant for health system reforms towards achieving UHC in Uganda. Authors have addressed the majority of key issues raised in my previous reviews. However, there are still a few issues requiring authors’ attention: | We appreciate the comments and the second review of our paper. We have looked into the persistent issues raised and addressed them as below. |
| Although authors have tried, I still think the discussion section is not sufficiently nuanced in a manner that will situate in global body of knowledge for international relevance. Also, the statement included implication of the study results for policy and practice woefully superficial. I suggest authors elaborate to include actionable recommendations based on the study findings, targeted at various levels of Uganda’s health systems. | A sub section on implications for policy is included where we highlight some of the actionable recommendations (Page 26). The entire discussions have been revised. |
| In conclusion, results is still being presented and discussed/interpreted, instead of making concluding remarks based on the interpreted results. I believe the conclusion could still be strengthened. | *This has been rewritten. We have mimized repeating the results. Although it is a bit challenging to give concluding remarks without giving an overall picture of our results and how we interpret them.* |
| Review #3 |  |
| Reviewer #3: Avoid unnecessary capitalization in the heading. Do not use willingness to pay and WTP concurrently. Resort to the acronym after first use. OOPE was not defined on first use. Revise that. | *The overall grammar has been checked.*  *The capitalizations have been dealt with in the subheadings.*  *Acronyms have been used for WTP, and other after first defining on first mention.*  *OOPE has been defined on first use in the abstract* |
| Contrast the following statement with the burden of diseases in these countries and how that creates a paradox. "According to the World Bank, direct modes of payment for54 health care services constitute 40% of health care expenditure in developing countries (4) | *This has been done. Thanks* |
| The information on page 10, lines 163 to 166, is repetitive | *This has been deleted.* |
| How was the semi-structured questionnaire developed? Adapted/adopted? Often, researchers have problems integrating results in mixed-method studies. What type of mixed-methods design was employed? How did that influence the integration of both quantitative and qualitative data? | *The tool was developed by the authors and this has been indicated.*  *The type of mixed methods study had already been indicated under the section of study design. For emphasis, this was convergent parallel where quantitative and qualitative data were collected simultaneously and hence we present the findings co currently too. This has also been indicated under the section of data collection.* |
| . Table 2: The classification of the religion is confusing. Catholicism, Protestantism, etc. are not religions but denominations or variants within the Christian fraternity. | *According to Uganda Bureau of statistics, that is how religion is classified. However, to avoid confusion to the global audience, we have changed the subheading to religious denominations. This has been changed in table 2.* |
| Could you correct this error?Line 264-266: "gave WTP zero" is grammatically incorrect. Should be "had a zero WTP." | *Thank you. We have changed this.* |

We hope these revisions fully address your concerns and strengthen the manuscript's clarity, relevance, and contribution to literature.

Thank you for your consideration.

Sincerely,

Noel Namuhani, Research associate, Makerere University School of Public Health
