# Supplementary material for: Willingness and perceived ability to pay for Uganda’s proposed national health insurance scheme among informal sector workers in Iganga and Mayuge districts, Uganda: A contingent valuation method
Source: PLOS Glob Public Health. 2026 Mar 11;6(3):e0003603. doi: 10.1371/journal.pgph.0003603 (PMC12978500; doi:10.1371/journal.pgph.0003603)
Supplement: Attachment — Submitted filename: Response to Reviewers 11th September 2025.docx [file pgph.0003603.s008.docx]

**Date: 11th /September/2025**

**The Editor,**

PLOS Global Public Health

Dear Sir/Madam,

**Re:** **Address of Comments for the manuscript PGPH-D-24-01717R3**

Please, below is how we have addressed the reviewers’ comments. Please, note that we included one more citation/reference in the methodology (reference number 24).

| **Comment** | **How it was addressed** |
| --- | --- |
| 1. If the authors have adequately addressed your comments raised in a previous round of review and you feel that this manuscript is now acceptable for publication, you may indicate that here to bypass the “Comments to the Author” section, enter your conflict of interest statement in the “Confidential to Editor” section, and submit your "Accept" recommendation.  Reviewer #2: All comments have been addressed  Reviewer #3: All comments have been addressed | Great. We appreciate both reviewers for their time and suggestions. The comments really strengthened this paper. |
| 2. Does this manuscript meet PLOS Global Public Health’s publication criteria? Is the manuscript technically sound, and do the data support the conclusions? The manuscript must describe methodologically and ethically rigorous research with conclusions that are appropriately drawn based on the data presented.  Reviewer #2: Yes  Reviewer #3: Partly | Thanks to both reviewers. All the authors have reviewed the entire manuscript to ensure it fits the journals publication criteria. |
| 3. Has the statistical analysis been performed appropriately and rigorously?  Reviewer #2: Yes  Reviewer #3: Yes | Thank you so much |
| 4. Have the authors made all data underlying the findings in their manuscript fully available (please refer to the Data Availability Statement at the start of the manuscript PDF file)?The PLOS Data policy requires authors to make all data underlying the findings described in their manuscript fully available without restriction, with rare exception. The data should be provided as part of the manuscript or its supporting information, or deposited to a public repository. For example, in addition to summary statistics, the data points behind means, medians and variance measures should be available. If there are restrictions on publicly sharing data—e.g. participant privacy or use of data from a third party—those must be specified.  Reviewer #2: Yes  Reviewer #3: Yes | Thank you |
| 5. Is the manuscript presented in an intelligible fashion and written in standard English? PLOS Global Public Health does not copyedit accepted manuscripts, so the language in submitted articles must be clear, correct, and unambiguous. Any typographical or grammatical errors should be corrected at revision, so please note any specific errors here.  Reviewer #2: Yes  Reviewer #3: No | The three authors have reviewed the entire manuscript to address any grammatical errors. We have tried to address these. |
| 6. Review Comments to the Author  Please use the space provided to explain your answers to the questions above. You may also include additional comments for the author, including concerns about dual publication, research ethics, or publication ethics. (Please upload your review as an attachment if it exceeds 20,000 characters)  Reviewer #2: Authors have addressed all comments and provided clarity to the queries I raised in my last review. However, I recommend that authors proofread the entire paper as there are a few sentence construction issues.  Reviewer #3: Improve writing style. | Thank you. We have proof read the paper |

Thank you for your consideration.

Sincerely,

Noel Namuhani, Research associate, Makerere University School of Public Health
