# Supplementary material for: Willingness and perceived ability to pay for Uganda’s proposed national health insurance scheme among informal sector workers in Iganga and Mayuge districts, Uganda: A contingent valuation method
Source: PLOS Glob Public Health. 2026 Mar 11;6(3):e0003603. doi: 10.1371/journal.pgph.0003603 (PMC12978500; doi:10.1371/journal.pgph.0003603)
Supplement: Attachment — Submitted filename: PLOS Global Public Health WTP Uganda NHIS 3 Nov 2025.docx [file pgph.0003603.s007.docx]

PLOS Global Public Health

Willingness and perceived ability to pay for Uganda’s proposed national health

insurance scheme among informal sector workers in Iganga and Mayuge districts,

Uganda: a contingent valuation method

| Comment | Reference |
| --- | --- |
| What was the response rate? | Line 28 |
| Please clarify, were all 853 respondents to the survey interviewed? | Line 51 |
| I would suggest including literature or data on the amount of OOPE being spent by ordinary Ugandans and their impact in terms of catastrophic health expenditures and impoverishment. This could help contextualize the reducing share of OOPE from the national health accounts. | Line 75 |
| Are there already studies on the WTP of the formal sector in Uganda? Perhaps it would be good to include this in your review or discussion section? | Line 119-120 |
| Could you clarify what the total population for mayuge district is? There appears to be a typo stating it as 473,2394 | Line 151 |
| Were more modern methods for calculation considered in this study? For example, this paper by Vaughan, Russell, and Darling in 2000 discussed an approach to optimizing sample sizes for surveys to conduct contingent valuation?  What would the impact have been if more recent sample size calculation methods were used?  Conversely, what are the particular benefits of using kish’s formula for this study? | Line 161 |
| In table 1, I suggest adding the total population for each district that is being sampled | Line 186 |
| In row 4 table 1 what is the number being multiplied to the proportion of the population subcategory? For instance, in Iganga 65% are farmers/peasants and this proportion was multiplied by 440? What does the 440 number represent? | Line 186 |
| It appears I could not find a response rate for the study.  It seems improbable that the calculated sample size was 853, and there was a 100% response rate, with no exclusions or refusals to be interviewed for the survey.  Please explain if there were instances respondents declined or were unable to participate, or whether certain criteria were applied to exclude a respondent. | Line 262 |
| Why was the derived WTP from the study compared to Iran, Namibia and Kenya? | Line 377 |
| Developing a savings group seems to be a promising strategy, perhaps looking for more literature to look at comparable countries to Uganda who have implemented this would be useful to help discuss the results in addition to the Pakistan reference? | Line 387 |

As it is, I think the paper is informative and useful. Inclined to ask for minor revisions to clarify response rate question, clarifications in the sample size computation, and including more literature to help support findings being discussed.
