# Supplementary material for: Willingness and perceived ability to pay for Uganda’s proposed national health insurance scheme among informal sector workers in Iganga and Mayuge districts, Uganda: A contingent valuation method
Source: PLOS Glob Public Health. 2026 Mar 11;6(3):e0003603. doi: 10.1371/journal.pgph.0003603 (PMC12978500; doi:10.1371/journal.pgph.0003603)
Supplement: Attachment — Submitted filename: Respense to Reviewers comments 10th January 2026 .docx [file pgph.0003603.s009.docx]

Date: 21^st^ /January/2026

**The Editor,**

PLOS Global Public Health

Dear Sir/Madam,

Re **Address of Comments for the manuscript PGPH-D-24-01717R4**

We thank you and the reviewers for the time taken to review our manuscript. We appreciate all the comments and suggestions given. Below is how we attempted to address them and improve the paper.

| **Comment** | **Reference** | **Response** |
| --- | --- | --- |
| What was the response rate? | Line 28 | We selected participants untiled we reached the targeted sample size of 853. For the 17 respondents who were not available at the time of study, these were replaced until we achieved our targeted sample size. This has been clarified in the methods. |
| Please clarify, were all 853 respondents to the survey interviewed? | Line 51 | we sampled until the calculated sample size was reached. 17 respondents were not available at the time of survey; these were replaced to achieve our targeted sample size of 853. |
| I would suggest including literature or data on the amount of OOPE being spent by ordinary Ugandans and their impact in terms of catastrophic health expenditures and impoverishment. This could help contextualize the reducing share of OOPE from the national health accounts. | Line 75 | We have added the amount and catastrophic health expenditure levels. |
| Are there already studies on the WTP of the formal sector in Uganda? Perhaps it would be good to include this in your review or discussion section? | Line 119-120 | we have added WTP for CBHI and general insurance for the general population. Noting the gap in evidence on WTP for the proposed scheme and informal sector to be specific in Uganda at the time of the study. |
| Could you clarify what the total population for mayuge district is? There appears to be a typo stating it as 473,2394 | Line 151 | Thanks, the figure had a typo. The population estimate was 473,239. This has been corrected in the document |
| Were more modern methods for calculation considered in this study? For example, this paper by Vaughan, Russell, and Darling in 2000 discussed an approach to optimizing sample sizes for surveys to conduct contingent valuation?  What would the impact have been if more recent sample size calculation methods were used?  Conversely, what are the particular benefits of using kish’s formula for this study? | Line 161 | We acknowledge the need to use the advanced modern methods for sample size calculations. Which could be more rigorous. However, since our main outcome was taken to be binary, a proportion, Kish Leslie was appropriate to yield the estimated sample size. Additionally, the Russel approach requires known estimates of WTP, which we did not have at the time of designing the study. However, we have noted that under limitations. |
| In table 1, I suggest adding the total population for each district that is being sampled | Line 186 | These have been added, in now table 2 |
| In row 4 table 1 what is the number being multiplied to the proportion of the population subcategory? For instance, in Iganga 65% are farmers/peasants and this proportion was multiplied by 440? What does the 440 number represent? | Line 186 | This figure is the calculated sample size for the district. The sample size for each district was estimated proportionate to size. For instance, Iganga with a total population of 504,197 (51.6%), thus the sample =853*0.516 =440. And for Mayuge 473,239 (48.4%). =853*0.484=413  A table (1) has been added to illustrate the sample size for each district. Under the sample size calculations. |
| It appears I could not find a response rate for the study.  It seems improbable that the calculated sample size was 853, and there was a 100% response rate, with no exclusions or refusals to be interviewed for the survey.  Please explain if there were instances respondents declined or were unable to participate, or whether certain criteria were applied to exclude a respondent. | Line 262 | We thank the reviewer for raising this point. We clarify that the reported sample size of 853 refers to the final achieved sample, rather than the number of individuals initially approached. Community leaders supported mobilization efforts, which helped minimize refusals. Of the sampled participants, 17 households were unavailable at the time of data collection. In these cases, an eligible replacement was systematically selected from the same sampling unit, until the required sample size was achieved. These details have been added in the methods to clarify |
| Why was the derived WTP from the study compared to Iran, Namibia and Kenya? | Line 377 | These were the available similar studies at that time. Countries like Kenya are comparable to Uganda. Iran has a strong universal health insurance scheme, thus good to see how that compares with our findings. |
| Developing a savings group seems to be a promising strategy, perhaps looking for more literature to look at comparable countries to Uganda who have implemented this would be useful to help discuss the results in addition to the Pakistan reference? | Line 387 | Thanks for your reflections. We have cited cases from Kenya and Cameroon. |

Thank you for your consideration.

Sincerely,

Noel Namuhani, Research associate, Makerere University School of Public Health
