# Supplementary material for: Willingness and perceived ability to pay for Uganda’s proposed national health insurance scheme among informal sector workers in Iganga and Mayuge districts, Uganda: A contingent valuation method
Source: PLOS Glob Public Health. 2026 Mar 11;6(3):e0003603. doi: 10.1371/journal.pgph.0003603 (PMC12978500; doi:10.1371/journal.pgph.0003603)
Supplement: Attachment — Submitted filename: Respense to Reviewers comments 20th Feb 2026.docx [file pgph.0003603.s010.docx]

Date: 20^th^ /February/2026

**The Editor,**

PLOS Global Public Health

Dear Sir/Madam,

Re **Address of Comments for the manuscript PGPH-D-24-01717R5**

We thank you and the reviewers for the time taken to review our manuscript. We appreciate all the comments and suggestions given. Below is how we attempted to address them and improve the paper.

| **Comment** | **Response** |
| --- | --- |
| While the sample size was calculated to be 853, it’s unclear if the authors achieved a 100% response rate. Were there any errors identified during data cleaning, or were there any observations dropped due to errors? It’s important to clarify whether all 853 individuals were invited and interviewed. This information should be included in the data analysis section of the manuscript. | We have added clarifying statement in the analysis section as below: A total of 853 informal sector workers were randomly selected and interviewed, achieving a 100% response rate. To achieve this target, 17 participants who were unavailable at the time of the survey were replaced. All completed surveys were included in the analysis, and no observations were dropped due to errors or missing data. Data were checked thoroughly in the field to identify and address any missing information |
| Additionally, in the abstract, you mentioned that a total of 853 informal sector workers were randomly sampled. It’s crucial to specify if they were simply sampled or if they were both sampled and interviewed. Did all 853 participants take part in the survey? Please make this clear in your abstract. | this has been addressed in the abstract as below: A total of 853 informal sector workers, including farmers, commercial motorists, fisherfolk, and traders, were randomly selected and all interviewed. Seventeen households that were unavailable at the time of data collection were replaced to achieve the target sample size, resulting in a 100% response rate |
| Lastly, I noticed some words highlighted in different colors and font sizes throughout the manuscript. Please address these inconsistencies, specifically: - The reference to "forms to the ODK server" found in the second paragraph of the data collection section. - Table 5, which discusses multivariable factors under wealth quintiles. Also, kindly ensure that all comments are removed. | These inconsistences have been addressed. Thank you |

Thank you for your consideration.

Sincerely,

Noel Namuhani, Research associate, Makerere University School of Public Health
